# The Vlasov bivector: a parameter-free approach to Vlasov kinematics

**DOI:** 10.1140/epjp/s13360-026-07703-8

**Published:** 2026-05-07

**Authors:** Finlay Gunneberg, Jonathan Gratus, Harvey Stanfield

**Affiliations:** 1 Department of Physics, Lancaster University, Lancaster, LA1 4YB UK; 2 The Cockcroft Institute, Daresbury Labs, Keckwick Ln, Daresbury, WA4 4AD UK; 3 Department of Physics & Astronomy, Manchester University, Manchester, M13 9PL UK

## Abstract

Plasma kinematics is typically performed on either the mass shell or a lab bundle, seven-dimensional phase spaces equipped with a Vlasov vector field. This choice of phase space encodes the parametrisation of the differential equations governing particle dynamics. By replacing the Vlasov vector field and related quantities over a phase space with a bivector on an eight-dimensional conic sub-bundle of the tangent bundle, we construct a parameter-free version of Vlasov theory. The advantages of this formalism include compatibility with light-like and ultra-relativistic particles, non-metric connections, and metric-free or pre-metric theories. Additionally, this formalism applies to theories where no time phase space can exist for topological reasons, e.g. when we wish to consider all geodesics, including space-like geodesics. We also use this formalism to derive a simple formula which enables the transformation of a Vlasov field from one phase space to another in such a way that the trajectories of the particles in the base space are unchanged. Our formalism also has implications for numerical simulations. By extending quantities such as the particle density form onto an eight-dimensional conic sub-bundle, we can extend existing numerical integrators, such as the agile numerical integrator, to relativistic scenarios. The extension of the particle density form also allows for the generalisation of the current 3-form onto the conic bundle. We also discuss the complicated relationship between the stress–energy form and phase space, how our formalism illustrates this, and the corresponding challenges in developing a parameter-free Einstein–Vlasov system. The relationship between our formalism and sprays or semi-sprays is explored, and examples from Finsler geometry are given.

## Introduction

In Vlasov and Boltzmann theories of kinematics, one is interested in the dynamics of a scalar field over a time phase space which represents a particle density. The fields are typically functions of time, 3 positional coordinates, and 3 velocity or momentum coordinates. The dynamic equations are written in terms of a first-order operator on this scalar field. In the case of the Vlasov equation, the action of the operator on the scalar field is zero, whereas for the Boltzmann equation, the right-hand side is nonzero to account for collisions. This seven-dimensional time phase space, which we call the *kinematic domain*, is a subspace of the eight-dimensional tangent bundle. It corresponds to the chosen parameterisation one uses for the underlying solutions of the chosen force equation. Sometimes there is a natural choice for this seven-dimensional space, for example the mass shell in the case of an Einstein–Vlasov system (see [1]). In other cases, it has to be chosen, e.g. for null geodesics. Furthermore, there are cases where it is not even possible to construct a kinematic domain, for example, in the case where we consider all geodesics, including space-like geodesics.

**Fig. 1:**
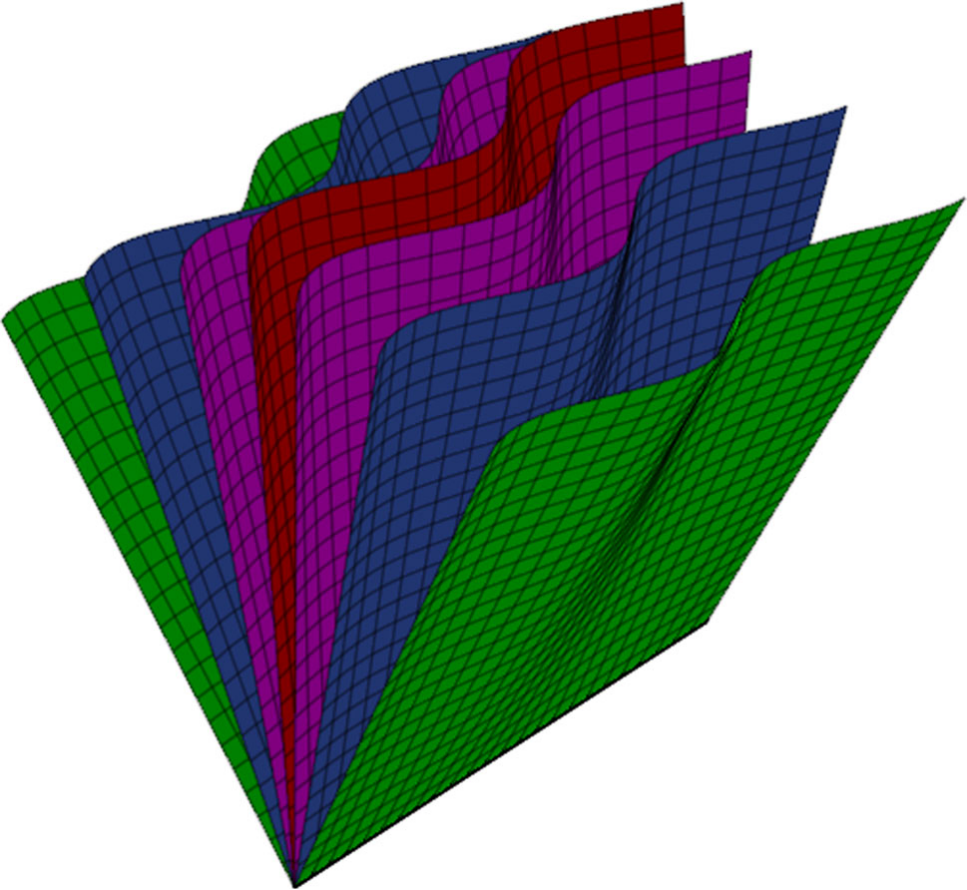
The Vlasov Bivector integrates to form leaves, like the pages of a book. The density of the leaves also corresponds to the particle distribution

In this article, we investigate an alternative approach that does not require a choice of kinematic domain. We work on an eight-dimensional conic bundle, which is a subset of the tangent bundle, and we replace the Vlasov vector field with the Vlasov bivector. This corresponds to not choosing a parameterisation for the underlying ordinary differential equations (ODEs) of the system. The integral two-dimensional surfaces of this bivector are depicted in Fig. 1. There are many advantages to this formalism which we discuss in the following subsection. Although we concentrate on the geodesic or Lorentz force equations and their corresponding Vlasov fields, this approach can be applied to the kinematics models for any second-order ODEs, on any arbitrary dimensional base manifold.

In this article, we first summarise the standard kinematic domain approach plus its relationship with the conic sub-bundle, and make the link with sprays and semi-sprays. This includes the means by which we transform a Vlasov field between kinematic domains. We then define the Vlasov bivector and particle density 6-form, and we give the equations of motion for the latter. We show how to go between the approaches, when the kinematic domain approach exists. We also show how to calculate the current 3-form, and the analogue to the stress–energy tensor, needed for the Einstein–Vlasov system. Next, we discuss the implications of our formalism for numerical methods and apply our formalism to extend the agile numerical integrator (ANI) to relativistic contexts.

In this article, we use sprays and Finsler geometry as examples to put this work in context. However, for the reader unfamiliar with these concepts, all statements about sprays, semi-sprays, and Finsler geometry may be safely ignored. The details linking our work and sprays are given in Sect. A.1.

### The standard Vlasov approach

**Fig. 2:**
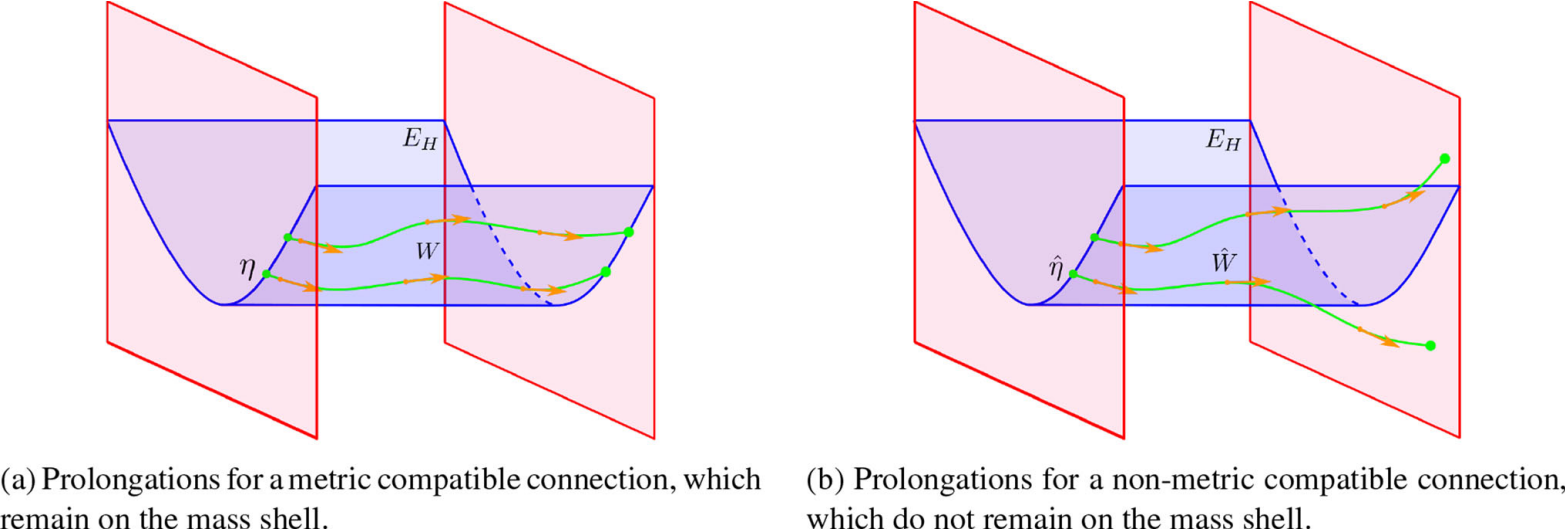
Illustration of integral curves, i.e. the prolongations, in the mass shell for the case of a Vlasov field built from a force equation with a metric compatible connection *W* (Fig. 2a), and a non-metric compatible connection $\hat{W}$ (Fig. 2b). The mass shell is represented by the blue sheet, integral curves $\eta ,\;\hat{\eta }$ of the Vlasov fields $W,\hat{W}$ are given by the green lines. The Vlasov fields themselves are depicted by the orange arrows

When performing Vlasov kinematics, a seven-dimensional time phase space *E* is chosen upon which to construct the particle density scalar field $f_E$. We will refer to *E* as the kinematic domain, and it is a bundle over the spacetime manifold *M*. The first-order partial differential equation governing the dynamics of $f_E$ can be represented by the Vlasov vector field $W_E\in \Gamma TE$,

1 $$\begin{aligned} W_E\langle f_E\rangle =0, \end{aligned}$$

where the angled brackets $\langle \bullet \rangle$ indicate the action of a vector field on a scalar field. Note that it is also referred to as the Liouville vector field in some literature, e.g. [2].

Throughout this article, we use the language of differential geometry on the tangent bundle to describe Vlasov kinematic models. For an overview of this technique, see [3]. The solutions to the underlying second-order ordinary differential equations (ODEs) are called trajectories and correspond to the worldlines of the particles. The tangent vectors to the trajectories, that is, the velocities of particles, are curves in the kinematic domain, called the prolongation of the trajectories. These prolongations are the integral curves of the Vlasov vector field.

**Fig. 3:**
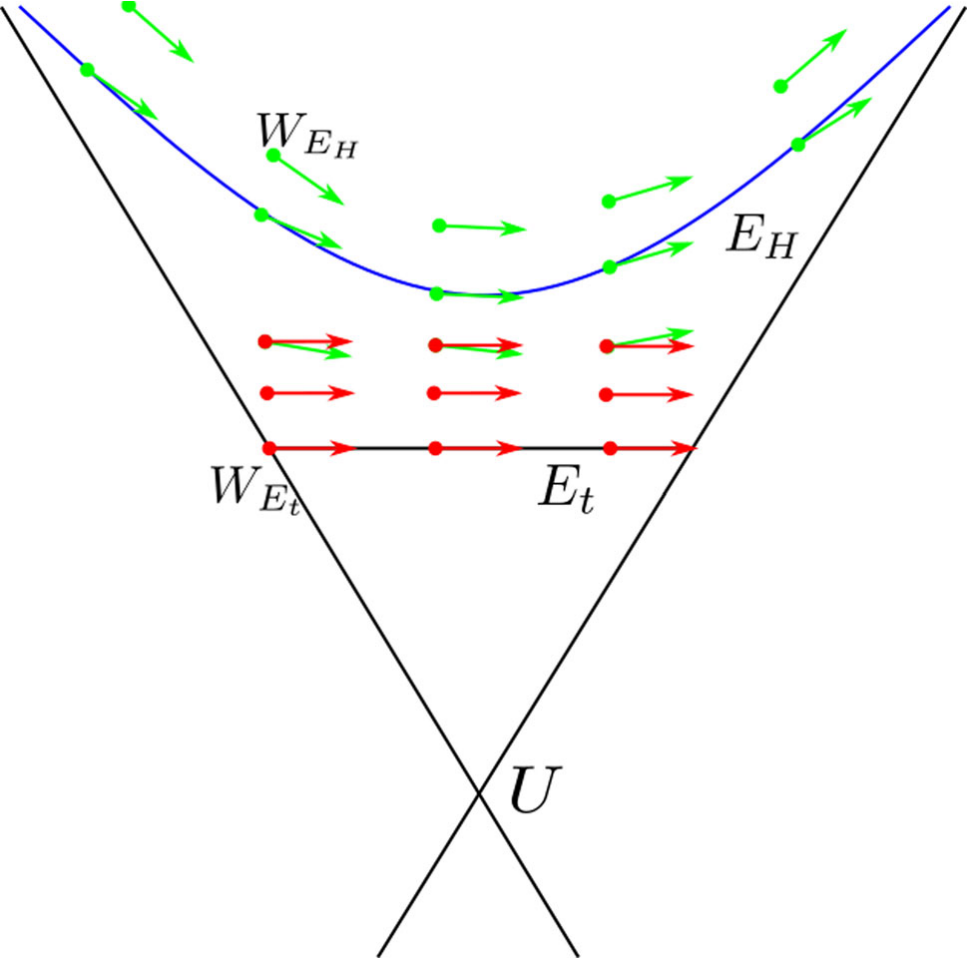
The seven-dimensional kinematic domains $E_{\text {H}}$ and $E_{t}$ are given by slices of the eight-dimensional conic bundle $U\subset TM$. The unit hyperboloid $E_{\text {H}}$ is given by the dark blue hyperbola and a lab-time bundle $E_{t}$ by the horizontal black line. The Vlasov fields $W_{E_{\text {H}}}$ and $W_{E_{t}}$ (green and red arrows, respectively) are tangent to their respective kinematic domains

The kinematic domain $E\subset TM$ is a sub-bundle of the tangent bundle over the base manifold *M*, and there are many choices for this sub-bundle. For example, if *M* has a spacetime metric, *g* [with signature $(-,+,+,+)$], then one choice is to use the unit mass shell $E_{\text {H}}$, also known as the upper unit hyperboloid:

2 $$\begin{aligned} E_{\text {H}}=\{ \underline{v}\in TM :g(\underline{v},\underline{v})=-1, \underline{v} \text{ is } \text{ future } \text{ pointing } \}. \end{aligned}$$

This corresponds to proper-time parameterisation and is the natural choice for the relativistic Vlasov–Maxwell system [4] and the Einstein–Vlasov system [1]. When performing plasma kinematics with the Vlasov–Maxwell system, we typically formulate the Lorentz force equation in terms of a metric compatible connection. One disadvantage of the upper unit hyperboloid is that prolongations of trajectories for a non-metric compatible connection will not, in general, remain on the mass shell (see Lemma A.6 for an example). This is visualised in Fig. 2.

Another choice of kinematic domain is the lab-time bundle, $E_{t}$ where $t\in \Gamma \Lambda ^0M$ is a lab time. This *t* has the property that if $\underline{v}$ is future pointing then $\underline{v}\langle t\rangle >0$. We also refer to *t* as a time-slicing scalar, because it slices spacetime into hypersurfaces. The events on each hypersurface are designated to be simultaneous. The term lab time refers to any scalar field *t*, not necessarily the time coordinate of an inertial frame or associated with a lab. Thus, it can also refer to the rest frame of an accelerating particle. The lab-time bundle, $E_{t}$, consists of vectors $\underline{v}$ such that $\underline{v}\langle t \rangle =1$ subject to some additional restrictions, such as being time-like. The lab-time bundle is defined in Eq. (21), once we have defined the conic bundle, *U*.

Working on $E_{t}$ adds an additional term to the Vlasov equation relative to the Vlasov field on $E_{\text {H}}$. Unlike the unit hyperboloid, it does not require a metric compatible connection. It is also useful when other quantities are defined with reference to a global time (see, for example, [5]). The disadvantage of this approach is that one has to choose a lab-time coordinate and there may be complicated transformations from one lab-time coordinate to another. An illustration of kinematic domains as ‘slices’ of a conic bundle is given in Fig. 3.

Given two kinematic domains, say *E* and $\hat{E}$, it is possible to transform the Vlasov field $W_E\in \Gamma TE$ to another Vlasov field $W_{\hat{E}}\in \Gamma T\hat{E}$ in such a way that the trajectories in the base space are unaffected. This transformation corresponds to a reparameterisation of the trajectories associated with the Vlasov field. For example, the geodesic equation on the unit hyperboloid $E_{\text {H}}$ becomes the pre-geodesic equation on the lab-time bundle $E_{t}$, and by analogy, the Lorentz force equation becomes the pre-Lorentz force equation. This extra force term, which is always proportional to the velocity, gives rise to an extra term in the Vlasov field.

### Motivation for the parameter-free approach

The primary goal of this work is to present the formalism of the Vlasov field and the corresponding Vlasov equation in a way which does not require choosing a kinematic domain, or equivalently, a parameterisation. It is a considerable abstraction to pass from the seven-dimensional kinematic formalism to the eight-dimensional formalism, and several new concepts must be defined, such as the Vlasov bivector and the particle density form. We identify here a number of reasons why this new formalism is justified.

- It removes the arbitrariness of the kinematic domain and the need for additional terms. Thus, this approach is fundamentally free of the choice of parameterisation.
- It works when modelling light-like particles where the kinematic domain is a $(2n-2)$-dimensional bundle over a *n*-dimensional manifold, and hence, one cannot use the hyperboloid bundle, $E_{\text {H}}$. One could use a lab-time bundle $E_{t}=\{ \underline{v}\in TM :\underline{v}\langle t \rangle =1\text { and } g(\underline{v},\underline{v})=0\}$. In the case of Minkowski spacetime, with *t* given by the usual Minkowski time coordinate, the prolongations of light-like geodesics remain on $E_{t}$. However, in general where there is gravity or where we use an arbitrary time scalar field, this is not the case, and the prolongations of light-like geodesics will not remain on $E_{t}$. See Appendix Lemma A.7 for an example of this. Thus, one has two choices, either to use our approach given here or to use the pre-geodesic equation.
- In particle accelerators, charged particles travel at speeds very close to the speed of light, and one can use an ultra-relativistic approximation. Although massless particles are not affected by electromagnetic fields, ultra-relativistic particles do respond in the limit where the electromagnetic field becomes infinite. In [6], the authors consider the ultra-relativistic approximation for a charged fluid. The approach given here will enable the ultra-relativistic approximation for a kinetic description of charged particles.
- Sometimes it is necessary to work in multiple kinematic domains for practical reasons, and hence, it is necessary to transform the Vlasov equation between them. One can construct this transformation by considering the underlying ODEs, rescaling them, and then reconstructing the new corresponding Vlasov equation. The advantage of starting with the parameterisation-free approach is that it gives the formula for this transformation directly. This can be visualised in Fig. 3. This is analogous to the advantages of working in coordinate-free notation. If one is subsequently given a coordinate system, one can easily calculate the corresponding coordinate quantities from the coordinate-free quantities. Also, given two coordinate systems, the transformation of these coordinate quantities is also derived from the coordinate-free definitions. Likewise the formula for passing from one kinematic domain to another falls out of our kinematic domain-free definition of the Vlasov field. Additionally, working with coordinate-free tools often provides geometric insight (see [7], for example).
- The numerical simulation of plasmas, both terrestrial and astrophysical, is of intensive interest. For these, one may attempt to solve the Maxwell–Vlasov system directly [8] (especially in 1 or 2 dimensions) or use a particle-in-cell (PIC) code. There are many codes in current use [9]. These generally use a lab frame [10] which updates all the fields and particles from one time steps to the next. These time steps define the time-slicing scalar field, that is, a field on spacetime whose contours define all simultaneous events. We know from relativity that such a notion of simultaneity is not universal and is a choice. When one can ignore the effect of gravity, it is usual to use an inertial frame. In Sect. 5.2, we leverage our formalism to generalise the agile numerical integrator (ANI), found in [11]. We generate the equations for a Lorentz boost of the one-dimensional Vlasov–Poisson equation. The current applications of the ANI are to systems where the velocities are either non-relativistic or only mildly relativistic. This avoids velocities bunching just below $v=c$. This prevents the ANI being used to model a bunch of particles being accelerated to ultra-relativistic velocities, such as in a particle accelerator. Using a series of Lorentz boosts, one can model such bunches. Here, we constantly work in a frame where the velocities have $v\ll c$, even though the actual velocities, relative to the lab, are ultra-relativistic. One could also use an arbitrary non-inertial frame, for example, one adapted to a prescribed accelerating orbit. This is a natural thing to do when considering moments of a distribution, for example, [5]. Our technology will trivially give you the Vlasov system in this case. Starting with the adapted time-slicing scalar field, it is straightforward to use Eq. (54) to deduce the Vlasov field in this adapted coordinate system. An alternative would require calculating all the pseudo-forces in the equations of motion and constructing the Vlasov field from these. The same approach can be used in general relativity with the contours of the time-slicing scalar field being the geodesic hypersurfaces which are orthogonal to a prescribed worldline [12].
- In this formalism, we work on the conic bundle $U\subset \breve{T}M$ (where $\breve{T}M=TM\backslash \{0\}$, the subset of *TM* which excludes the zero vectors), which is a bundle over *M* and has the same dimension as *TM*. The formal definition of the conic bundle is given in Definition 1.5. There are cases where there cannot exist a kinematic domain for topological reasons. For example, suppose we wish to consider all solutions to the autoparallel equation. In spacetime, this corresponds to all time-like, light-like and space-like geodesics simultaneously. In this case, $U=\breve{T}M$. Thus, we would have to pick an initial velocity for each direction in order to construct the phase space *E* upon which we define our Vlasov field. Each fibre $E_p$ would then be topologically equivalent to the quotient set $T_pM/\sim$ where $\underline{v}\sim \lambda \underline{v}$ for $\lambda \ne 0$. This set is the real projective $(n{-}1)$-space $\mathbb {R}P^{n-1}$ in the case where *M* is a *n*-dimensional spacetime manifold. However, if *n* is odd, $n\ge 3$, $\mathbb {R}P^{n-1}$ cannot be embedded into $T_pM$, and hence, no kinematic domain *E* exists. By contrast, our formalism remains valid in this case. For proof that $\mathbb {R}P^{2n}$ cannot be embedded into $\mathbb {R}^{2n+1}$ see cor. 8.9 [13].
- As shown in Fig. 2, the prolongations of solutions to the Lorentz force equation do not, in general, remain on $E_{\text {H}}$ if the connection is not metric compatible. An example is given in Lemma A.6. By contrast, our approach works with force equations constructed from any connection and more general models of acceleration [14]. Furthermore, our approach works even when the manifold does not possess a metric. For example, using the autoparallel equations, one can construct the Vlasov equations with just a connection and no metric. Thus, it is compatible with pre-metric formalisms of dynamics (see [15–17]). For electrodynamics, without a metric, one would need to consider a force tensor, analogous to the electromagnetic field, but which maps vectors to vectors.
- This formalism can be generalised to Finsler spacetimes [18]. Furthermore, our formalism is not intrinsically dependant on any geometric objects beyond a base manifold. By casting objects from Finsler geometry-based kinematic theories (e.g. [19]), the dependence of these objects upon the Finsler metric can be better highlighted in a way analogous to pre-metric electromagnetism.
- The clock hypothesis is defined using the metric. Particles which decay must have a notion of time. However, for stable particles, one can argue that the description of the particles motion should not be defined in terms of proper time which does not affect the particles. Our approach may be useful for treatments which do not impose the clock hypothesis, e.g. Mashoon electrodynamics [20–23].
- Finally, there is a philosophical argument. There is vague distinction between the kinematics and the dynamics of a system. In the case of particle dynamics, the kinematics simply state that we are interested in curves that satisfy some unspecified ODE, whereas the dynamics prescribe the ODE. It is the dynamics that require the connection, and maybe a metric. By contrast, for the standard Vlasov field on $E_{\text {H}}$, the kinematics are defined on a kinematic domain which requires a metric. Thus, the metric is introduced at the kinematic stage rather than the dynamics stage.

### The Vlasov bivector and the transport equations

**Fig. 4:**
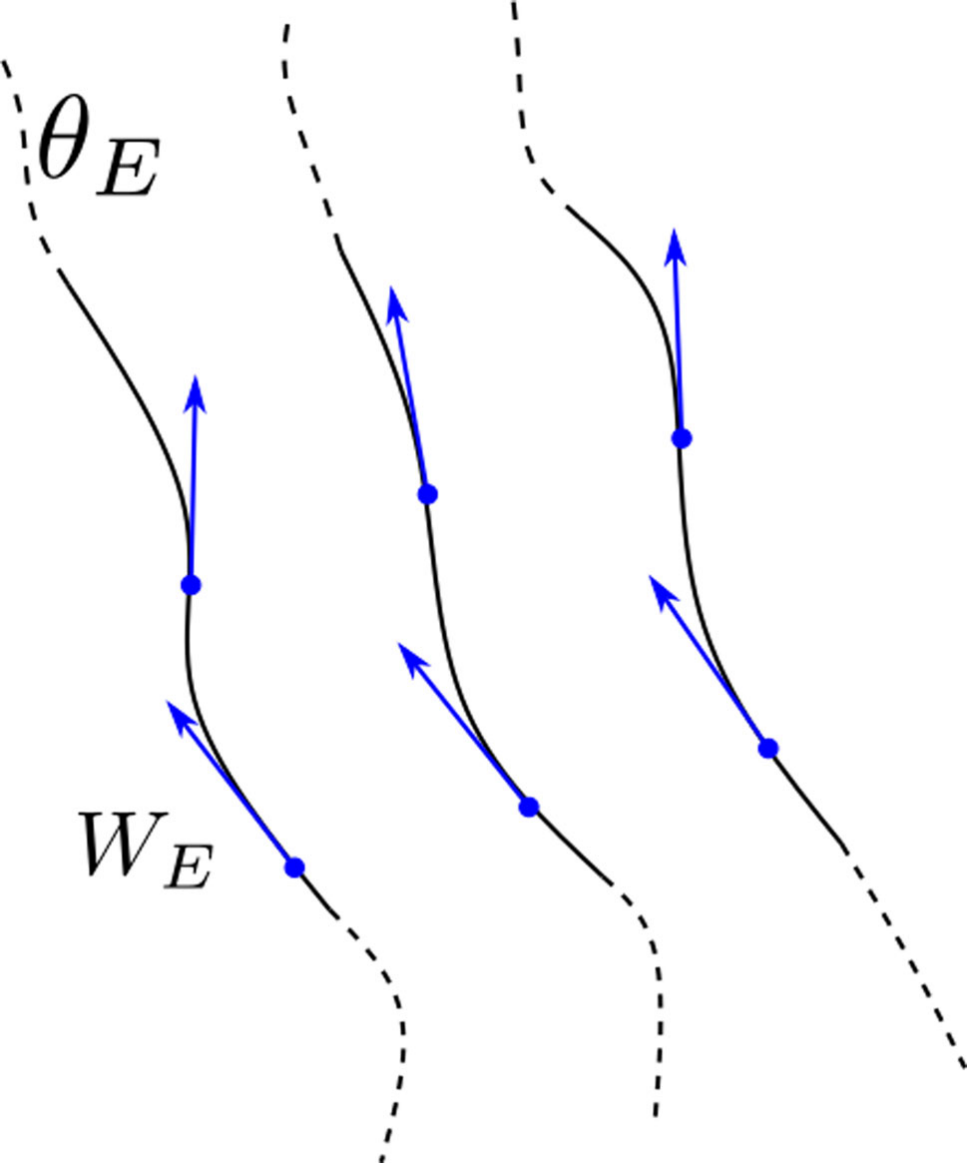
Diagram of the transport equations on a $(2n-1)$-dimensional kinetic domain *E* using form submanifolds. The details of submanifolds are described in [24]. The form manifolds of $\theta _E\in \Gamma \Lambda ^{2n-2} E$ (black lines) do not terminate ($d\theta _E=0$) and are tangent to the vector field $W_E\in \Gamma TE$, represented by blue arrows ($i_{W_E}\theta _E=0$)

**Fig. 5:**
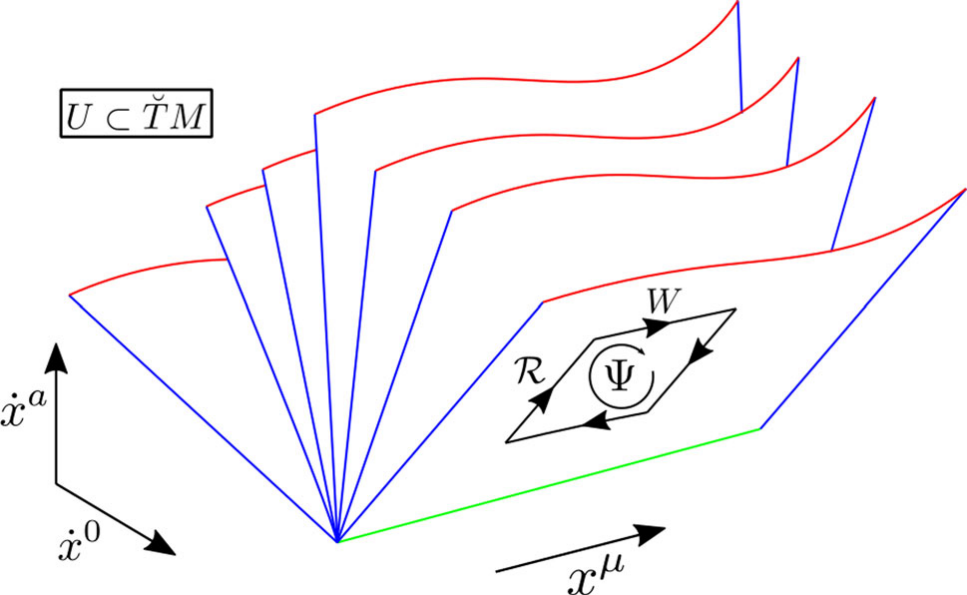
Sketch of an integrable Vlasov bivector $\Psi$. Here, a possible form for $\Psi$ is $\Psi =\mathcal {R}\wedge W$. Notice that since $\Psi$ is integrable, the bivectors ‘knit together’ to form the leaves of a foliation, also depicted in Fig. 1. The ambient space is $U\subset \breve{T}M$. The green line is to indicate the absence of 0-vectors in our space. The density of the leaves corresponds to the velocity density of our one particle distributions function with higher density towards the middle and lower density towards the sides. Viewing this diagram as the particle density form $\theta$, then the observation that the leaves are tangent to $\Psi$ and that they have no boundary, is equivalent to the transport equations given in Eq. (86)

**Fig. 6:**
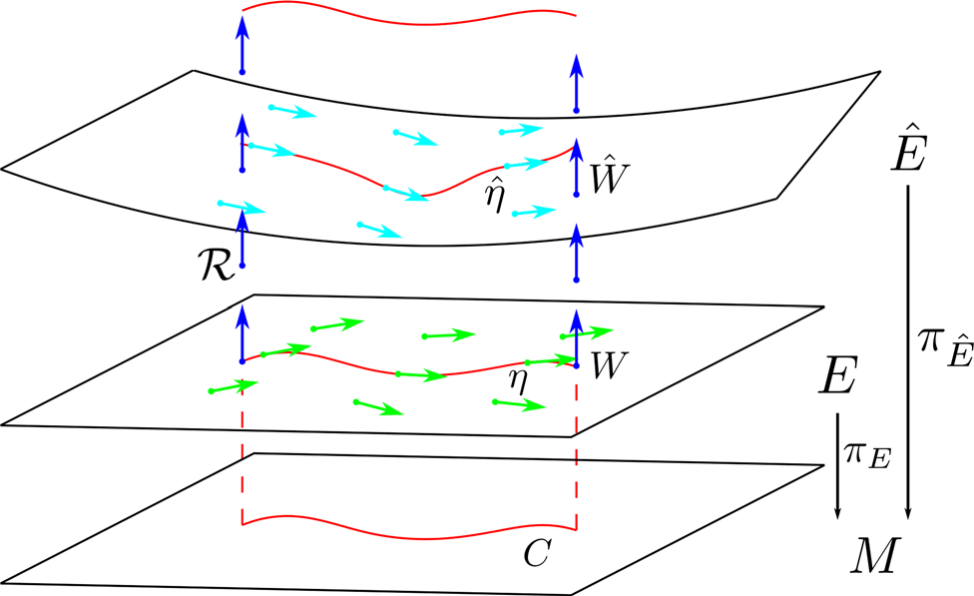
Given a two-dimensional manifold *M* and two different kinematic domains *E* and $\hat{E}$, we can observe the relationship between two projectively related Vlasov fields, *W* and $\hat{W}$ (see Theorem 2.18). The Vlasov field *W* (resp. $\hat{W}$), depicted by green (light blue) arrows, are tangent to *E* ($\hat{E}$) and generate integral curves $\eta$ ($\hat{\eta }$), depicted by red lines. Both integral curves project down into the same curve on *M*, denoted *C*, also a red line. The radial vector field $\mathcal {R}$ is denoted by dark blue arrows. This diagram can be identified with Fig. 3

The primary object of interest in this paper is the Vlasov bivector, $\Psi$. The Vlasov bivector is constructed on the conic bundle *U* as opposed to a given kinematic domain. Informally we may consider these to be the generalisation of a Vlasov field $W_E$ on *E* to a geometric object on *U*. Although we use the language of bivectors here, one can use the equivalent language of foliations to describe a Vlasov bivector. Each $\Psi$ is constructed in such a way that the two-dimensional leaves, see Figs. 1 and 5, that compose their foliations intersect any given kinematic domain *E* to produce one-dimensional curves, visualised in Fig. 6. These curves are exactly the integral curves of a Vlasov field associated with $\Psi$ which is tangent to *E*.

Vlasov bivectors also have the added benefit of accounting for all projectively related Vlasov fields simultaneously. Consequently, the Vlasov bivector accounts for all parametrisations in tandem without explicit reference to any of them. Furthermore, the equation for transforming Vlasov fields between kinematic domains can be easily derived from the Vlasov bivector, hence the choice to describe our formalism in terms of bivectors instead of foliations. Vlasov bivectors are the main topic of discussion in Sect. 3.

In general, it is not informative to attempt to extend the particle density function $f_E$ to *U*. Instead, we first replace $f_E$ with the particle density (2*n*–2)-form $\theta _E$. This is depicted in Fig. 4. This 6-form has two conditions, equivalent to Eq. (1), which together we call the *transport equations*. The first states that $\theta _E$ is closed, $d\theta _E=0$ which corresponds to the form manifolds not terminating (i.e. no particles being created or lost). The second states that the Vlasov field is tangent to $\theta _E$, i.e. $i_{W_E}\theta _E=0$. Furthermore, the transport equations apply to a more general set of theories than simply the Vlasov equation. The transport equations are discussed in Sect. 2.5.

The geometric notion of the transport equations can be more easily translated into our formalism. In our case, the particle density from on *E*, $\theta _E$, is replaced with the particle density form $\theta \in \Gamma \Lambda ^{2n-2}U$. This $(2n{-}2)$-form on a 2*n*-dimensional manifold is also depicted in Fig. 1, using the idea of form submanifolds described in [24]. The closure of $\theta$ corresponds to the fact that the form submanifolds do not have boundaries in *U*, and they are also tangent to all projectively related Vlasov fields *W*. In Sect. 4.1, the transport equations are generalised to *U* in terms of the Vlasov bivector and the particle density form on *U*.

Having established the Vlasov bivector, *W*, and the particle density form $\theta _E$, we show how to relate them to the corresponding Vlasov vector $W_E$ and density form $\theta _E$. Of course, this is only possible if we are dealing with a time-orientable system. We say that the conic bundle is time-orientable if we can write *U* in terms of a disjoint union of future pointing and past pointing vectors, discussed in Sect. 1.6. When *U* is time-orientable, we show how to translate between *W* and $W_E$, and between $\theta$ and $\theta _E$.

### Signposting

The remainder of Sect. 1 is devoted to the notation and conventions used throughout this paper. In Sect. 2, we discuss kinematic domains and the various objects built on them. We begin by introducing the kinematic domain and its properties in Sect. 2.1: a generalisation of the mass shell and lab-time bundles upon which we can perform kinematics. In this subsection, we also introduce the Vlasov field *W* on the conic bundle *U* and explore its relationship with the Vlasov field $W_E$ on kinematic domains. A useful tool for describing a kinematic domain is a kinematic indicator, and these are introduced and discussed in Sect. 2.2. Integral curves of Vlasov fields are discussed in Sect. 2.3. We also discuss the conditions for which two different Vlasov fields correspond to the same trajectories. In Sect. 2.4, we discuss how to transform Vlasov fields between kinematic domains in such a way that the trajectories of particles are preserved. In Sect. 2.5, we introduce the transport equations, a geometric method of interpreting the Vlasov equation on *E*. We also discuss some advantages of this approach. Next, Sect. 2.6 gives an example of how we may transform Vlasov fields between kinematic domains.

In Sect. 3, we introduce the Vlasov bivector $\Psi$. First, the necessary properties to define Vlasov bivectors are discussed in Sects. 3.1 and 3.2. Vlasov bivectors themselves, their benefits, and geometric interpretation are given in Sect. 3.3. We also illustrate the correspondence between bivectors and foliations here.

Section 4 deals with the particle density form on *U* and its applications. The transport equations are generalised to the conic bundle using the Vlasov bivector in Sect. 4.1. We also discuss the geometric interpretation of these new transport equations here. In Sect. 4.2, we explore the conditions necessary to define a particle density form on *U* given a particle density from on a kinematic domain *E* and vice versa. We then apply the particle density on *U* to define a current $(n{-}1)$-form on *M* in Sect. 4.3. We also show that this current form is the same as the current form typically defined on *E*. In Sect. 4.4, we define the stress–energy (*n*–1)-forms, where we see that, unlike the in case of the current, these depend on the choice of kinematic domain.

In Sect. 5, numerical applications for the framework are discussed. We begin in Sect. 5.1 with a discussion of the application of lab time in simulations, particularly in relativistic cases. We then discuss a particular integration scheme, the ANI and its generalisation in Sect. 5.2. This includes the introduction of an integration scheme which only uses one interpolation, and an application of our formalism: we perform a Lorentz boost on a Vlasov–Poisson system to write the equation for integrating the Vlasov field in a new frame. We conclude in Sect. 6.

In the appendices, we discuss the relationship between our work and sprays in Sect. A.1, some useful auxiliary lemmas are contained in Sect. A.2, and proofs which are not discussed in the body of the work are given in Sect. A.3.

### Notation and conventions for general manifolds

**Table 1:** Table of symbols use throughout

Object type	Set of objects	Generic	Specific
Manifolds	N.A	*N*, *P*, *K*	*M*, *E*, *U*
Scalar fields	$\Gamma \Lambda ^0N$	*h*, *k*	*f*, *F*, *g*
Vector fields	$\Gamma TN$	*X*, *Y*, *Z*, *V*	*W*
*p*-forms	$\Gamma \Lambda ^pN$	$\alpha ,\beta ,\gamma ,\omega$	$\theta ,\theta _E,\chi ,\mathcal {J},\mathcal {J}_E$
Bivectors	$\Gamma \mathcal {B}^2(N)$	$\Phi$	$\Psi$

Throughout this paper, Greek indices will run from 0 to $n-1$ ($\mu ,\nu =0,1,2,\ldots ,n-1$) and Latin indices will run from 1 to $n-1$ ($a,b=1,2,\ldots ,n-1$), unless specified otherwise. We will use *M* to denote a connected *n*-dimensional manifold and *TM* to denote the tangent bundle over *M*. All manifolds are assumed to be smooth. Points in *M* will generally be denote $p,q\in M$. Vectors on *M,* which are points in *TM* (and consequently the conic bundle *U*), will be denoted $\underline{u}\in U$. The space of vector fields over an arbitrary smooth manifold *N* of dimension $\ell$ is denoted $\Gamma TN$ and its elements are denoted $X,Y\in \Gamma TN$. Maps between abstract manifolds are denoted $\Theta :N\rightarrow P$. The space of scalar fields over *N* is denoted $\Gamma \Lambda ^0N$, and we denote its elements $f,g,h,F,G,H\in \Gamma \Lambda ^0N$ depending on context. The space of *r*-forms is given by $\Gamma \Lambda ^r N$, and we denote its elements by $\alpha ,\beta \in \Gamma \Lambda ^r N$. Although we consider both vectors at points and vector fields, we only consider *r*-form fields. Hence, when referring to *r*-forms, the word field is implicit. A field evaluated at a point will be denoted by $X\vert _{p}$ and the action of a vector field on a scalar field by $X\langle h\rangle$. A table of generic and specific symbols is given in Table 1.

The internal contraction of an *r*-form $\alpha$ by a vector field *X* is given by $i_X\alpha$ (or $\alpha {:} X$ for the special case when $\alpha$ is a 1-form), and the Lie derivative of $\alpha$ along *X* is denoted by $L_X\alpha$. Contraction by coordinate partial derivatives are denoted $i_\mu ^{(x)}=i_{\partial _\mu ^{(x)}}$ etc. A similar convention is used for Lie derivatives along a coordinate vector field where we denote $L_{\partial _\mu ^{(x)}}=L_\mu ^{(x)}$ etc.

#### Definition 1.1

*(Pullback and Pushforward)* Given a map between manifolds $\Theta :N\rightarrow P$ , then the *pushforward* of a vector at a point $\underline{v}\in T_p N$ is given by

3 $$\begin{aligned} \left( \Theta _*\underline{v} \right) \langle h \rangle = \underline{v}\langle h\circ \Theta \rangle . \end{aligned}$$

The *pullback* of a scalar field $h\in \Gamma \Lambda ^0P$ is given by

4 $$\begin{aligned} \Theta ^*h= (h\circ \Theta ). \end{aligned}$$

We may also pullback forms of arbitrary degree *r* according to the following rules:

5 $$\begin{aligned} \Theta ^*(d h)= d (\Theta ^*h) \quad \text {and}\quad \Theta ^*(\alpha \wedge \beta )=\Theta ^*\alpha \wedge \Theta ^*\beta , \end{aligned}$$

for $h\in \Gamma \Lambda ^0N,\; \alpha \in \Gamma \Lambda ^1 N,\; \beta \in \Gamma \Lambda ^{r-1}N$. The pullback and pushforward satisfy the compatibility property:

6 $$\begin{aligned} \alpha :\Theta _*\underline{v} = \Theta ^*\alpha :\underline{v}, \end{aligned}$$

for any 1-form $\alpha \in \Gamma \Lambda ^1P$ and point vector $\underline{v}\in T_pN$. Observe that we pushforward vectors at points, while we pull back *r*-forms.

#### Definition 1.2

*(Tangential Vector Fields)* Given an embedding $\Theta :K\hookrightarrow N$, a vector field $X\in \Gamma TN$ is *tangent* to the submanifold *K* if, there exists a vector field $Y\in \Gamma TK$ such that

7 $$\begin{aligned} \Theta _*(Y\vert _{p})= X\vert _{\Theta (p)}, \; \forall p\in K. \end{aligned}$$

Note that if *Y* exists then it is unique. In this case, we say that $Y\in \Gamma TK$ is induced by *X*. Given an *r*-form $\alpha \in \Gamma \Lambda ^r N$ then

8 $$\begin{aligned} \Theta ^*(i_X \alpha ) = i_{Y} (\Theta ^*\alpha ). \end{aligned}$$

#### Definition 1.3

*(Scalar Lift)* Given a scalar field $h\in \Gamma \Lambda ^0N$, the scalar lift defines a *scalar field* $\dot{h}\in \Gamma \Lambda ^0TN$ given by

9 $$\begin{aligned} \dot{h}\vert _{\underline{u}}= \underline{u} \langle h \rangle . \end{aligned}$$

#### Definition 1.4

*(Induced Coordinates)* Given a coordinate system $(x^0,\ldots ,x^{n-1})$ for the patch $K\subset N$, this induces a coordinate system $(\bar{x}^0,\ldots ,\bar{x}^{n-1},\dot{x}^0,\ldots ,\dot{x}^{n-1})$ for $\pi ^{-1}(K)\subset TN$, where $\bar{x}^\mu =\pi ^*x^\mu$ and $\dot{x}^\mu$ is the scalar lift. In this coordinate system an arbitrary vector field $X\in \Gamma TN$ can be written

10 $$\begin{aligned} \begin{aligned} X= \bar{X}^\mu \frac{\partial \;}{\partial \bar{x}^\mu }+ \hat{X}^\mu \frac{\partial \;}{\partial \dot{x}^\mu } \quad \text {where} \quad \bar{X}^\mu = X\langle {\bar{x}^\mu }\rangle \text { and } \hat{X}^\mu = X\langle {\dot{x}^\mu }\rangle . \end{aligned} \end{aligned}$$

Every vector field over *TN* can be defined using Eq. (10) and consequently can be defined entirely by its action on scalar fields of type $\pi ^*h$ and $\dot{h}$ for all $h\in \Gamma \Lambda ^0N$.

Throughout, we will use the shorthand

11 $$\begin{aligned} \partial _\mu ^{(x)}= \frac{\partial \;}{\partial x^\mu },\quad \partial _\mu ^{(\bar{x})}= \frac{\partial \;}{\partial \bar{x}^\mu },\quad \partial _\mu ^{(\dot{x})}= \frac{\partial \;}{\partial \dot{x}^\mu }. \end{aligned}$$

Note that $\partial _\mu ^{(x)}\in \Gamma TM$ while $\partial _\mu ^{(\bar{x})}\in \Gamma TTM$ and $\partial _\mu ^{(\dot{x})}\in \Gamma TTM$. In induced coordinates, the scalar lift of a scalar field $h\in \Gamma \Lambda ^0M$ is given by

12 $$\begin{aligned} \dot{h}= \dot{x}^\mu \partial ^{(x)}_\mu h. \end{aligned}$$

In general, we will not distinguish between $\bar{x}$ and *x* unless necessary.

When convenient, the exterior product of coordinate differential forms will be written

13 $$\begin{aligned} dx^0\wedge \cdots \wedge dx^{n-1} = dx^{0,\ldots ,n-1}. \end{aligned}$$

### Notation and conventions specific for this article

Let *M* be a spacetime manifold and $\pi :TM\rightarrow M$, the tangent bundle over *M*. The slit tangent bundle $\breve{T}M=TM\backslash \{0\}$ is the subset of *TM* which excludes the zero vectors.

#### Definition 1.5

*(Conic Sub-Bundle)* A *conic sub-bundle* is a subset of the slit tangent bundle $U\subset \breve{T}M$, which satisfies the following properties:

1. $$\pi (U)=M$$
2. If $\underline{u}\in U$ then for any $\lambda \ne 0$ we have $\lambda \underline{u}\in U$.

We also use $\pi$ to also denote the projection $\pi :U\rightarrow M$. A formal discussion of conic bundles can be found in [18]. We additionally assume the properties necessary for *U* to have a smooth sub-bundle structure.

#### Definition 1.6

*(Causal Indicator)* We say *U* is *time-orientable* if $U=U^+\cup U^-$ where $U^+\cap U^-=\varnothing$ and $\underline{u}\in U^+$ if and only if $-\underline{u}\in U^-$. We call $\underline{u}\in U^+$ *future pointing*. The *causal indicator* is the scalar field $\sigma \in \Gamma \Lambda ^0U$ given by

14 $$\begin{aligned} \sigma \vert _{\underline{u}}= {\left\{ \begin{array}{ll} 1, & \text{ if } \underline{u}\in U^+ \\ -1, & \text{ if } \underline{u}\in U^-. \end{array}\right. } \end{aligned}$$

Examples of structures described by conic bundles include the collection of time-like vectors over a spacetime manifold, the light cone, and the causal region of a time-orientable manifold. In the case where we consider only the time-like vectors, the corresponding conic bundle has an open sub-bundle structure. In the case where we consider the causally connected regions of spacetime (the former case plus the light cone), the corresponding conic bundle is a sub-bundle with a boundary. However, it is not a closed set since it excludes the zero vectors.

An important example of a conic bundle which is not time-orientable is $U=\breve{T}M$, for any manifold of dimension greater than 1. This is because each fibre, $\breve{T_p}M$, is connected.

As discussed in the introduction, when dealing with kinematic domains we restrict our attention to time-orientable conic bundles. By contrast, when dealing with the Vlasov bivector approach, we do not have to make this restriction. Thus, the bivector approach can be applied to the case when $U=\breve{T}M$.

#### Definition 1.7

*(Radial Vector Field)* The *radial vector field*, denoted by $\mathcal {R}\in \Gamma TU$, is the unique vector field that satisfies the following condition: for any $h\in \Gamma \Lambda ^0M$ and $\underline{u}\in U,$ we have

15 $$\begin{aligned} \mathcal {R}\langle \pi ^*h\rangle =0, \quad \text{ and } \quad \mathcal {R}\langle \dot{h} \rangle \vert _{\underline{u}}= \dot{h}\vert _{\underline{u}}= \underline{u}\langle h \rangle . \end{aligned}$$

In local induced coordinates, $\mathcal {R}$ is given by

16 $$\begin{aligned} \mathcal {R}= \dot{x}^\mu \partial ^{(\dot{x})}_\mu . \end{aligned}$$

This can be seen by acting $\mathcal {R}$ on the induced coordinates: $\mathcal {R}\langle \bar{x}^\mu \rangle =0$, $\mathcal {R}\langle \dot{x}^\mu \rangle = \dot{x}^\mu$. Within the literature, the radial vector field is known by many names, perhaps the most common of which is the vertical vector field. We have opted to refer to it as the radial vector field as it exhibits many useful radial properties.

#### Definition 1.8

*(Homogeneity)* A scalar field $G\in \Gamma \Lambda ^0U$ is said to be *homogeneous of degree* *k* (also known as radially homogeneous or fibre-wise homogeneous) if

17 $$\begin{aligned} G\vert _{\lambda {\underline{u}}}= \lambda ^k G\vert _{\underline{u}} \quad \text {for all}\quad \lambda \in \mathbb {R}\setminus \{ 0 \}. \end{aligned}$$

Note that by Euler’s theorem of homogeneous functions, a function $G\in \Gamma \Lambda ^0U$ is (fibre-wise) *k*-homogeneous if and only if it satisfies

18 $$\begin{aligned} \mathcal {R}\langle G\rangle =kG. \end{aligned}$$

## Vlasov systems and kinematic domains

### Kinematic domains and the Vlasov picture

In order to discuss the Vlasov field, we must first establish the space over which it is defined. We call such submanifolds kinematic domains. For this section, we assume that *U* is time-orientable in the sense of Definition 1.6, and that $E\subset U^+$, that is all points in *E* are future pointing. As stated in the introduction, this constraint is not needed for the generalisation to Vlasov bivectors. We assume that each spacetime manifold *M* (and hence *TM*) is connected.

#### Definition 2.1

*(Kinematic Domain)* A *Kinematic domain* is a $(2n{-}1)$-dimensional submanifold $E\subset U^+$ which satisfies the following properties:

1. $$\pi _E:E\rightarrow M$$ is surjective,
2. *E* is connected,
3. For any $\underline{u}\in U$, there exists a unique $\underline{v}\in E$ and $\lambda \ne 0$ such that $\underline{u}=\lambda \underline{v}$.

The inclusion map for *E* into *U* is given by $\Sigma _E:E \hookrightarrow U$.

For example, the unit mass shell, or as we refer to it throughout the remainder of this paper, the upper unit hyperboloid, given in Eq. (2), is a kinematic domain. We can write this as

19 $$\begin{aligned} E_{\text {H}}=\{ \underline{u}\in U: F_{\text {H}}=1 \text { and } \sigma |_{\underline{u}}=1 \}, \end{aligned}$$

where

20 $$\begin{aligned} F_{\text {H}}:U\rightarrow \mathbb {R};\; \underline{u}\mapsto -g_{\pi (\underline{u})}(\underline{u},\underline{u}). \end{aligned}$$

Another example of a kinematic domain is the lab-time bundle, with respect to a time-slicing scalar field *t*,

21 $$\begin{aligned} E_{t}=\{ \underline{v}\in U :\underline{v}\langle t \rangle =1\}. \end{aligned}$$

Recall that a time-slicing scalar $t\in \Gamma \Lambda ^0M$ requires that if $\sigma (\underline{v})=1$ then $\underline{v}\langle t\rangle > 0$.

An example from Finsler geometry^1^ is defined in [25], where the Finsler spacetime (*M*, *L*, *F*) admits an observer bundle

22 $$\begin{aligned} \mathcal {O}= \bigcup _{p\in M} \mathcal {S}_p, \end{aligned}$$

where

23 $$\begin{aligned} \mathcal {O}_p= \left\{ \begin{array}{c} \underline{u}\in T_pM \; :\; L\vert _{\underline{u}}=\pm 1, g^{(L)}_{\mu \nu }\vert _{\underline{u}} \text{ has } \text{ signature } (L,-L,-L,-L) \end{array} \right\} , \end{aligned}$$

and $\mathcal {S}_p$ is a non-empty closed connected component of $\mathcal {O}_p$. Here $g_{\mu \nu }^{(L)}$ is the metric induced by the fundamental function *L*.

We refer to two types of Vlasov fields throughout this paper: Vlasov fields on kinematic domains (denoted $W_E\in \Gamma TE$ for $E\subset U$) and Vlasov fields on *U* (denoted $W\in \Gamma TU$).

#### Definition 2.2

*(Vlasov field on E)* Given a kinematic domain *E*, a *Vlasov field on* *E*, $W_E\in \Gamma TE$, is a vector field that satisfies the horizontal condition

24 $$\begin{aligned} \pi _E{}_*(W_E\vert _{\underline{v}})=\underline{v}, \text{ or } \text{ equivalently, } W_E\langle \pi _E^*h \rangle =\dot{h}, \end{aligned}$$

for all $\underline{v}\in E$ and $h\in \Gamma \Lambda ^0E$. This equivalence is due to the fact $W_E\vert _{\underline{v}}\langle \pi _E^*h \rangle = \pi _E{}_*( W_E\vert _{\underline{v}} )\langle h \rangle = \underline{v}\langle h \rangle = \dot{h}\vert _{\underline{v}}$, for all $\underline{v}\in E$ and $h\in \Gamma \Lambda ^0 M$.

Given a particle density function $f_E\in \Gamma \Lambda ^0E$, the Vlasov field $W_E\in \Gamma TE$ satisfies Eq. (1). The flow of the Vlasov field defines a set of integral curves which trace out the paths of particles through phase space (see Sect. 2.3). The projections of these integral curves into the base space *M* are the trajectories of the particles described by $f_E$.

An example of the Vlasov field on $E_{\text {H}}$ is the Lorentz force Vlasov equation. In local coordinates $(x^\mu ,v^a)$ on $E_{\text {H}}$, with the embedding given by $\dot{x}^a=v^a$. Define $v^0$ by solving the constraint $g_{\mu \nu }v^\mu v^\nu =-1$ and choosing the root, such that $v^\mu \partial ^{(x)}_\mu \in U^+$ (note that here the $x^\mu$ in $\partial ^{(x)}_\mu$ is a coordinate on *M*). In this coordinate system, the Lorentz force Vlasov field is given by

25 $$\begin{aligned} W_{E_{\text {H}}}= v^\mu \partial _{\mu }^{(x)}- \left( \Gamma ^{a}_{\nu \rho }v^{\nu }v^{\rho } -\frac{q}{m}g^{a\nu }\mathcal {F}_{\nu \rho }v^\rho \right) \partial _{a}^{(v)}. \end{aligned}$$

Here $\mathcal {F}\in \Gamma \Lambda ^2M$ is the Faraday 2-form which satisfies the Maxwell equations. Dividing by $v^0$ gives the usual Vlasov field on $E_{\text {H}}$.

#### Definition 2.3

*(Vlasov Field on U)* Denoted $W\in \Gamma TU$, a *Vlasov field on* *U* is a vector field with the following defining properties:

1. *W* is horizontal,
  26 $$\begin{aligned} \pi _*(W\vert _{\underline{u}})=\underline{u}, \text{ or } \text{ equivalently, } W\langle \pi ^*h \rangle =\dot{h}, \end{aligned}$$
  for any point vector $\underline{u}\in U$ and $h\in \Gamma \Lambda ^0M$;
2. *W* is radially quadratic,
  27 $$\begin{aligned} W\langle \dot{h}\rangle \vert _{\lambda \underline{u}} = \lambda ^2 W\langle \dot{h}\rangle \vert _{\underline{u}}, \end{aligned}$$
  for any $h\in \Gamma \Lambda ^0M$, $\underline{u}\in U$, and $\lambda \in \mathbb {R}^+$.

From Eqs. (27) and (18), the radially quadratic property can equivalently be stated as

28 $$\begin{aligned} \mathcal {R}\langle W\langle \dot{h} \rangle \rangle =2W\langle \dot{h}\rangle . \end{aligned}$$

#### Lemma 2.4

Let $W\in \Gamma TU$ be horizontal. Then *W* is radially quadratic and hence a Vlasov field if and only if

29 $$\begin{aligned} [\mathcal {R},W]=W. \end{aligned}$$

#### Proof

Let *W* be radially quadratic then for any $f\in \Gamma \Lambda ^0M$, $W\langle \dot{f} \rangle$ is a 2-homogeneous scalar field. It follows that $\mathcal {R}\langle W\langle \dot{f}\rangle \rangle = 2W\langle \dot{f}\rangle$ by Eq. (28). Hence, $[\mathcal {R},W]\langle \dot{f}\rangle = \mathcal {R}\langle W\langle \dot{f}\rangle \rangle -W\langle \mathcal {R}\langle \dot{f}\rangle \rangle = W\langle \dot{f}\rangle$. From Eq. (26), we also have

$$\begin{aligned} {[}\mathcal {R},W]\langle \pi ^*f\rangle =\mathcal {R}\langle \dot{f} \rangle = \dot{f} =W\langle \pi ^*f\rangle , \end{aligned}$$

and hence, $$[\mathcal {R},W]=W$$.

Suppose that $[\mathcal {R},W]=W$, then for any $f\in \Gamma \Lambda ^0M$ we have

30 $$\begin{aligned} W\langle \dot{f} \rangle = [\mathcal {R},W]\langle \dot{f}\rangle = \mathcal {R}\langle W\langle \dot{f}\rangle \rangle - W\langle \dot{f}\rangle . \end{aligned}$$

Rearranging the above gives $\mathcal {R}\langle W\langle \dot{f}\rangle \rangle = 2W\langle \dot{f}\rangle$. Hence, *W* is radially quadratic. $\hfill\square$

Figure 3 shows two Vlasov fields on *U* that represent the same trajectories on *M*, just with different parametrisations. One is tangent to the unit hyperboloid $E_H$ while the other is tangent to a lab-time bundle $E_{t}$. In Sect. 2.4, we see the formula for transforming from one Vlasov field to another in such a way that the particle trajectories are unaffected.

The Vlasov field on *U* can be written in local coordinates as

31 $$\begin{aligned} W= \dot{x}^\mu \partial ^{(x)}_\mu +\varphi ^\mu \partial _\mu ^{(\dot{x})}, \end{aligned}$$

where $\varphi ^\mu$ are 2-homogeneous scalar fields, $\varphi ^\mu \vert _{\lambda \underline{u}}=\lambda ^2\varphi ^\mu \vert _{\underline{u}}$. The Vlasov field *W* on *U* can be reduced to a Vlasov field on *E* provided it is tangent to it.

#### Definition 2.5

*(Extension of a Vlasov Field on E)* Given a kinematic domain *E* with inclusion map $\Sigma _E:E\hookrightarrow U$ and a Vlasov field $W_E\in \Gamma TE$, we call $W\in \Gamma TU$ the extension of $W_E$ if *W* is tangent to *E* and $W_E$ is induced by *W*, as in Eq. (7),

32 $$\begin{aligned} \Sigma _{E}{}_*\left( W_{E}\vert _{\underline{v}} \right) = W \vert _{\Sigma _E(\underline{v})}, \end{aligned}$$

for all $$\underline{v}\in E$$.

#### Lemma 2.6

Let *W* be a Vlasov field and *G* be any *k*-homogeneous scalar field. Then for any $\lambda \ne 0$ and $\underline{u}\in U$,

$$\begin{aligned} W\vert _{\lambda \underline{u}}\langle G\rangle = \lambda ^{k+1} W\vert _{\underline{u}}\langle G\rangle . \end{aligned}$$

#### Proof

Let *G* be a *k*-homogeneous function. First note that

$$\begin{aligned} \partial _\mu ^{(\dot{x})}\left( G\vert _{\lambda \underline{u}} \right) = \lambda \left( \partial _\mu ^{(\dot{x})} G \right) \big \vert _{\lambda \underline{u}}, \end{aligned}$$

by the chain rule. Hence, we have

$$\begin{aligned} \begin{aligned} \left( \partial _\mu ^{(\dot{x})} G \right) \big \vert _{\lambda \underline{u}} = \lambda ^{-1} \partial _\mu ^{(\dot{x})}\left( G\vert _{\lambda \underline{u}} \right) = \lambda ^{-1} \partial ^{(\dot{x})}_\mu \left( \lambda ^k G\vert _{\underline{u}} \right) = \lambda ^{k-1} \left( \partial _\mu ^{(\dot{x})} G \right) \big \vert _{\underline{u}}. \end{aligned} \end{aligned}$$

We also have

$$\begin{aligned} \left( \partial _\mu ^{(x)}G \right) \big \vert _{\lambda \underline{u}}= \partial _\mu ^{(x)}\left( \lambda ^k G\vert _{\underline{u}} \right) =\lambda ^k \left( \partial _\mu ^{(x)}G \right) \big \vert _{\underline{u}}. \end{aligned}$$

By expanding the Vlasov field in coordinates, we get

$$\begin{aligned} \begin{aligned} W\vert _{\lambda \underline{u}}\langle G\rangle&= \left( \dot{x}^\mu \partial _\mu ^{(x)}G+ \varphi ^\mu \partial _\mu ^{(\dot{x})}G \right) \big \vert _{\lambda \underline{u}} = \lambda \underline{u} \lambda ^k \left( \partial _\mu ^{(x)}G \right) \big \vert _{\underline{u}} +\lambda ^2 \varphi ^\mu \vert _{\underline{u}} \lambda ^{k-1} \left( \partial _\mu ^{(\dot{x})} G \right) \big \vert _{\underline{u}} \\{}&= \lambda ^{k+1} \left( \dot{x}^\mu \partial _\mu ^{(x)}G+ \varphi ^\mu \partial _\mu ^{(\dot{x})}G \right) \big \vert _{\underline{u}} = \lambda ^{k+1} W\vert _{\underline{u}} \langle G\rangle , \end{aligned} \end{aligned}$$

hence the result. $\hfill\square$

It is worth noting at this point that what we call Vlasov fields on *U* are referred to as sprays and $W_E$ are referred to as semi-sprays in literature (see [26] or [27] for an overview). The correspondence between these objects is explored in Sect. A.1.

We now give the example of the Lorentz force equation and the corresponding Vlasov fields adapted to the unit hyperbolid $E_{\text {H}}$ and the lab bundle $E_{t}$. Here *U* is the conic bundle of time-like vectors. The Vlasov field on *U* adapted to $E_{\text {H}}$ is given by

33 $$\begin{aligned} W=\dot{x}^\mu \partial ^{(x)}_\mu + \left( \frac{q}{m} \sigma \sqrt{ F_{\text {H}}} g^{\mu \nu }\mathcal {F}_{\nu \rho }\dot{x}^\rho - \Gamma ^\mu _{\nu \rho }\dot{x}^\nu \dot{x}^\rho \right) \partial ^{(\dot{x})}_\mu , \end{aligned}$$

where $F_{\text {H}}$ is as defined in Eq. (20). The inclusion of the factor $\sigma \sqrt{F_{\text {H}}}$ is to ensure the $\partial ^{(\dot{x})}_\mu$ term is 2-homogeneous and future pointing. The parameterisation associated with the trajectories of this Vlasov field is proper time $\tau$. The Vlasov field on *U* describing particles subject to the Lorentz force in the lab-time bundle $W_{E_{t}}$ with associated lab time *t* is given by

34 $$\begin{aligned} \begin{aligned} \hspace{-0.6em} \hat{W}&= \dot{x}^\mu \partial ^{(x)}_\mu + \bigg ( \frac{q}{m} \sigma \sqrt{ F_{\text {H}}} g^{\mu \nu }\mathcal {F}_{\nu \rho }\dot{x}^\rho -\Gamma ^\mu _{\nu \rho }\dot{x}^\nu \dot{x}^\rho \bigg ) \partial ^{(\dot{x})}_\mu \\{}&\quad - \bigg ( \frac{q}{m} \sigma \sqrt{F_{\text {H}}} g^{\lambda \nu }\mathcal {F}_{\nu \rho }\dot{x}^\rho \frac{\partial t}{\partial x^\lambda } - \Gamma ^\lambda _{\nu \rho }\dot{x}^\nu \dot{x}^\rho \frac{\partial t}{\partial x^\lambda } +\dot{x}^\nu \dot{x}^\rho \frac{\partial t}{\partial x^\nu \partial x^\rho } \bigg ) \frac{\dot{x}^\mu }{\dot{t}} \partial _\mu ^{(\dot{x})}. \end{aligned} \end{aligned}$$

When using a lab-time bundle, it is often convenient to choose an adapted coordinate system $(t,x^1,x^2,x^3)$ on *M*. In this coordinate system, Eq. (34) becomes

35 $$\begin{aligned} \begin{aligned} \hat{W}&= \dot{x}^\mu \partial ^{(x)}_\mu + \bigg ( \frac{q}{m} \sigma \sqrt{ F_{\text {H}}} g^{\mu \nu }\mathcal {F}_{\nu \rho }\dot{x}^\rho -\Gamma ^\mu _{\nu \rho }\dot{x}^\nu \dot{x}^\rho \bigg ) \partial ^{(\dot{x})}_\mu \\ - \left( \frac{q}{m} \sigma \sqrt{F_{\text {H}}} g^{0\nu }\mathcal {F}_{\nu \rho }\dot{x}^\rho - \Gamma ^0_{\nu \rho }\dot{x}^\nu \dot{x}^\rho \right) \frac{\dot{x}^\mu }{\dot{t}}\partial _\mu ^{(\dot{x})}, \end{aligned} \end{aligned}$$

where $x^0=t$. We see in Sect. 2.6 that we can demonstrate the transformation between Eqs. (33) and (34) using the underlying second-order ODEs as done in Lemma 2.17. However, it is much easier to calculate these transformations after we have defined the kinematic indicator in the following subsection. This leads to the main result of this section in Theorem 2.18.

### Kinematic indicators

A kinematic domain can be defined in terms of a scalar field on *U*, and we call this field a kinematic indicator.

#### Definition 2.7

*(Kinematic Indicator)* A *kinematic indicator *for *E* is a non-vanishing scalar field $F\in \Gamma \Lambda ^0 U$ with nonzero integer degree of homogeneity *k*, $F\vert _{\lambda \underline{v}}=\lambda ^k F\vert _{\underline{v}}$, such that *F* is positively valued on $U^+$, and *E* is given by

36 $$\begin{aligned} E=\{ \underline{u}\in U :F\vert _{\underline{u}}=a \; \text{ and } \; \sigma \vert _{\underline{u}}=1\}, \end{aligned}$$

for some positive number *a*. Note that if *k* is odd, then the condition $\sigma \vert _{\underline{u}}=1$ becomes redundant.

For example, $F_{\text {H}}$ given in Eq. (20) is a kinematic indicator for $E_{\text {H}}$, the unit hyperboloid. A second example $F=\dot{t}$ is a kinematic indicator for $E_{t}$, the lab-time bundle.

#### Lemma 2.8

Given an arbitrary kinematic domain *E*, there exists a unique 1-homogeneous kinematic indicator $F\in \Gamma \Lambda ^0 U$ such that $E=\{ \underline{u}\in U: F\vert _{\underline{u}}=1 \}.$ For each $\underline{u}\in U,$ this is given by

37 $$\begin{aligned} F\vert _{\underline{u}}=\lambda \quad \text{ where } \;\underline{u}=\lambda \underline{v}\; \text{ for } \text{ a } \text{ unique } \underline{v}\in E. \end{aligned}$$

#### Proof

For each $\underline{u}\in U$, there exists a unique $\lambda \in \mathbb {R}\setminus \{0\}$ and $\underline{v}\in E$ such that $\underline{u}=\lambda \underline{v}$ by the conic properties of *U* (Definition 1.5). We define then $F\vert _{\underline{u}}=\lambda$. Since each component of *E* is connected, *F* is smooth, and hence, $F\in \Gamma \Lambda ^0 U$. To see that *F* is unique, suppose there is another scalar $F'$ which is 1-homogeneous and is such that such that $E=\{ \underline{u}\in U: F'\vert _{\underline{u}}=1 \}$ and pick any $\underline{u}\in U$. Then by the 1-homogeneity of $F'$, $F'\vert _{\underline{u}}=F'\vert _{\lambda \underline{v}}=\lambda =F\vert _{\underline{u}}$. $\hfill\square$

Given a kinematic indicator *F* for a kinematic domain *E* as defined in Lemma 2.8, we may define another scalar for some $k\in \mathbb {Z}$, $k\ne 0$ and $a\in \mathbb {R}^+$ by

38 $$\begin{aligned} \hat{F}= aF^k. \end{aligned}$$

Not only is $\hat{F}$ a *k*-homogeneous kinematic indicator for *E*, it is also unique for the chosen *k* and *a*.

#### Lemma 2.9

Let *F* be a kinematic indicator for *E* as given by Lemma 2.8 and let $\hat{F}$ be given by Eq. (38). $\hat{F}$ is a kinematic indicator for *E*.

#### Proof

Let $\hat{E}$ be a contour of $\hat{F}$ as given by Eq. (36). For and $\underline{v}\in E$, notice that $F^k\vert _{\underline{v}}=1$ so that $\hat{F}\vert _{\underline{v}}=aF^k\vert _{\underline{v}}=a$. Hence, $\underline{v}\in E$ implies $\underline{v}\in \hat{E}$. The converse can be proved similarly to show $\underline{v}\in \hat{E}$ implies $\underline{v}\in E$. Hence, $\hat{F}$ is a kinematic indicator for *E*. $\hfill\square$

#### Lemma 2.10

Let $a\in \mathbb {R}^+$ and $k\in \mathbb {N}$. If $\hat{F}$ is a *k*-homogeneous kinematic indicator for *E* such that Eq. (36) is satisfied, then $\hat{F}$ is uniquely given by Eq. (38).

#### Proof

$\hat{F}$ satisfies the following properties:

$$\begin{aligned} \hat{F}\vert _{\underline{v}}=a \quad \text {and}\quad \hat{F}\vert _{\lambda \underline{u}}= \lambda ^k \hat{F}\vert _{\underline{u}}, \end{aligned}$$

for all $\underline{v}\in E$, $\underline{u}\in U$ and $\lambda \ne 0$. These two functions can be related by

39 $$\begin{aligned} \hat{F}\vert _{\underline{v}}= a =a F\vert _{\underline{v}}, \quad \text { and } \quad \frac{(\sigma \hat{F})\vert _{\lambda \underline{u}}}{\hat{F}\vert _{\underline{u}}}= \sigma \vert _{\lambda \underline{u}}\lambda ^k=\sigma \vert _{\lambda \underline{u}}\left( \frac{F\vert _{\lambda \underline{u}}}{F\vert _{\underline{u}}} \right) ^k, \end{aligned}$$

for all $\underline{v}\in E,\;\underline{u}\in U$ and $\lambda \ne 0$.

By the conic property, for any $\underline{u}\in U$ there exists a unique $\underline{v}\in E$ and $\lambda \ne 0$ such that $\underline{u}=\lambda \underline{v}$. By plugging such a $\underline{v}$ into Eq. (39), we get

$$\begin{aligned} \frac{\hat{F}\vert _{\lambda \underline{v}}}{a}= \left( \frac{F\vert _{\lambda \underline{v}}}{1} \right) ^k \implies \hat{F}\vert _{\underline{u}}= aF^k\vert _{\underline{u}}. \end{aligned}$$

Hence, $$\hat{F}=aF^k$$. $$\hfill\square$$

In the case of the upper unit hyperboloid $F_{\text {H}}$ [Eq. (20)], the kinematic indicator is 2-homogeneous. The equivalent 1-homogeneous kinematic indicator is given by

40 $$\begin{aligned} \hat{F}_H\vert _{\underline{u}}= \sigma \vert _{\underline{u}} \sqrt{F_{\text {H}}\vert _{\underline{u}}}. \end{aligned}$$

where $\sigma$ is given by Definition 1.6.

At this point, we may observe some similarities with foliations of the tangent bundle based on Finsler metrics [28]. The function *F* which defines *E* can be a Finsler metric; however, it is not a necessary condition. The only requirement for *F* is that it be homogeneous of some degree.

#### Definition 2.11

*(Compatible Vlasov Field)* Given a kinematic indicator *F* with associated kinematic domain *E*, a Vlasov field $W\in \Gamma TU$ is said to be compatible with *F* if it satisfies $W\langle F\rangle =0$. The restriction of *W* to a compatible *E* is then denoted $W_E\in \Gamma TE$ as given by eq. (32).

Note that by Lemma A.5, *W* is tangent to a kinematic domain *E* with kinematic indicator *F* if and only if *W* is compatible with *F*.

#### Lemma 2.12

Given a kinematic domain *E* and a Vlasov field $W_E\in \Gamma TE$, there exists a unique Vlasov field $W\in \Gamma TU$ which is an extension of $W_E$, or equivalently $W_E$ is induced by *W*.

#### Proof

First observe that for any $f\in \Gamma \Lambda ^0M$ and $\underline{v}\in E$,

$$\begin{aligned} \left( \dot{f}\circ \Sigma _E \right) \vert _{\underline{v}}= \dot{f}\vert _{\Sigma _E(\underline{v})}=\dot{f}\vert _{\underline{v}}. \end{aligned}$$

It follows that $\dot{f}\circ \Sigma _E= \dot{f}$.

For all $\underline{u}\in U$ and $f\in \Gamma \Lambda ^0M$, define $W\in \Gamma TU$

$$\begin{aligned} \pi _*(W\vert _{\underline{u}}) = \underline{u} \; \text{ and } \; W\vert _{\underline{u}}\langle \dot{f}\rangle = \lambda ^2 W_E\vert _{\underline{v}}\langle \dot{f}\rangle , \end{aligned}$$

where $\underline{u}=\lambda \underline{v}$ by the conic property for $\underline{v}\in E$ and $\lambda \ne 0$. By construction, *W* is radially quadratic and horizontal so it remains to show that it is unique.

Let both *W* and $\hat{W}$ induce $W_E$ as defined above and set $X=\hat{W}-W$. Observe that $\pi _*X=0$ and

$$\begin{aligned} X\vert _{\underline{u}}\langle \dot{f}\rangle = \lambda ^2(W_E-W_E)\vert _{\underline{v}}\langle \dot{f}\rangle =0. \end{aligned}$$

Hence, it follows that $X=0$ and *W* is unique.

To see that *W* is compatible with *F* (and hence tangent to *E* by Lemma A.5), consider the following. Without loss of generality, we may assume *F* is 1-homogeneous. By Lemma 2.6, we have $W\vert _{\underline{u}}\langle F\rangle = \lambda ^2 W\vert _{\underline{v}}\langle F\rangle$. It follows that

$$\begin{aligned} \begin{aligned} W\vert _{\underline{u}}\langle F\rangle&= \lambda ^2 W\vert _{\underline{v}} \langle F\rangle = \lambda ^2 (\Sigma _E{}_*W_E\vert _{\underline{v}}) \langle F\rangle = \lambda ^2 W_E\vert _{\underline{v}} \langle F\circ \Sigma _E \rangle = \lambda ^2 W_E\vert _{\underline{v}} \langle 1 \rangle =0. \end{aligned} \end{aligned}$$

$$\hfill\square$$

### Trajectories, prolongations, and the horizontal condition on *U*

The notion of integral curves for Vlasov fields on kinematic domains can be extended to integral curves on *U*. The Vlasov field $W\in\Gamma TU$generates a set of integral curves on *U*, denoted $\eta$. The projections of these curves onto *M* are exactly the trajectories of the particles and are denoted *C*. Since we restrict our attention here to integral curves of horizontal vector fields, the terms prolongation and integral curve can be used interchangeably.

Trajectories can be considered in terms of maps from intervals of the real line $\mathcal {I}\subset \mathbb {R}$ into the spacetime manifold *M*:

41 $$\begin{aligned} C:\mathcal {I}\hookrightarrow M. \end{aligned}$$

These trajectories can be parameterised by choosing a parameter, $s\in \Gamma \Lambda ^0\mathcal {I}$ with $ds\ne 0$. We then write the parameterised trajectories as *C*(*s*).

#### Definition 2.13

*(Prolongations)* For some $s\in \Gamma \Lambda ^0\mathcal {I}$ and $\partial _s\in \Gamma T\mathcal {I}$, the *prolongation* of a curve $C:\mathcal {I}\hookrightarrow M$ is given by

42 $$\begin{aligned} \eta :\mathcal {I}\hookrightarrow U,\; \eta (s_0)=C_*(\partial _s\vert _{s_0}),\; \forall s_0\in \mathcal {I}. \end{aligned}$$

#### Lemma 2.14

$\eta :\mathcal {I}\hookrightarrow U$ is the prolongation of some trajectory *C* if and only if

43 $$\begin{aligned} (\pi \circ \eta )_*(\partial _s)=\eta . \end{aligned}$$

#### Proof

First, suppose $\eta$ is the prolongation of *C*. Then $C_*(\partial _s|_{s_0})\in T_{C(s_0)}M$. Hence, $\pi \big (\eta (s_0)\big ) = \pi \big (C_*(\partial _s|_{s_0})\big )=C(s)$, i.e. $\pi \circ \eta =C,$ and hence, $(\pi \circ \eta )_*(\partial _s)=C_*(\partial _s)=\eta$.

Conversely, assuming Eq. (43), let $C=\pi \circ \eta$, then $C_*(\partial _s) = (\pi \circ \eta )_*(\partial _s) = \eta$. $\hfill\square$

Let $\hat{\mathcal {I}}\subset \mathbb {R}$ be coordinated by $\hat{s}$ and let $\hat{C}:\hat{\mathcal {I}}\hookrightarrow M$ be an alternative parameterisation of *C*. That is, there exists a diffeomorphism $\hat{s}=\hat{s}(s)$ such that

44 $$\begin{aligned} \hat{C}\big (\hat{s}(s)\big )=C(s). \end{aligned}$$

Note that although the two parameterisations define the same curve, the prolongations $\eta$ and ${\hat{\eta }}$ do not coincide. Consequently, the tangent vectors along the prolongations (i.e. acceleration) belong to different spaces: $\eta _*(\partial _s\vert _{s_0}) \in T_{\eta (s_0)}U$ and $\hat{\eta }_*(\partial _{\hat{s}}\vert _{\hat{s}_0}) \in T_{\hat{\eta }(\hat{s}_0)}U$ even when $C(s_0)=\hat{C}(s_1)$.

#### Definition 2.15

An integral curve $\eta$ of *W* is said to lie along *E* if

45 $$\begin{aligned} \eta (s) \in E,\; \forall s\in \mathcal {I}. \end{aligned}$$

This is a necessary condition for $\eta$ to be an integral curve of $W_E$.

If $W_E$ is induced by *W*, $\eta$ is an integral curve of *W*, and $\eta$ lies along *E*, then $\eta$ is also an integral curve of $W_E$.

#### Lemma 2.16

All integral curves $\eta$ of a vector field $X\in \Gamma TU$ are prolongations if and only if *X* is horizontal.

#### Proof

Let $\underline{u}\in U$ and let $\eta$ be an integral curve of *X*, i.e. $X\vert _{\eta (s_1)}= \eta _*(\partial _s\vert _{s_1})$ for all $s_1\in \mathcal {I}$, such that $\eta (s_0)=\underline{u}$ for some $s_0\in \mathcal {I}$.

Suppose first that $\eta$ is a prolongation. Then

46 $$\begin{aligned} \begin{aligned} \pi _*X\vert _{\underline{u}}&= \pi _*X\vert _{\eta (s_0)} = \pi _*\eta _*(\partial _s\vert _{s_0}) = (\pi \circ \eta )_*(\partial _s\vert _{s_0})= \eta (s_0)= \underline{u}. \end{aligned} \end{aligned}$$

Hence, *X* is horizontal.

Suppose now that *X* is horizontal. We have $\pi _*X\vert _{\underline{u}}= \underline{u}= \eta (s_0)$. Then,

47 $$\begin{aligned} \eta (s_0)= \pi _*X\vert _{\eta (s_0)} = \pi _*\eta _*(\partial _s\vert _{s_0}) =(\pi \circ \eta )_*(\partial _s\vert _{s_0}). \end{aligned}$$

Hence, $\eta$ is a prolongation. $\hfill\square$

The integral curves $\eta$ of Vlasov fields $W\in \Gamma TU$ correspond to trajectories *C* in the base space *M* through the following relation:

48 $$\begin{aligned} C=\pi \circ \eta . \end{aligned}$$

In local coordinates $(x^\mu ,\dot{x}^\mu ),$ let $C^\mu (s_0)=x^\mu \vert _{C(s_0)}$ then

49 $$\begin{aligned} \dot{C}^\mu (s_0)= \frac{dC^\mu }{ds} \bigg \vert _{s_0} \; \text{ and } \; \ddot{C}^\mu (s_0)= \frac{d^2C^\mu }{ds^2}\bigg \vert _{s_0}. \end{aligned}$$

These trajectories can be expressed in terms of a parameterised system of second-order differential equations in terms of the coefficients of *W*:

50 $$\begin{aligned} \ddot{C}^\mu \vert _{s_0}=\varphi ^\mu \vert _{\dot{C}(s_0)}, \end{aligned}$$

where *s* is a parameter corresponding to *W* and $\varphi ^\mu$ are the coefficients of *W* as given in Eq. (31). Due to the projection in Eq. (48), there is a class of integral curves, with corresponding vector fields, that produce the same trajectories. These vector fields differ only by a term $k\mathcal {R}$ where $k\in \Gamma \Lambda ^0U$ is a 1-homogeneous function. The converse is also true, and a full proof of this statement can be found in [26]. These vector fields are said to be projectively related in the literature (see Definition A.2 for a definition). We present here a proof of only the former statement to illustrate this result in our mathematical notation.

#### Lemma 2.17

Let $W,\hat{W}\in \Gamma TU$ be Vlasov fields and let $k\in \Gamma \Lambda ^0U$ be a 1-homogeneous scalar field. If *W* and $\hat{W}$ are related by

51 $$\begin{aligned} \hat{W}=W+k\mathcal {R} \end{aligned}$$

,then they have the same trajectories up to a parameterisation. That is, if the trajectories *C* of *W* are parameterised by *s* then $\hat{W}$ has the same trajectories *C* only parameterised by $\hat{s}$ where

52 $$\begin{aligned} \frac{\text {d}^2\hat{s}}{\text {d}s^2}\bigg \vert _{s_0}+ k\vert _{\underline{u}} \frac{\text {d}\hat{s}}{\text {d}s}\bigg \vert _{s_0}=0,\; \frac{\text {d}\hat{s}}{\text {d}s}>0, \end{aligned}$$

and $$\underline{u}=C_*(\partial _s\vert _{s_0})\in U$$.

#### Proof

Notice that if *W* and $\hat{W}$ are related by Eq. (51) then in local coordinates we have

$$\begin{aligned} \hat{\varphi }^\mu =\varphi ^\mu + k\dot{x}^\mu , \end{aligned}$$

where $\varphi ^\mu$ (resp. $\hat{\varphi }^\mu$) are the $\partial _\mu ^{(\dot{x})}$ coefficients of *W* ($\hat{W}$), see eq. (31). Let the trajectories of *W* be denoted by *C*(*s*). These trajectories satisfy Eq. (50). Define a new parameter $\hat{s}=\hat{s}(s)$ by Eq. (52) and set $\hat{s}_0=\hat{s}(s_0)$.

The trajectory $C(\hat{s}(s))$ can be shown to satisfy

$$\begin{aligned} \frac{\text {d}^2C^\mu }{\text {d}s^2}\bigg \vert _{s_0}= \frac{\text {d}^2\hat{s}}{\text {d}s^2}\bigg \vert _{s_0}\frac{\text {d}C^\mu }{\text {d}\hat{s}}\bigg \vert _{\hat{s}_0} +\left( \frac{\text {d}\hat{s}}{\text {d}s}\bigg \vert _{s_0} \right) ^2 \frac{\text {d}^2C^\mu }{\text {d}\hat{s}^2}\bigg \vert _{\hat{s}_0}. \end{aligned}$$

We therefore have

$$\begin{aligned} \begin{aligned} \frac{\text {d}^2C^\mu }{\text {d}\hat{s}^2}&\bigg \vert _{\hat{s}_0}= \left( \frac{\text {d}\hat{s}}{\text {d}s}\bigg \vert _{s_0} \right) ^{-2} \! \left( \frac{\text {d}^2C^\mu }{\text {d}s^2}\bigg \vert _{s_0}- \frac{\text {d}^2\hat{s}}{\text {d}s^2}\bigg \vert _{s_0} \frac{\text {d}C^\mu }{\text {d}\hat{s}}\bigg \vert _{\hat{s}_0} \right) = \left( \frac{\text {d}\hat{s}}{\text {d}s}\bigg \vert _{s_0} \right) ^{-2} \bigg ( \varphi ^\mu \vert _{C_*(\partial _s\vert _{s_0})} +k\vert _{C_*(\partial _s\vert _{s_0})} \frac{\text {d}\hat{s}}{\text {d}s}\bigg \vert _{s_0} \frac{\text {d}C^\mu }{\text {d}\hat{s}}\bigg \vert _{\hat{s}_0} \bigg )\\ {}&= \varphi ^\mu \vert _{\underline{\hat{u}}}+ k\vert _{\underline{\hat{u}}} \frac{\text {d}dC^\mu }{\text {d}\hat{s}}\bigg \vert _{\hat{s}_0} = \left( \varphi ^\mu +k\dot{x}^\mu \right) \vert _{\underline{\hat{u}}} = \hat{\varphi }^\mu \vert _{\underline{\hat{u}}}, \end{aligned} \end{aligned}$$

where $\underline{\hat{u}}=C_*(\partial _{\hat{s}}\vert _{\hat{s}_0})$. The third line is due to the 2-homogeneity of $\varphi ^\mu$ and 1-homogeneity of *k*. Hence, $C(\hat{s})$ are the trajectories associated with $\hat{W}$. Hence, both *W* and $\hat{W}$ have the same trajectories up to a reparameterisation. $\hfill\square$

In the case of special relativity, with local coordinates $(t,x^a)$, where *t* is a lab-time coordinate and $s=t$, the Lorentz force equation may be written in velocity coordinates as

53 $$\begin{aligned} v^0=1,\quad \frac{\text {d}\vec {v}}{\text {d}t} = \frac{1}{\gamma } \frac{q}{m} \left( \vec {E} + \vec {v} \times \vec {B} \right) -\frac{1}{\gamma } \frac{q}{m} \left( \vec {v}\cdot \vec {E} \right) \vec {v}. \end{aligned}$$

where $\vec {v}$ is the 3-velocity of the particle and $\gamma$ is the Lorentz factor. We see that in this case the new (second) term in Eq. (51) correspond to the second term in Eq. (53), which arises from $\frac{d\gamma }{dt}$.

### Transforming between kinematic domains

Suppose we are given a Vlasov field $W_E$ on a kinematic domain *E* with kinematic indicator *F*. Using this data, we can construct a new Vlasov field $\hat{W}$ which is compatible with a new kinematic indicator $\hat{F}$ with associated kinematic domain $\hat{E}$. This defines a new Vlasov field $\hat{W}_{\hat{E}}$ on $\hat{E}$ which produces the same trajectories in the base space *M* as the initial Vlasov field $W_E$.

Once we have promoted $W_E$ into a Vlasov field *W* on *U* using Lemma 2.12, we can use it to construct a new Vlasov field $\hat{W}$ which is tangent to another kinematic domain $\hat{E}$. A visualisation of the following lemma is shown in Fig. 6.

#### Theorem 2.18

Let *E* and $\hat{E}$ be kinematic domains with kinematic indicator *F* and $\hat{F}$, respectively. Given a Vlasov field $W\in \Gamma TU$ which is compatible with *F*, we may construct a new Vlasov field $\hat{W}\in \Gamma TU$ given by

54 $$\begin{aligned} \hat{W}=W-\frac{W\langle \hat{F}\rangle }{\mathcal {R}\langle \hat{F}\rangle } \mathcal {R} \end{aligned}$$

which is compatible with $\hat{F}$ and corresponds to the same trajectories as *W* up to parameterisation.

#### Proof

It is clear that $\hat{W}\langle \hat{F}\rangle =0$. To see that the resultant vector field is horizontal, observe that

$$\begin{aligned} \begin{aligned} \pi _*(\hat{W}\vert _{\underline{u}})&= \pi _*(W\vert _{\underline{u}}) -\frac{W\langle \hat{F}\rangle }{\mathcal {R}\langle \hat{F}\rangle } \bigg \vert _{\underline{u}} \pi _*(\mathcal {R}\vert _{\underline{u}})= \pi _*(W\vert _{\underline{u}})= \underline{u} \end{aligned} \end{aligned}$$

To see that $\hat{W}$ is radially quadratic, observe that

$$\begin{aligned} \begin{aligned} \hat{W}\vert _{\lambda \underline{v}} \langle \dot{h}\rangle&= W\vert _{\lambda \underline{v}} \langle \dot{h}\rangle - \frac{W\langle \hat{F}\rangle }{\mathcal {R}\langle \hat{F}\rangle } \bigg \vert _{\lambda \underline{v}} \mathcal {R}\vert _{\lambda \underline{v}} \langle \dot{h}\rangle = \lambda ^2 W\vert _{\underline{v}} \langle \dot{h}\rangle - \frac{\lambda ^{k+1}W\vert _{\underline{v}}\langle \hat{F}\rangle }{\lambda ^k \mathcal {R}\vert _{\underline{v}} \langle \hat{F}\rangle } (\lambda \underline{v} \langle \dot{h}\rangle )\\&= \lambda ^2 \bigg ( W\vert _{\underline{v}} \langle \dot{h}\rangle - \frac{W \langle \hat{F}\rangle }{ \mathcal {R} \langle \hat{F}\rangle }\bigg \vert _{\underline{v}} \mathcal {R}\vert _{\underline{v}} \langle \dot{h}\rangle \bigg ) = \lambda ^2 \hat{W}\vert _{\underline{v}} \langle \dot{h} \rangle , \end{aligned} \end{aligned}$$

for any $h\in \Gamma \Lambda ^0 M$, $\underline{v}\in U$, $\lambda \ne 0$. Hence, $\hat{W}$ is a valid Vlasov field. To see that $\hat{W}$ corresponds to the same set of trajectories as *W*, notice that $W\langle \hat{F}\rangle / \mathcal {R}\langle \hat{F}\rangle$ in Eq. (54) is a 1-homogeneous function. Hence, by 2.17, *W* and $\hat{W}$ have the same trajectories up to parameterisation. $\hfill\square$

### The transport equations on kinematic domains

A method of interpreting the Vlasov equation in a kinematic domain *E* can be given in terms of the transport equations [29]. The transport equations are written in terms of a particle density form which codifies the phase space trajectories corresponding to a given Vlasov field. With some additional structure (a choice of non-vanishing top form, otherwise known as a measure), we can recover the usual Vlasov equation (i.e. a particle density function $f_E\in \Gamma \Lambda ^0 E$ such that $W_E\langle f_E \rangle =0$) from the transport equations on our preferred choice of kinetic domain *E*.

#### Definition 2.19

*(Transport Equations on E)* Consider a kinematic domain $E\subset U$, a $(2n{-}2)$-form $\theta _E\in \Gamma \Lambda ^{2n-2}E$, and a Vlasov field $W_E\in \Gamma TE$. The *transport equations on* *E* are written

55 $$\begin{aligned} d\theta _E=0,\; i_{W_E}\theta _E=0. \end{aligned}$$

A visualisation of the transport equations is given in Fig. 4. These geometric statements are equivalent to the Vlasov equation under appropriate conditions. We call $\theta _E$ a particle density form on *E*.

Let *E* be a kinematic domain, $W_E\in \Gamma TE$ a Vlasov field on *E*, and let $\Omega _E\in \Gamma \Lambda ^{2n-1}E$ be a measure on *E* such that

56 $$\begin{aligned} L_{W_E}\Omega _E=0. \end{aligned}$$

We can relate a particle density $(2n{-}2)$-form $\theta _E\in \Gamma \Lambda ^{2n-2}E$ and a particle density function (PDF) $f_E\in \Gamma \Lambda ^0E$ via

57 $$\begin{aligned} \theta _E= f_Ei_{W_E} \Omega _E. \end{aligned}$$

#### Lemma 2.20

Given $\theta _E\in \Gamma \Lambda ^{2n-2}E$ such that $i_{W_E}\theta _E=0$ then $f_E\in \Gamma \Lambda ^0E$, given by Eq. (57), exists and is unique. Furthermore, $\theta _E$ satisfies the transport equations Eq. (55) if and only if $f_E$ satisfies Eq. (1) that is $W_E\langle f_E\rangle =0$.

#### Proof

Let $(x^0,\ldots ,x^{2n-2})$ define a coordinate system on *E* such that $\partial _0^{(x)}=W_E$. Then,

$$\begin{aligned} \Omega _E=\Omega _0\text {d}x^0\wedge \cdots \wedge \text {d}x^{2n-2}, \text{ and } \theta _E= \theta _E^\mu i_\mu ^{(x)}\Omega _E, \end{aligned}$$

where $\mu =0,\ldots ,2n-2$. We then have

$$\begin{aligned} \begin{aligned} 0 = i_{W_E}\theta _E&= i_0^{(x)}\theta _E = \theta _E^0 i_0^{(x)}i_0^{(x)} \Omega _E + \theta _E^a i_0^{(x)} i_a^{(x)} \Omega _E = \theta _E^a i_0^{(x)} i_a^{(x)} \Omega _E, \end{aligned} \end{aligned}$$

where $a=1,\ldots ,2n-2$. Hence, $\theta _E^a=0$ for each *a*. It follows that $\theta _E= \theta _E^0i_0^{(x)}\Omega$, i.e. $\theta _E=f_E i_{W_E}\Omega _E$.

From Eq. (57), we have

$$\begin{aligned} \begin{aligned} \text {d}\theta _E&= \text {d}f_E\wedge i_{W_E}\Omega _E= i_{W_E}df \wedge \Omega _E = W_E\langle f_E\rangle \Omega _E; \end{aligned} \end{aligned}$$

hence, the transport equations for $\theta _E$ are equivalent to Eq. (1). $\hfill\square$

As a corollary of Lemma 2.20, the transport equations and the Vlasov equation, Eq. (1), can equivalently be cast as

58 $$\begin{aligned} L_{W_E}(f_E\Omega _E) = 0. \end{aligned}$$

An example of the transport equations can be seen in [25]. However, these equations are subtly different in that a form $\omega _E=i_{W_E}\Omega _E$ with $L_{W_E}\Omega _E =0$ is defined and the transport equations are cast as $d\omega _E=0$ and $i_{W_E}\omega _E=0$. Note that the particle density function $f_E$ is absent from this definition.

Given a volume form $\Omega \in \Gamma \Lambda ^{2n} U$ on *U*, such that $L_W\Omega =0$, and kinematic domain $E\subset U$ with inclusion map $\Sigma _E: E\hookrightarrow U$, the typical choice of top form on *E* is given by

59 $$\begin{aligned} \Omega _E= \Sigma _E^*(i_{\mathcal {R}}\Omega ). \end{aligned}$$

#### Lemma 2.21

Let $\Omega \in \Gamma \Lambda ^{2n}U$ such that $L_W\Omega =0$. Let $\Omega _E\in \Gamma \Lambda ^{2n-1}E$ be defined by Eq. (59), then Eq. (56) holds.

#### Proof

Observe that

$$\begin{aligned} \begin{aligned} L_{W_E}\Omega _E&= L_{W_E} \left( \Sigma _E^*i_{\mathcal {R}}\Omega \right) = \Sigma _E^*\left( L_Wi_{\mathcal {R}}\Omega \right) = \Sigma _E^*\left( i_{[W,\mathcal {R}]}\Omega + i_{\mathcal {R}} L_W\Omega \right) = -\Sigma _E^*i_W\Omega = -i_{W_E} \Sigma _E^*\Omega = 0, \end{aligned} \end{aligned}$$

since the degree of $\Omega$ is greater than the dimension of *E*. $\hfill\square$

In the instance that the chosen kinematic domain is the upper unit hyperboloid $E_{\text {H}}$ as given in Eq. (19), then the volume form typically chosen is

60 $$\begin{aligned} \Omega _{E_{\text {H}}}= -\frac{\det (g)}{\dot{x}_0} \text {d}x^0\wedge \cdots \wedge \text {d}x^{n-1}\wedge \text {d}\dot{x}^1\wedge \cdots \wedge \text {d}\dot{x}^{n-1}. \end{aligned}$$

Furthermore, if $W_{E_{\text {H}}}$ is the Vlasov field corresponding to the Lorentz force it can be shown that $L_{W_{E_{\text {H}}}}\Omega _{E_{\text {H}}}=0,$ and hence, the transport equations equipped with $\Omega _{E_{\text {H}}}$ are equivalent to the standard Vlasov equation. The choice of volume form in (59) which produces (60) is given by

61 $$\begin{aligned} \Omega = -\det (g)\, \text {d}x^0\wedge \cdots \wedge \text {d}x^{n-1}\wedge \text {d}\dot{x}^1\wedge \cdots \wedge \text {d}\dot{x}^{n}. \end{aligned}$$

It is a straightforward exercise to show that $L_W\Omega =0$ for the above $\Omega$ and *W* given by Eq. (33).

It is often the case that we know the measure only on the time slices of *E*, in Sect. 5.2, for example. In the following lemma, we show that this is sufficient information to specify $\Omega$ uniquely. We consider the scalar field *s* on *E* to be the time slicing, and $\alpha$ to be the measure on each time slice. For example, if the particle current density at time *s* is of the form $\int \dot{x}^\mu \, f_E\, \omega _0\, \text {d}\dot{x}^{1,\cdots ,n-1}$, where we integrate over the velocities, then we set $\alpha =\omega _0\, \text {d}\dot{x}^{1,\ldots ,n-1}$. The other data required is the constant $\lambda \in \mathbb {R}$ which enables us to extend the uniqueness to the entirety of *U*. Of course, $\Omega$ will depend on the choice of $\alpha$ and $\lambda$.

#### Lemma 2.22

Given an $\alpha \in \Gamma \Lambda ^{2n-2} E$ and $\lambda \in \mathbb {R}$. Let $s\in \Gamma \Lambda ^0 E$, such that $ds\ne 0$ on *E*, let $S_{s_0}=\{\underline{v}\in E, s|_{\underline{v}}=s_0\}\subset E$ and define $\Xi _{s_0} :S_{s_0} \rightarrow E$. If there exists an $\Omega \in \Gamma \Lambda ^{2n} U$ which satisfies $L_W\Omega =0$, $L_{\mathcal {R}}\Omega =\lambda \Omega$, and $\Xi _{s_0}^*i_{W_E} \Sigma ^*_E i_{\mathcal {R}}\Omega =\Xi _{s_0}^*\alpha$ for each $s_0$, then that $\Omega$ is unique.

#### Proof

Suppose there exists $\Omega ,\hat{\Omega }\in \Gamma \Lambda ^{2n} U$ satisfying the relevant conditions and define $\omega =\Omega -\hat{\Omega }$. Define a coordinate system on *U* such that $\mathcal {R}=\partial ^{(x)}_0,\; W=\partial ^{(x)}_1.$ Let $\omega =\omega '(x^0,\ldots ,x^{2n-1}) dx^0\wedge \ldots \wedge dx^{2n-1}$. Then $L_W\Omega =0$ and $L_W\hat{\Omega }=0$ implies $L_W\omega =0$. Furthermore,

$$\begin{aligned} \begin{aligned} \lambda \omega&= L_{\mathcal {R}}\omega = (\partial ^{(x)}_{0}\omega ') \text {d}x^0\wedge \cdots \wedge \text {d}x^{2n-1} + \omega ' L^{(x)}_{0} \text {d}x^0\wedge \cdots \wedge \text {d}x^{2n-1} = (\partial ^{(x)}_{0}\omega ') \text {d}x^0\wedge \cdots \wedge \text {d}x^{2n-1}; \end{aligned} \end{aligned}$$

hence,

$$\begin{aligned} \omega =\omega '(x^2,\ldots ,x^{n-1}) e^{\lambda x^0} \text {d}x^0\wedge \cdots \wedge \text {d}x^{2n-1}. \end{aligned}$$

Also, since $\Xi _{s_0}^*i_{W_E} \Sigma ^*_E i_{\mathcal {R}}\Omega =\alpha$ (similarly for $\hat{\Omega }$) for each $s_0$, we have $\Xi _{s_0}^*i_{W_E} \Sigma _E^*i_{\mathcal {R}}\omega =0$; thus, $\Omega '_0(x^2|_{s_0},\ldots ,x^{n-1}|_{s_0})=0$ for all $s_0$ so $\omega '=0,$ and hence, $\omega =0$. $\hfill\square$

We observe that $\Omega$ given by (61) satisfies $L_{\mathcal {R}}\Omega =n \Omega$. Therefore, by setting $s=x^0$ and $\alpha =(-\dot{x}^0 \det g / \dot{x}_0) \text {d}x^{1,\cdots ,n-1} \wedge \text {d}\dot{x}^{1,\cdots ,n-1}$, Lemma 2.22 tells us that Eq. (61) is unique.

There are instances when it is advantageous to cast the Vlasov equation in terms of the transport equations even when the flow of the measure is not preserved by the Vlasov field (i.e. $L_{W_E}\Omega _E\ne 0$). In [30], an attempt is made to account for the radiation reaction within the Vlasov field over a four-dimensional spacetime manifold *M*. To accomplish this, the Vlasov equation is cast as

62 $$\begin{aligned} L_{W_Q}(f_Q\omega _Q)=0. \end{aligned}$$

Here the 10-dimensional manifold *Q* is a subset of the double copy of the tangent bundle $Q\subset TM \oplus TM$ where the first copy of the tangent bundle contains normalised time-like velocity vectors (as on the upper unit hyperboloid), and the second copy contains acceleration vectors which are orthogonal to these velocities. The measure $\omega _Q\in \Gamma \Lambda ^{10}Q$ is formed on *Q* by pulling back a specific 10 form related to the measure on $TM \oplus TM$. In this case, the flow of $\omega _Q$ along $W_Q$ is not conserved:

63 $$\begin{aligned} L_{W_Q}\omega _Q= \frac{3}{\tau } \omega _Q, \text{ or } \text{ equivalently } W_Q \langle f_Q\rangle + \frac{3}{\tau }f_Q=0, \end{aligned}$$

where $\tau =q^2/6\pi m$. The latter equation is similar to Eq. (1), but the additional term on the LHS is to account for losses due to radiation. Note that, although the Vlasov equation is altered, the transport equations remain the same:

64 $$\begin{aligned} \text {d} \theta _Q=0, \quad i_{W_Q}\theta _Q=0, \end{aligned}$$

where

65 $$\begin{aligned} \theta _Q= i_{W_Q}(f_Q\omega _Q). \end{aligned}$$

### Example: the unit hyperboloid and lab-time Vlasov vector fields for the Lorentz force equation

In Eqs. (33), (34), and (35), we gave the Vlasov vector field adapted to the upper unit hyperboloid, an arbitrary time slicing, and a time slicing with respect to coordinate time. We stated that one method to find the transformation is to calculate second-order ODEs, perform the reparameterisation, and then recalculate the corresponding Vlasov vector field, which we do here.

#### Lemma 2.23

Equation (33) can be transformed into Eq. (34), by reparameterising the Lorentz force ODE.

#### Proof

Let *t* be the parameterisation of the trajectories *C* of the Vlasov field $W_{E_{\text {H}}}$ (i.e. Eq. (33). The Lorentz force ODE is given in coordinates by

$$\begin{aligned} \frac{\text {d} ^2 C^\mu }{\text {d} \tau ^2} = \frac{q}{m}\sigma \sqrt{F_{\text {H}}} g^{\mu \nu }\mathcal {F}_{\nu \rho } \frac{\text {d} C^\rho }{\text {d} \tau } - \Gamma ^\mu _{\nu \rho } \frac{\text {d} C^\nu }{\text {d} \tau } \frac{\text {d} C^\rho }{\text {d} \tau }. \end{aligned}$$

Let $W_{E_{t}}$, given by Eq. (34), be parameterised by *t*. Assuming the existence of a relationship $t=t(\tau ),$ the above ODE can be reparameterised as

66 $$\begin{aligned} \begin{aligned} \frac{\text {d} ^2 C^\mu }{\text {d} t^2} =&\frac{q}{m}\sigma \sqrt{F_{\text {H}}} g^{\mu \nu }\mathcal {F}_{\nu \rho } \frac{\text {d} C^\rho }{\text {d} t} - \Gamma ^\mu _{\nu \rho } \frac{\text {d} C^\nu }{\text {d} t} \frac{\text {d} C^\rho }{\text {d} t} - \left( \frac{\text {d}t}{\text {d}\tau } \right) ^{-3} \frac{\text {d}^2t}{\text {d}\tau ^2} \frac{\text {d}C^\mu }{\text {d}t}. \end{aligned} \end{aligned}$$

Let the integral curves of $W_{E_{\text {H}}}$ and $W_{E_{t}}$ be given by $\eta _{E_{\text {H}}}$ and $\eta _{E_{t}}$, respectively. Let $t_0=t(\tau _0),$ then we have the following relations:

$$\begin{aligned} \frac{\text {d}t}{\text {d}\tau } \bigg \vert _{\tau _0}=&\dot{t}\vert _{\eta _{E_{\text {H}}}(\tau _0)}, \end{aligned}$$

$$\begin{aligned} \begin{aligned} \frac{\text {d}^2t}{\text {d}\tau ^2}&\bigg \vert _{\tau _0} = \eta _{E_{\text {H}}}{}_*(\partial _\tau \vert _{\tau _0}) \langle \dot{t}\rangle = W_{E_{\text {H}}}\vert _{\eta _{E_{\text {H}}}(\tau _0)} \langle \dot{t}\rangle = W_{E_{\text {H}}}\vert _{\eta _{E_{\text {H}}}(\tau _0)}\left\langle \dot{x}^\nu \frac{\partial t}{\partial x^\nu } \right\rangle \\ =&\bigg ( \frac{q}{m} \sigma \sqrt{F_{\text {H}}} g^{\lambda \nu }\mathcal {F}_{\nu \rho }\dot{x}^\rho \frac{\partial t}{\partial x^\lambda } - \Gamma ^\lambda _{\nu \rho }\dot{x}^\nu \dot{x}^\rho \frac{\partial t}{\partial x^\lambda } +\dot{x}^\nu \dot{x}^\rho \frac{\partial t}{\partial x^\nu \partial x^\rho } \bigg ) \bigg \vert _{\eta _{E_{\text {H}}}(\tau _0)}\\ =&\frac{q}{m} \left( \sigma \sqrt{F_{\text {H}}} g^{\lambda \nu }\mathcal {F}_{\nu \rho } \right) \big \vert _{\eta _{E_{\text {H}}}(\tau _0)} \frac{\text {d} C^\rho }{\text {d}\tau }\bigg \vert _{\tau _0} \frac{\partial t}{\partial x^\lambda }\bigg \vert _{\tau _0} - \Gamma ^\lambda _{\nu \rho } \big \vert _{\eta _{E_{\text {H}}}(\tau _0)} \frac{\text {d} C^\nu }{\text {d}\tau }\bigg \vert _{\tau _0} \frac{d C^\rho }{\text {d}\tau }\bigg \vert _{\tau _0} \frac{\partial t}{\partial x^\lambda } \bigg \vert _{\tau _0} +\frac{\text {d} C^\nu }{\text {d}\tau }\bigg \vert _{\tau _0} \frac{\text {d} C^\rho }{\text {d}\tau }\bigg \vert _{\tau _0} \frac{\partial t}{\partial x^\nu \partial x^\rho }\bigg \vert _{\tau _0}\\ =&\left( \frac{\text {d}t}{\text {d}\tau }\bigg \vert _{\tau _0} \right) ^2 \bigg ( \frac{q}{m} \left( \sigma \sqrt{F_{\text {H}}} g^{\lambda \nu }\mathcal {F}_{\nu \rho } \right) \big \vert _{\eta _{E_{t}}(t_0)} \frac{\text {d} C^\rho }{\text {d}t}\bigg \vert _{t_0} \frac{\partial t}{\partial x^\lambda }\bigg \vert _{t_0} - \Gamma ^\lambda _{\nu \rho } \big \vert _{\eta _{E_{t}}(t_0)} \frac{\text {d} C^\nu }{\text {d}t}\bigg \vert _{t_0} \frac{\text {d} C^\rho }{\text {d}t}\bigg \vert _{t_0} \frac{\partial t}{\partial x^\lambda } \bigg \vert _{t_0}\\ {}&\quad +\frac{d C^\nu }{\text {d}t}\bigg \vert _{t_0} \frac{\text {d} C^\rho }{\text {d}t}\bigg \vert _{t_0} \frac{\partial t}{\partial x^\nu \partial x^\rho }\bigg \vert _{t_0} \bigg ) \end{aligned} \end{aligned}$$

Inserting the above relationships into Eq. (66), we get

67 $$\begin{aligned} \begin{aligned} \frac{\text {d}^2 C^\mu }{\text {d} t^2} =&\frac{q}{m}\sigma \sqrt{F_{\text {H}}} g^{\mu \nu }\mathcal {F}_{\nu \rho } \frac{\text {d}C^\rho }{\text {d} t} - \Gamma ^\mu _{\nu \rho } \frac{\text {d}C^\nu }{\text {d} t} \frac{\text {d}C^\rho }{\text {d} t}\\ {}&-\left( \dot{t}\right) ^{-1} \bigg ( \frac{q}{m} \sigma \sqrt{F_{\text {H}}} g^{\lambda \nu }\mathcal {F}_{\nu \rho } \frac{\text {d} C^\rho }{\text {d}t} \frac{\partial t}{\partial x^\lambda } - \Gamma ^\lambda _{\nu \rho } \frac{\text {d} C^\nu }{\text {d}t} \frac{\text {d} C^\rho }{\text {d}t} \frac{\partial t}{\partial x^\lambda } +\frac{\text {d}C^\nu }{\text {d}t} \frac{\text {d}C^\rho }{\text {d}t} \frac{\partial t}{\partial x^\nu \partial x^\rho } \bigg ) \frac{\text {d}C^\mu }{\text {d}t}. \end{aligned} \end{aligned}$$

We may identify this with the $\partial ^{(\dot{x})}_\mu$ term in Eq. (34) using Eq. (50). Hence, $W_{E_{t}}$ corresponds to the above reparameterised Lorentz force equation. If we were to choose an adapted coordinate system $(x^0=t,x^a,\dot{x}^\mu ),$ then we would have

68 $$\begin{aligned} \frac{\partial t}{\partial x^\mu }=\delta ^0_\mu , \quad \frac{\partial t}{\partial x^\mu \partial x^\nu }=0. \end{aligned}$$

Plugging these into Eq. (67) gives the $\partial ^{(\dot{x})}_\mu$ coefficient of Eq. (35). $\hfill\square$

Alternatively, we can use the transformation formula Eq. (54).

#### Lemma 2.24

Equation (33) can be transformed into Eq. (34) and hence Eq. (35), using the transformation formula Eq. (54).

#### Proof

Follows from

69 $$\begin{aligned} \begin{aligned} \frac{W_{E_{\text {H}}}\left\langle \dot{t} \right\rangle }{\mathcal {R}\left\langle \dot{t} \right\rangle }\mathcal {R}^\mu =&\frac{q}{m} \sqrt{F_{\text {H}}} g^{\lambda \nu }\mathcal {F}_{\nu \rho }\dot{x}^\rho \frac{\partial t}{\partial x^\lambda } \frac{\dot{x}^\mu }{\dot{t}} - \Gamma ^\lambda _{\nu \rho }\dot{x}^\nu \dot{x}^\rho \frac{\partial t}{\partial x^\lambda } \frac{\dot{x}^\mu }{\dot{t}} +\dot{x}^\nu \dot{x}^\rho \frac{\partial t}{\partial x^\nu \partial x^\rho } \frac{\dot{x}^\mu }{\dot{t}}. \end{aligned} \end{aligned}$$

$$\hfill\square$$

Thus, we see that the use of the transformation formula greatly simplifies the task of readapting the Vlasov vector field from one kinematic domain to another. Furthermore, we will see below that Eq. (54) can be trivially derived from the Vlasov bivector. This is shown in Lemma 3.21.

## The Vlasov bivectors

Here we present the Vlasov Bivector, the key goal of this article. The advantages of this approach have been listed in Sect. 1.2. The Vlasov bivector $\Psi$ can always be expressed as

70 $$\begin{aligned} \Psi = \mathcal {R} \wedge W, \end{aligned}$$

for any appropriate $W\in \Gamma TU$. It is trivial to see that any Vlasov fields related by Eqs. (51) and (54) produce the same Vlasov bivector, and so represents an entire class of equivalent Vlasov fields. Throughout this section, $\Psi$ will be reserved for Vlasov bivectors while an arbitrary bivector will be denoted $\Phi$.

### Bivectors

First, we write the definition of bivectors and explore some of their important properties.

#### Definition 3.1

*(Bivector)* A *bivector* over a manifold *N* is an exterior product of vector fields $X_\mu ,Y_\mu \in \Gamma \ TN$ over *N*, denoted by $\sum _\mu X_\mu \wedge Y_\mu$, for an arbitrary summation index $\mu$. These follow the standard rules for exterior products, namely ‘f’-linearity and antisymmetry.

#### Definition 3.2

*(Simple Bivectors)* A bivector over *N*, $\Phi$ is said to be *simple* if there exists $X,Y\in \Gamma TN$ such that $\Phi =X\wedge Y$. The space of all simple bivectors (fields) over *N* is denoted by $\Gamma \mathcal {B}^2(N)$.

We require that a Vlasov bivector be simple and take the form Eq. (70). In order to formally write the definition of $\Psi ,$ we first observe the following property of simple bivectors.

#### Definition 3.3

*(Bivector Pairing)* A bivector $\sum _\mu X_\mu \wedge Y_\mu$ acts on a pair of scalar fields $F,G\in \Gamma \Lambda ^0U$ according to

71 $$\begin{aligned} \sum _\mu \big (X_\mu \wedge Y_\mu )\langle F,G\rangle = \sum _\mu ( X_\mu \langle F\rangle Y_\mu \langle G\rangle - X_\mu \langle G\rangle Y_\mu \langle F\rangle \big ). \end{aligned}$$

Similarly we may define a vector field using

72 $$\begin{aligned} \sum _\mu (X_\mu \wedge Y_\mu )\langle F,\bullet \rangle = \sum _\mu \big ( X_\mu \langle F\rangle Y_\mu - \ Y_\mu \langle F\rangle X_\mu \big ). \end{aligned}$$

In the case that we have a simple bivector acting on a scalar field, we write

73 $$\begin{aligned} (X\wedge Y)\langle F,\bullet \rangle = X\langle F\rangle Y- Y\langle F\rangle X. \end{aligned}$$

#### Lemma 3.4

Given a bivector $\Phi$ and a nonzero vector field $X\in \Gamma TN$, $X\wedge \Phi =0$ if and only if there exists some $Y\in \Gamma \ TN$ such that $\Phi =X\wedge Y$.

#### Proof

Suppose that $X\wedge \Phi =0$ and let *N* have dimension $\ell$. Define a local coordinate system such that $X=\partial _0$ when we may write $\Phi =\Phi ^{0a}\partial _0\wedge \partial _a+\frac{1}{2} \Phi ^{bc}\partial _b \wedge \partial _c$, where $a,b,c=1,\ldots ,\ell -1$. Since $\partial _0\wedge \Phi = \frac{1}{2} \Phi ^{bc}\partial _0\wedge \partial _b\wedge \partial _c=0$, we must have that each $\Phi ^{bc}=0$. Hence, $\Phi = \partial _0\wedge (\Phi ^{0a}\partial _a)=X\wedge Y$. The converse holds by the properties of the exterior product ($X\wedge X=0$). $\hfill\square$

#### Definition 3.5

*(Specially Related Pairs of Vector Fields)* Two pairs of vector fields $X_1,X_2\in \Gamma TN$ and $Y_1,Y_2\in \Gamma TN$ are said to be *specially related* if there exists $\alpha ,\beta ,\gamma ,\delta \in \Gamma \Lambda ^0N$ satisfying

$$\begin{aligned} ( \alpha \delta - \beta \gamma )\vert _p=1\; \forall p\in N, \end{aligned}$$

such that

$$\begin{aligned} \begin{aligned} Y_1=\alpha X_1+\beta X_2, \text { and } Y_2=\gamma X_1+\delta X_2. \end{aligned} \end{aligned}$$

#### Lemma 3.6

Two pairs of vector fields, $X_1,X_2\in \Gamma TN$ and $Y_1,Y_2\in \Gamma TN$, are specially related if and only if $X_1\wedge X_2= Y_1\wedge Y_2$.

#### Proof

See Sect. A.2. $$\hfill\square$$

For a Vlasov bivector to contain all the necessary information to perform plasma kinematics, it must satisfy three conditions: the radial condition, the horizontal condition, and a third condition. There are three equivalent ways to express the third condition: integrability, being radially cubic, and being expressible as in Eq. (70).

We begin by discussing integrability. A geometric interpretation of a bivector is a network of infinitesimal rectangles whose sides are defined by a pair of vectors. When these bivectors ‘knit together’ to form smooth surfaces, the vector distribution spanned by the components of the bivector is integrable. These surfaces are depicted in Fig. 5. More formally, we may consider integrability using the Frobenius theorem and the language of vector distributions (a method of smoothly assigning vector subspaces of $T_xM$ to each point $x\in M$).

#### Definition 3.7

*(Tangent Bivector)* A bivector $\Phi \in \Gamma \mathcal {B}^2(N)$ is tangent to a surface $K\subset N$ if, given a representation for the bivector $\Phi =X\wedge Y, \; X,Y\in \Gamma TN$, both *X* and *Y* are tangent to *K* in the sense described in Definition 1.2.

From Lemma 3.6, it is clear that Definition 3.7 is independent of the representation of the bivector: if $\Phi =X\wedge Y$ and $\Phi =Z\wedge V$ and *X* and *Y* are tangent to *K*, then by Lemma 3.6*Z* and *V* are linear combinations of *X* and *Y* and hence tangent to *K*.

#### Definition 3.8

*(Integrability)* A simple bivector $\Phi \in \Gamma \mathcal {B}^2(N)$ is said to be *integrable* if there exists $X,Y\in \Gamma TN$ and $\alpha ,\beta \in \Gamma \Lambda ^0N$ such that if $\Phi =X\wedge Y$ then $[X,Y]=\alpha X+ \beta Y$. By Lemma 3.6, if one representation of $\Phi$ satisfies the integrability condition, then so do all other representations of $\Phi$.

#### Lemma 3.9

If a bivector $\Phi \in \Gamma \mathcal {B}^2(N)$ is integrable then for any representation $\Phi =X\wedge Y,\; X,Y\in \Gamma TN$, we have

74 $$\begin{aligned} {[}X,Y]=\alpha X+ \beta Y, \end{aligned}$$

for some $$\alpha ,\beta \in \Gamma \Lambda ^0N$$.

#### Proof

See Sect. A.3. $$\hfill\square$$

It follows from Lemmas 3.6 and 3.9 that integrability is a well-defined property. That is, given a simple bivector, if one representation satisfies the integrability condition, then all representations do.

The Frobenius theorem states that a vector distribution over a manifold *M* is integrable if and only if the Lie bracket of any two vectors within the distribution also lies within the distribution. An integrable vector distribution then admits a collection of maximal connected integral manifolds which form a foliation of *M*. Given an integrable bivector $\Phi =X\wedge Y$, we can form a vector distribution over *U* which is spanned by *X* and *Y*. This generates a two-dimensional foliation of *U* such that *X* and *Y* are tangent to the leaves of our foliation at each point. We may identify the leaves of this foliation with the form manifolds of the particle density form.

We also introduce the null condition here. This is a property an arbitrary bivector may have which is necessary for defining the transport equations on *U*.

#### Definition 3.10

*(Null condition)* Given a $(2n{-}2)$-form $\alpha$, and a bivector $\Phi \in \Gamma \mathcal {B}^2(N)$, then *the null condition* is given by

75 $$\begin{aligned} \begin{gathered} \text{ Null }(\Phi ,\alpha ) \text { is true}\iff i_X\alpha =0\;\text {and}\; i_Y\alpha =0, \text{ for } \text{ any } X,Y\in \Gamma TN \text{ such } \text{ that } \Phi = X \wedge Y. \end{gathered} \end{aligned}$$

To see that the null condition is well defined notice that if $X\wedge Y= Z\wedge V$ then $\text{ Null }(X\wedge Y,\alpha )$ holds if and only if $\text{ Null } (Z\wedge V,\alpha )$ holds. This is due to Lemma 3.6 and the linearity of the contraction mapping.

### Horizontal bivectors

The radial and the horizontal conditions are dealt with in tandem. We now exclusively work on the submanifold $U\subset \breve{T}M$.

#### Definition 3.11

*(Radial Bivectors)* A simple bivector $\Phi \in \Gamma \mathcal {B}^2(U)$ is called *radial* if

76 $$\begin{aligned} \mathcal {R}\wedge \Phi =0, \end{aligned}$$

where $\mathcal {R}\in \Gamma TU$ is the radial vector field.

#### Definition 3.12

*(Horizontal Bivectors)* A simple radial bivector $\Phi \in \Gamma \mathcal {B}^2(U)$ is called *horizontal* if for any $f,h\in \Gamma \Lambda ^0M$ it satisfies

77 $$\begin{aligned} \Phi \langle \pi ^*f, \dot{h}\rangle = -\dot{f}\dot{h}. \end{aligned}$$

The space of horizontal bivectors is denoted $\Gamma \mathcal {B}^2_H(U)$.

Note that in order for a bivector to be horizontal it must also be simple and radial. Hence, from this point onwards, when we refer to a horizontal bivector, we also assume it to be simple and radial.

#### Lemma 3.13

A bivector $\Phi =\mathcal {R}\wedge X$ is horizontal if and only if $X\in \Gamma TU$ is horizontal.

#### Proof

Suppose first that $\Phi = \mathcal {R}\wedge X$ for some horizontal $X\in \Gamma TU$. We then have $\mathcal {R}\wedge \Psi =0$ automatically; hence, $\Phi$ is radial. To see that $\Phi$ is horizontal, observe that

$$\begin{aligned} \begin{aligned} \Phi \langle \pi ^*f,\dot{h}\rangle =&\mathcal {R} \langle \pi ^*f \rangle X\langle \dot{h} \rangle - \mathcal {R} \langle \dot{h} \rangle X\langle \pi ^*f \rangle = -\dot{f}\dot{h}, \end{aligned} \end{aligned}$$

for any $$f,h\in \Gamma \Lambda ^0M$$.

Suppose now that $\Phi$ is horizontal. By Lemma 3.4 (since $\Phi$ is radial and simple), there exists some $X\in \Gamma TU$ such that

$$\begin{aligned} \Phi = \mathcal {R}\wedge X. \end{aligned}$$

By the horizontal condition

$$\begin{aligned} \begin{aligned} -\dot{f}\dot{h}= \Phi \langle \pi ^*f,\dot{h}\rangle =&\mathcal {R} \langle \pi ^*f \rangle X\langle \dot{h} \rangle - \mathcal {R} \langle \dot{h} \rangle X\langle \pi ^*f \rangle = -X\langle \pi ^*f \rangle \dot{h}, \end{aligned} \end{aligned}$$

for any $f,h\in \Gamma \Lambda ^0M$. Hence, $X \langle \pi ^*f \rangle =\dot{f}$, that is, *X* is horizontal. $\hfill\square$

#### Lemma 3.14

Let $X\in \Gamma TU$ and $\Phi \in \Gamma \mathcal {B}^2(U)$ such that $\Phi =\mathcal {R}\wedge X$. If *X* is horizontal and $\Phi$ is integrable, then there exists $\alpha \in \Gamma \Lambda ^0U$ such that

78 $$\begin{aligned} {[}\mathcal {R},X]=X+\alpha \mathcal {R}. \end{aligned}$$

#### Proof

Since *X* is horizontal, observe that for any $f\in \Gamma \Lambda ^0M$ we have

$$\begin{aligned} \begin{aligned} {[}\mathcal {R},X]\langle \pi ^*f\rangle =&\mathcal {R}\langle X\langle \pi ^*f\rangle \rangle - X\langle \mathcal {R}\langle \pi ^*f \rangle \rangle = \mathcal {R}\langle \dot{f}\rangle =\dot{f}=X\langle \pi ^*f\rangle . \end{aligned} \end{aligned}$$

By the integrability of $\Phi ,$ we also have $[\mathcal {R},X]=\alpha \mathcal {R}+ \beta X$ so that we also have

$$\begin{aligned} {[}\mathcal {R},X]\langle \pi ^*f\rangle = \alpha \mathcal {R}\langle \pi ^*f\rangle +\beta X\langle \pi ^*f \rangle = \beta X\langle \pi ^*f \rangle . \end{aligned}$$

Combining the last two equations gives us that $\beta =1$. Hence, the result holds. $\hfill\square$

### Vlasov bivectors

With this we have all the necessary ingredients to define a Vlasov bivector. There are several equivalent properties which ensure that a bivector contains sufficient structure. Given a horizontal bivector $\Psi \in \Gamma \mathcal {B}^2_H(U)$, these three properties are, the existence of a Vlasov field $W\in \Gamma TU$ such that $\Psi =\mathcal {R}\wedge W$, integrability, and another property we call the radially cubic property. Representations and integrability are discussed in Sect. 3.1, and the radially cubic property is discussed below.

#### Definition 3.15

*(Radially Cubic)* A horizontal bivector $\Psi \in \Gamma \mathcal {B}^2_H(U)$ is *radially cubic* if for any $f,h\in \Gamma \Lambda ^0 M$, $\underline{u}\in U$, and $\lambda \ne 0$ we have

79 $$\begin{aligned} \Psi \langle \dot{f},\dot{h} \rangle \vert _{\lambda \underline{u}}= \lambda ^3 \Psi \langle \dot{f},\dot{h} \rangle \vert _{\underline{u}}. \end{aligned}$$

We see below in Theorem 3.18 that we can generate a Vlasov field *W*, using a Vlasov bivector and a kinematic indicator. A natural choice to use is a ‘lab-time’ kinematic indicator $\dot{t}$. However, in general there is no $t\in \Gamma \Lambda ^0M$ such that $\dot{t}\ne 0$ on all of *U*. As an intermediate step, we can always define the following kinematic indicator using the coordinate system $(x^0,\ldots x^{n-1})$ on *M*,

80 $$\begin{aligned} F_{crd }= \sum _{\mu =0}^{n-1} (\dot{x}^\mu )^2. \end{aligned}$$

It is clear this is not a physical kinematic indicator since it depends on the coordinate system. It is homogeneous degree 2. Since this is even, one would need the causality indicator to define a kinematic domain, Eq. (36).

#### Definition 3.16

*(Coordinate-based Vlasov field)* Let $(x^0,\ldots x^{n-1})$ be a local coordinate system on *M* and let $\Psi \in \Gamma \mathcal {B}_H^2(U)$, we define the *coordinate-based Vlasov field* by

81 $$\begin{aligned} W_{crd }= \frac{\Psi \langle F_{crd },\bullet \rangle }{2F_{crd }}. \end{aligned}$$

#### Lemma 3.17

Let $\Psi \in \Gamma \mathcal {B}^2_H(U)$ be radially cubic and then $W_{crd }$ as given by Eq. (81) is a Vlasov field such that

82 $$\begin{aligned} \Psi =\mathcal {R}\wedge W_{crd }. \end{aligned}$$

Furthermore, given another representation for $\Psi$, say $\Psi =\mathcal {R}\wedge X$ for some $X\in \Gamma TU$, then

83 $$\begin{aligned} W_{crd }=X- \frac{X\langle F_{crd }\rangle }{2F_{crd }}\mathcal {R}. \end{aligned}$$

#### Proof

See Sect. A.3$$\hfill\square$$

#### Theorem 3.18

Let $\Psi \in \Gamma \mathcal {B}^2_H(U)$ be a horizontal bivector. The following properties are equivalent:

(i) There exists a Vlasov field $W\in \Gamma TU$ such that $\Psi =\mathcal {R}\wedge W$.
(ii) $\Psi$ is integrable (Definition 3.8).
(iii) $\Psi$ is radially cubic (Definition 3.15).

#### Proof

Suppose first that there exists a Vlasov field *W* such that $\Psi = \mathcal {R}\wedge W$. To see that $\Psi$ is radially cubic observe that, for some $f,h\in \Gamma \Lambda ^0M$, any $\underline{u}\in U$, and $\lambda \ne 0$, it follows that

$$\begin{aligned} \begin{aligned} \Psi \langle \dot{f},\dot{h} \rangle \vert _{\lambda \underline{u}}&= \mathcal {R} \langle \dot{f} \rangle W\langle \dot{h} \rangle \vert _{\lambda \underline{u}}- \mathcal {R} \langle \dot{h} \rangle W\langle \dot{f} \rangle \vert _{\lambda \underline{u}} = \lambda \mathcal {R} \langle \dot{f} \rangle \lambda ^2 W\langle \dot{h} \rangle \vert _{\underline{u}} -\lambda \mathcal {R} \langle \dot{h} \rangle \lambda ^2 W_i\langle \dot{f} \rangle \vert _{\underline{u}} = \lambda ^3 \Psi \langle \dot{f},\dot{h} \rangle \vert _{\underline{u}}. \end{aligned} \end{aligned}$$

Also, since *W* is radially quadratic we have $[\mathcal {R},W]=W$ by Lemma 2.4. Hence, $\Psi$ is integrable. That is, property (i) implies properties (ii) and (iii).

By Lemma 3.17, if $\Psi$ is radially cubic then $W_{crd }$ as defined by Eq. (81) is a Vlasov field and $\Psi =\mathcal {R}\wedge W_{crd }$. Hence, property (iii) implies (i).

Suppose now that $\Psi$ is integrable. Let $W_{crd }$ be given by Eq. (81). By Lemma 3.17, $\Psi =\mathcal {R}\wedge W_{crd }$ and $W_{crd }$ is horizontal. It remains to show that $W_{crd }$ is radially quadratic. By Lemma 3.13, there exists a horizontal vector field $X\in \Gamma TU$ such that $\Psi =\mathcal {R}\wedge X$ and by Lemma 3.17, $W_{crd }$ and *X* are related by Eq. (83). The integrability of $\Psi$ tells us $[\mathcal {R},X]=\alpha \mathcal {R}+ \beta X$ for some $\alpha ,\beta \in \Gamma \Lambda ^0 U$. It follows that $\beta =1$ form Lemma 3.14. We then have that

$$\begin{aligned} \begin{aligned} [\mathcal {R},W_{crd }]&= [\mathcal {R},X]- \left[ \mathcal {R},\frac{X\langle \dot{f}\rangle }{\dot{f}} \mathcal {R} \right] = [ \mathcal {R},X] -\mathcal {R} \bigg \langle \frac{X\langle \dot{f}\rangle }{\dot{f}} \bigg \rangle \mathcal {R} = X+ \alpha \mathcal {R} -\left( \frac{\mathcal {R}\langle X\langle \dot{f}\rangle \rangle }{\dot{f}}- \frac{X\langle \dot{f}\rangle }{\dot{f}} \right) \mathcal {R} \\{}&= X+\alpha \mathcal {R} -\frac{1}{\dot{f}} [\mathcal {R},X]\langle \dot{f}\rangle \mathcal {R} = X+\alpha \mathcal {R} -\frac{1}{\dot{f}} (X\langle \dot{f}\rangle +\alpha \dot{f})\mathcal {R} = X-\frac{X\langle \dot{f}\rangle }{\dot{f}}\mathcal {R} = W_{crd }. \end{aligned} \end{aligned}$$

Hence, $W_{crd }$ is radially quadratic by Lemma 2.4. It follows that $W_{crd }$ is a Vlasov field and property (ii) implies property (i). $\hfill\square$

#### Definition 3.19

*(Vlasov Bivectors)* A horizontal bivector $\Psi \in \Gamma \mathcal {B}^2_H(U)$ defines a *Vlasov bivector* if it satisfies one of the following equivalent properties:

1. There exists a Vlasov field $W\in \Gamma TU$ such that $\Psi =\mathcal {R}\wedge W$.
2. $\Psi$ is integrable (Definition 3.8).
3. $\Psi$ is radially cubic (Definition 3.15).

The space of Vlasov vectors over *U* is denoted by $\Psi \in \Gamma \mathcal {B}^2_V(U)$.

#### Lemma 3.20

Let $\Psi \in \Gamma \mathcal {B}_V^2(U)$, let *E* have a kinematic indicator *F* of order *k*, and let

84 $$\begin{aligned} {W}_F=\frac{\Psi \left\langle {F},\bullet \right\rangle }{k F}, \end{aligned}$$

Then $W_F$ is a Vlasov field which is compatible with *F* and

85 $$\begin{aligned} \Psi = \mathcal {R}\wedge W_F. \end{aligned}$$

#### Proof

$\Psi =\mathcal {R}\wedge W_F$ and $W_F$ is horizontal by the same logic as Lemma 3.17. Furthermore, by Lemma 3.17, $\Psi =\mathcal {R}\wedge W_{crd }$ where $W_{crd }$ is given by eq. (81). To see that $W_F$ is radially quadratic observe that

$$\begin{aligned} \begin{aligned} W_F\vert _{\lambda \underline{u}} \langle \dot{f}\rangle&= \left( \frac{\Psi \langle F,\dot{f}\rangle }{kF} \right) \bigg \vert _{\lambda \underline{u}} = \left( \frac{\mathcal {R}\langle F\rangle W_{crd }\langle \dot{f}\rangle - \mathcal {R}\langle \dot{f}\rangle W_{crd }}{kF} \right) \bigg \vert _{\lambda \underline{u}} = \left( W_{crd }\langle \dot{f}\rangle -\frac{\dot{f}}{kF} W_{crd }\langle F\rangle \right) \bigg \vert _{\lambda \underline{u}}\\ {}&= \left( \lambda ^2 W_{crd }\langle \dot{f}\rangle -\frac{\lambda \dot{f}}{\lambda ^k kF} \lambda ^{k+1} W_{crd }\langle F\rangle \right) \bigg \vert _{\underline{u}} =\lambda ^2 \left( W_{crd }\langle \dot{f}\rangle -\frac{ W_{crd }\langle F\rangle }{ kF} \mathcal {R}\langle \dot{f} \rangle \right) \bigg \vert _{\underline{u}}\\&=\lambda ^2 W_F\vert _{\underline{u}}\langle \dot{f}\rangle . \end{aligned} \end{aligned}$$

The Vlasov field $W_F$ is compatible with *F*, i.e. $W_F\langle F\rangle =0$, since $\Psi \langle F,F\rangle =0$. It follows that

$$\begin{aligned} \begin{aligned} \frac{\Psi \langle F,\bullet \rangle }{kF}=&\frac{\Psi \langle F,\bullet \rangle }{\mathcal {R}\langle F\rangle }= \frac{\mathcal {R}\langle F\rangle W_F- W_F\langle F\rangle \mathcal {R}}{\mathcal {R}\langle F\rangle }= W_F. \end{aligned} \end{aligned}$$

$$\hfill\square$$

With Lemmas 3.6 and 3.9 combined, we may note that Vlasov bivectors constructed from projectively related Vlasov fields represents the same object; that is, if there exists a 1-homogeneous function $k\in \Gamma \Lambda ^0 U$ such that $\hat{W}=W+k\mathcal {R}$, then $\Psi =\mathcal {R}\wedge W= \mathcal {R} \wedge \hat{W}$. In Fig. 5, the foliations generated by $\mathcal {R},W$ are the same of those generated by $\mathcal {R},\hat{W}$. We can use the Vlasov bivector to derive the transformation formula Eq. (54).

#### Lemma 3.21

If $\Psi =\mathcal {R}\wedge W= \mathcal {R} \wedge \hat{W}$ and $\hat{W}$ is compatible with kinematic indicators $\hat{F}$, then Eq. (54) holds.

#### Proof

Consider the action of $\Psi$ on the scalar field $\hat{F}$:

$$\begin{aligned} \begin{aligned} \Psi \langle \hat{F},\bullet \rangle =&\mathcal {R}\langle \hat{F} \rangle W- W\langle \hat{F}\rangle \mathcal {R} = \mathcal {R}\langle \hat{F} \rangle \hat{W}- \hat{W}\langle \hat{F}\rangle \mathcal {R}= \mathcal {R}\langle \hat{F} \rangle \hat{W}. \end{aligned} \end{aligned}$$

Hence, Eq. (54) $$\hfill\square$$

Here we show that the Vlasov Bivector $\Psi$, knit together as leaves, as depicted in Fig. 5. Since the leaves are tangent to $\mathcal {R}$, then they must open out like a book.

#### Theorem 3.22

Given a Vlasov bivector $\Psi$, then for each $\underline{u}\in U$ there exists a two-dimensional surface $K\subset U$ such that $\underline{u}\in K$ and $\Psi$ is a tangent to *K*.

#### Proof

From Theorem 3.18, we can write $\Psi =\mathcal {R}\wedge W$ such that $\mathcal {R}$ and *W* are in involution, Eq. (29). The result now follows directly from Frobenius theorem, which can be found in [31]. $\hfill\square$

## Particle density $(2n{-}2)$-forms

In this section, we introduce the particle density $(2n{-}2)$-form on *U*, $\theta \in \Gamma \Lambda ^{2n-2}U$. This may be depicted pictorially in Figs. 1 and 5. It is the generalisation of the particle density form $\theta _E\in \Gamma \Lambda ^{2n-2}E$, and it is subject to the transport equations on *U*, defined below. This reformulation of the transport equations has the same advantages as mentioned in Introduction.

### Transport equations on *U*

#### Definition 4.1

*(Transport Equations on U)* Given a particle density form $\theta \in \Gamma \Lambda ^{2n-2}U$ and a Vlasov bivector $\Psi \in \Gamma \mathcal {B}^2_V(U)$, the *transport equations on* *U* are given by

86 $$\begin{aligned} \text{ Null }(\Psi ,\theta )\; \text{ holds } \text{ and } \; d\theta =0, \end{aligned}$$

where the null condition is given by Definition 3.10.

Notice that if $\text{ Null }(\Psi ,\theta )$ holds and $\Psi =\mathcal {R}\wedge W$ then both *W* and $\mathcal {R}$ are tangent to the form manifolds of $\theta$. That is, $i_W\theta =0$ and $i_{\mathcal {R}}\theta =0$. This is consistent with the visualisation in Fig. 5. We can identify the form manifolds of $\theta$ with the leaves of a foliation generated by a Vlasov bivector. Since $\theta$ is a closed form, the form manifolds associated with it are smooth surfaces. Furthermore, the velocity density profile of the particle distribution is reflected in these form manifolds: the closer together the surfaces, the greater the local velocity density of the particles (and vice versa).

#### Definition 4.2

*(Populated Systems on U)* Given a Vlasov bivector $\Psi$ and a particle density $(2n{-}2)$-form $\theta \in \Gamma \Lambda ^{2n-2} U$ which satisfies the transport equations on *U* Eq. (86), we define the pair $(\Psi ,\theta )$ to be a populated system on *U*.

As stated in Introduction, defining a populated system does not require a time orientation. Since for any given radial $\{\lambda \underline{u}\in U, \lambda >0\}$ is disconnected from $\{\lambda \underline{u}\in U, \lambda <0\},$ there is no relationship between $\theta \vert _{\underline{u}}$ and $\theta \vert _{-\underline{u}}$. For example, in Eq. (70) the particle density form can be considered nonzero on $U^+$ and zero on $U^-$.

### Relating the particle density on *U* with the particle density on *E*

For this subsection, we assume that *U* is time-orientable and that there is a kinematic domain *E*. We relate the particle densities $\theta \in \Gamma \Lambda ^{n-2}U$ and $\theta _E\in \Gamma \Lambda ^{n-2}E$. Furthermore, we assume that $\theta$ only contains particles which lie on $U^+$.

#### Definition 4.3

*(Future Pointing Particle Density)* A particle density form $\theta \in \Gamma \Lambda ^{(2n-2)}U$ is called future pointing if *U* is time-orientable and

87 $$\begin{aligned} \theta \vert _{U^-}=0. \end{aligned}$$

For future pointing particle densities, we let the restriction $\theta ^+\in \Gamma \Lambda ^{2n-2}U^+$ be

88 $$\begin{aligned} \theta ^+ = \theta \vert _{U^+}. \end{aligned}$$

A populated system $(\Psi ,\theta ^+)$ formed from a future pointing particle density is called a future pointing populated system.

#### Lemma 4.4

Let $(\Psi ,\theta ^+)$ define a future pointing populated system on *U*, satisfying the transport equations. Given a kinematic domain $E\subset U^+$ with kinematic indicator *F*, let $W_E$ be given by Eq. (32) where *W* is given by Eq. (84), and let $\theta _E\in \Gamma \Lambda ^{2n-2}E$ be given by

89 $$\begin{aligned} \theta _E=\Sigma _E^*\theta ^+. \end{aligned}$$

Then $W_E$ and $\theta _E$ satisfy the transport equations on *E*.

#### Proof

Observe that

$$\begin{aligned} d\theta _E=&d\Sigma _E^*\theta = \Sigma _E^*d\theta =0, \; \text{ and } \; i_{W_E}\theta _E= i_{W_E} \Sigma _E^*\theta = \Sigma _E^*\left( i_W\theta \right) =0. \end{aligned}$$

Hence, $\theta _E$ with $W_E$ satisfy Definition 2.19. $\hfill\square$

#### Lemma 4.5

Given a kinematic domain *E* with $\theta _E\in \Gamma \Lambda ^{2n-2}E$ and $W_E\in \Gamma TE$ such that $\theta _E$ satisfies the transport equations on *E*, define the map,

90 $$\begin{aligned} \Pi _E :U^+\rightarrow E;&\quad \Pi (\lambda \underline{v})=\underline{v} \; \text{ where } \; \underline{v}\in E and \lambda >0. \end{aligned}$$

Let

91 $$\begin{aligned} \theta ^+= \Pi _E^*\theta _E, \end{aligned}$$

let $\theta$ be future pointing, given by Eqs. (87) and (88), and let $W\in \Gamma TU$ be given by Eq. (32). The system $(\Psi =\mathcal {R}\wedge W,\theta )$ satisfies the transport equations on *U*.

#### Proof

See Sect. A.3. $$\hfill\square$$

#### Lemma 4.6

Given a kinematic domain *E* and particle densities $\theta _E$ and $\theta$ where $\theta$ is future pointing satisfying the relevant transport equations, then Eq. (89) holds if and only if Eq. (91) holds.

#### Proof

See Sect. A.3. $$\hfill\square$$

A direct application of Lemmas 4.4 and 4.5 to numerical simulations is given in Sect. 5.2, where they are used to construct a particle density function in a new kinematic as a way to deduce quantities in a boosted frame.

### The current associated with the particle density form

For a populated system on *E*, $(W_E,\theta _E)$ can construct the current $\mathcal {J}_E$ associated with $\theta _E$ by integrating over each fibre. In differential geometry, this integration can be expressed very naturally using the language of de Rham pushforwards. The conservation of charge $d \mathcal {J}_E=0$ follows from the transport equation $d \theta _E=0$ and the fact the exterior derivative commutes with the de Rham pushforward.

To write the current density for a populated system on *U*, it is necessary to introduce a support 1-form $\chi$. The goal of this subsection is to first define the current form $\mathcal {J}$ on *U*, then show that the result is independent of the choice of this support 1-form, and to show charge is conserved.

#### Definition 4.7

*(Integration along a fibre)* Let $\pi :K\rightarrow M$ where *K* is an oriented *k*-dimensional vector bundle over *M*. A form $\alpha \in \Gamma \Lambda ^qK$ (for $n\le k \le 2n$) is said to have vertical compact support if for each $p\in M$ the restriction $\alpha \vert _{\pi ^{-1}(p)}$ has compact support. Given a form with vertical compact support $\alpha \in \Gamma \Lambda ^q K,$ the *integral along the fibre* (otherwise known as the de Rham pushforward) $\pi _\varsigma \alpha \in \Gamma \Lambda ^{n-r} M$ is defined by

92 $$\begin{aligned} \int \limits _K \pi ^*\beta \wedge \alpha = \int \limits _M\beta \wedge \pi _\varsigma \alpha , \end{aligned}$$

for all forms $\beta \in \Gamma \Lambda ^r M$ with compact support such that $q+r =\dim (K)$. For a comprehensive overview, see [32].

#### Definition 4.8

*(Current forms from E)* Let *U* be time-orientable, let $E\subset U^+$ be a kinematic domain and let $\theta _E\in \Gamma \Lambda ^{2n-2}E$ define a particle density $2n{-}2$-form satisfying the transport equations on *E*. We define the current $(n{-}1)$-form $\mathcal {J}_E\in \Gamma \Lambda ^{n-1}M$ by

93 $$\begin{aligned} \mathcal {J}_E= \pi _{E\varsigma }(\theta _E). \end{aligned}$$

Here, $\pi _E:E\rightarrow M$ is the projection from the bundle *E* to *M*.

Since the above definition relies on a choice of kinematic domain *E*, we propose the following generalisation. For this, we need to define a support form, which is a 1-form on *U*.

#### Definition 4.9

*(Support Form)* Given any $\underline{u}\in U$ let $\mathfrak {R}_{\underline{u}}=\{ \lambda \underline{u} \; :\; \lambda >0\}$ and $\hat{\mathfrak {R}}_{\underline{u}} :\mathbb {R}^+ \hookrightarrow U$. A *support form* $\chi \in \Gamma \Lambda ^1 U$ is a 1-form such that for all $\underline{u}\in U$, $\hat{\mathfrak {R}}_{\underline{u}}^*\chi$ has compact support on $\mathfrak {R}_{\underline{u}}$ and satisfies

94 $$\begin{aligned} \int \limits _{\mathbb {R}^+} \hat{\mathfrak {R}}_{\underline{u}}^*\chi =1, \end{aligned}$$

for each $$\underline{u}\in U$$.

Observe that replacing $\underline{u}$ with $\lambda \underline{u}$ for some $\lambda >0$ does not change the integration in Eq. (94). Recall that $\theta \vert _{\underline{u}}$ may be unrelated to $\theta \vert _{-\underline{u}}$ and in general both can be nonzero. Thus, we need a support form to have support on both $\mathfrak {R}_{\underline{u}}$ and $\mathfrak {R}_{-\underline{u}}$ so that both sides contribute.

#### Definition 4.10

*(Current forms from U)* Let $\theta \in \Gamma \Lambda ^{2n-2}U$ be a particle density form satisfying the transport equations on *U* (Definition 4.1). The *current form on U*, $\mathcal {J}\in \Gamma \Lambda ^{n-1}M$ is given by

95 $$\begin{aligned} \mathcal {J}=\pi _\varsigma (\chi \wedge \theta ), \end{aligned}$$

for any support form $\chi \in \Gamma \Lambda ^1U$.

Although we need to impose additional structure through the inclusion of the support form, we may show that the current form from *U* is independent of our choice of support form.

#### Lemma 4.11

Let *N* be an $\ell$-dimensional manifold, $\alpha \in \Gamma \Lambda ^{\ell -1}N$, $\beta \in \Gamma \Lambda ^1N$, $t\in \Gamma \Lambda ^0N$ and $X\in \Gamma TN$ such that

96 $$\begin{aligned} L_X\alpha =0,\quad i_X\alpha =0,\quad \text{ and } \quad X\langle t\rangle =1. \end{aligned}$$

Let $K_{t_0}=\{ p\in N :t\vert _p=t_0 \}$ for some value $t_0$ and define the embedding $\Sigma _{t_0}:K_{t_0}\hookrightarrow N$. Lastly, let $\eta _p(t)$ denote the integral curve of *X* passing through *p*. Then

97 $$\begin{aligned} \int \limits _N \alpha \wedge \beta = \int \limits _{p\in K_{t_0}} \Sigma _{t_0}^*\alpha \left( \int \limits _{\mathbb {R}} \eta _{p}^*\beta \right) , \end{aligned}$$

provided $\eta _{p}^*\beta$ has compact support on the domain of each $\eta _p$.

#### Proof

See Sect. A.3. $$\hfill\square$$

#### Lemma 4.12

Let the current form $\mathcal {J}\in \Gamma \Lambda ^{n-1}M$ as given by Definition 4.10. $\mathcal {J}$ is independent of the choice of support form $\chi \in \Gamma \Lambda ^1U$.

#### Proof

Observe that since $\theta$ satisfies the transport equations (Definition 4.1) we have $i_{\mathcal {R}}\theta =0$ and $d\theta =0$ so $L_{\mathcal {R}}\theta =0$. It follows that

$$\begin{aligned} L_{\mathcal {R}}(\pi ^*\phi \wedge \theta )= \pi ^*(L_{\pi _*\mathcal {R}}\phi )\wedge \theta -\pi ^*\phi \wedge L_{\mathcal {R}} \theta =0, \end{aligned}$$

since $\pi _*\mathcal {R}=0$, for any test form $\phi$. We also have $i_{\mathcal {R}}(\pi ^*\phi \wedge \theta )=0$ so we may apply Lemma 4.11 in the following way. Let $r\in \Gamma \Lambda ^0 U$ such that $dr\ne 0$ and $\mathcal {R}\langle r \rangle =1$, then let $\eta _{\underline{u}}$ be an integral curve of $\mathcal {R}$ passing through $\underline{u}\in U$ and let $K_{r_0}=\{ \underline{u}\in U :r\vert _{\underline{u}}=r_0 \}$ with $\Sigma _{r_0}:K_{r_0} \hookrightarrow U$ for some value $r_0$. We then have

$$\begin{aligned} \begin{aligned} \int \limits _U \pi ^*\phi \wedge \theta \wedge \chi&= \int \limits _{\underline{u}\in K_{r_0}} \Sigma _{r_0}^*(\pi ^*\phi \wedge \theta ) \left( \int \limits _{\mathbb {R}} \eta _{\underline{u}}^*\chi \right) =\int \limits _{K_{r_0}} \Sigma _{r_0}^*(\pi ^*\phi \wedge \theta ). \end{aligned} \end{aligned}$$

Hence, $\mathcal {J}$ is independent of the choice of support form $\chi$. $\hfill\square$

#### Lemma 4.13

The current $(n{-}1)$-form on *U*, $\mathcal {J}=\pi _\varsigma (\chi \wedge \theta )$, satisfies the continuity equation

98 $$\begin{aligned} \text {d}\mathcal {J}=0. \end{aligned}$$

#### Proof

The exterior derivative commutes with the de Rham pushforward and $d\theta =0$ so we have

$$\begin{aligned} \text {d}\mathcal {J}= \text {d}\pi _\varsigma (\chi \wedge \theta )= \pi _\varsigma (\text {d}\chi \wedge \theta ). \end{aligned}$$

Since $\mathcal {J}$ is independent of our choice of $\chi$ by Lemma 4.12 it suffices to pick $\chi$ such that $d\chi =0$. By picking $r\in \Gamma \Lambda ^0 U$ such that $dr\ne 0$ and $\mathcal {R}\langle r \rangle =1$, we may define a coordinate system $(x^\mu ,r,y^{a})$. By choosing

$$\begin{aligned} \chi =\chi _r(r)\text {d}r, \end{aligned}$$

where $\chi _r(r)$ is a function in *r* with compact support, to comply with Eq. (94), we have $d\chi =0$. Hence, $d\mathcal {J}=0$, and charge is conserved. $\hfill\square$

#### Lemma 4.14

Let *U* be time-orientable, *E* be a kinematic domain and $\theta$ is future time pointing. Let $\theta$ and $\theta _E$ be related by Eqs. (89) and (91). The current form on *U*, $\mathcal {J}\in \Gamma \Lambda ^{n-1}M$ (Definition 4.10) and the current form on *E*, $\mathcal {J}_E\in \Gamma \Lambda ^{n-1}M$, Definition 4.8, are identical.

#### Proof

Let *F* be the 1-homogeneous kinematic indicator associated with *E*, define

$$\begin{aligned} r=\log F, \end{aligned}$$

$K_{r_0}=\{ \underline{u}\in U :r\vert _{\underline{u}}=r_0 \}$, and $\Sigma _{r_0}:K_{r_0}\hookrightarrow U$. First observe that $E=K_0$ and $\mathcal {R}\langle r\rangle =1$. By application of Lemma 4.11, we have for any test form $\phi \in \Gamma _0\Lambda ^1M$,

$$\begin{aligned} \begin{aligned} \int \limits _U \pi ^*\phi \wedge \theta \wedge \chi&= \int \limits _{p\in K_0} \Sigma _0^*(\pi ^*\phi \wedge \theta ) \left( \int \limits _{\mathbb {R}} \eta _p^*\chi \right) = \int \limits _{ K_0} \Sigma _0^*(\pi ^*\phi \wedge \theta ) = \int \limits _{ E} \Sigma _E^*(\pi ^*\phi \wedge \theta ) = \int \limits _E \pi _E^*\phi \wedge \theta _E. \end{aligned} \end{aligned}$$

Hence, for any *E*, $\mathcal {J}=\mathcal {J}_E$. $\hfill\square$

Using the coordinate expression for the fibre integral in [33], the coefficients of the current vector on the unit hyperboloid $E_{\text {H}}$ (i.e. the 4 current when $\dim M =4$) are given by

99 $$\begin{aligned} \mathcal {J}_{E_{\text {H}}}^{\mu }=\int \limits _{E_{\text {H}}}\dot{x}^\mu f_{E_{\text {H}}} \frac{\sqrt{-\det g}}{\dot{x}_0} {\text {d}}\dot{x}^{1,\ldots ,n-1}, \end{aligned}$$

where

100 $$\begin{aligned} \mathcal {J}_{E_{\text {H}}}^\mu = \star ({\text {d}}x^\mu \wedge \mathcal {J}_{E_{\text {H}}}). \end{aligned}$$

### Discussion about the stress–energy $n{-}1$-form

Given a kinematic domain *E* and $\alpha \in \Gamma \Lambda ^1M$, the stress–energy $(n{-}1)$-form can be expressed

101 $$\begin{aligned} \tau ^E_{\alpha }= \pi _E{}_\varsigma (\hat{\alpha } \theta _E), \end{aligned}$$

where $\hat{\alpha }\in \Gamma \Lambda ^0U$ is given by $\hat{\alpha }\vert _{\underline{u}}=\alpha :\underline{u}$ for $\underline{u}\in U$. The stress–energy $n{-}1$-form for the unit hyperboloid $E_{\text {H}}$ can be converted into the usual stress–energy tensor

102 $$\begin{aligned} T_{E_{\text {H}}}^{\mu \nu }=\int \limits _{E_{\text {H}}}\dot{x}^\mu \dot{x}^\nu f_{E_{\text {H}}} \frac{\sqrt{-\det g}}{\dot{x}_0} {\text {d}}\dot{x}^{1,\ldots ,n-1}, \end{aligned}$$

using the relationship

103 $$\begin{aligned} T_{E_{\text {H}}}^{\mu \nu } = \star ({\text {d}}x^\mu \wedge \tau ^{E_{\text {H}}}_{{\text {d}}x^\nu }), \end{aligned}$$

where $\star$ is the Hodge dual. See Lemma A.8 for proof of this statement. This can be substituted into the right-hand side of Einstein’s equations to complete the Einstein–Vlasov system on $E_{\text {H}}$.

Due to the similarities between the definitions of the current $(n{-}1)$-form, Eq. (93), and the stress–energy $(n{-}1)$-form, Eq. (101)), it may be tempting to try defining the stress–energy $(n{-}1)$-form on *U* by

104 $$\begin{aligned} \tau = \pi _\varsigma (\chi \wedge \hat{\alpha }\theta ). \end{aligned}$$

Unfortunately, unlike for $\mathcal {J}$, the stress–energy $(n{-}1)$-form depends on the choice of $\chi$. This is because $d\left( \hat{\alpha } \theta \right) \ne 0$ in general, so that Lemma 4.14 does not apply.

For an explicit example of $\tau ^E_{\alpha }$ depending on *E*, consider the charged free dust in Minkowski space time so that in Cartesian coordinates $W=\dot{x}^\mu \partial ^{(x)}_\mu$. In this case, $\tau ^{E_{\text {H}}}_{\alpha }$ and $\tau ^{E_{t}}_{\alpha }$ are given by

105 $$\begin{aligned} \tau ^{E_{\text {H}}}_{\alpha }&= i^{(x)}_\mu {\text {d}}x^{0,\cdots ,n-1} \int \limits _{E_{\text {H}}} f_{E_{\text {H}}} \alpha _{a} \frac{\dot{x}^{a}}{\dot{x}_0} \dot{x}^\mu {\text {d}}\dot{x}^{1,\cdots ,n-1},\end{aligned}$$

106 $$\begin{aligned} \tau ^{E_{t}}_{\alpha }&= i^{(x)}_\mu {\text {d}}x^{0,\cdots ,n-1} \int \limits _{E_{t}} f_{E_{t}} \alpha _{a} \dot{x}^{a} \dot{x}^\mu {\text {d}}\dot{x}^{1,\cdots ,n-1}. \end{aligned}$$

in their respective adapted coordinate systems.

Furthermore, in general, the stress–energy form is not necessarily divergenceless (in the sense put forth in [34]) for an arbitrary lab frame with no electromagnetic interaction. Using the external covariant derivative *D*,

107 $$\begin{aligned} D \tau ^E_{\alpha }= {\text {d}} \tau ^E_{\alpha } - {\text {d}}x^\mu \wedge \tau ^E_{\nabla _\mu \alpha }, \end{aligned}$$

the divergenceless condition can then be written $D \tau ^E_{\alpha }=0$. In [35] it’s shown that $D \tau ^{E_{\text {H}}}_{\alpha }=0$. However, for an arbitrary lab bundle $E_{t}$, it can be shown that

108 $$\begin{aligned} D \tau ^{E_{t}}_{{\text {d}}x^{\underline{\mu }}}=0, \quad D\tau ^{E_{t}}_{{\text {d}}x^0}= {\text {d}}x^{0,\cdots ,n-1} \int _{\pi ^{-1}(\bullet )} \Gamma ^0_{\mu \nu } \dot{x}^\nu \dot{x}^\mu {\text {d}}\dot{x}^{1,\cdots ,n-1}. \end{aligned}$$

in an adapted coordinate system $(x^\mu ,\dot{x}^{a})$.

The usual stress–energy form is $\tau ^{E_{\text {H}}}_{\alpha }$, which is needed for the Einstein–Vlasov system, is divergenceless. This implies there is a preferred kinematic domain, $E_{\text {H}}$. One may ask whether there are still advantages for using the formalism in this article. We aregue that there are for two reasons. First, it makes it clear how the Vlasov equations, i.e. the Vlasov bivector and the transport equations, depend on the metric. This may be less explicit in the usual treatment. This is especially relevant if one needs to vary the metric, as is done in [36], for instance. Second, it reveals the relationship between the stress–energy tensor and the kinematic domain. Since we do not have a formulation of the stress–energy form/tensor which does not depend on the kinematic domain, we cannot assert that Einstein–Vlasov systems are parameter-free under our formalism.

Another use of our approach, is when considering non-metric compatible connections. As stated in the introduction, the trajectories no longer remain on $E_{\text {H}}$, see Fig. 2. However, using the Vlasov bivector and the particle density form $\theta$, this is no longer a problem. After calculating $\theta$, one can choose $E_{\text {H}}$ to calculate $\tau ^{E_{\text {H}}}_{\alpha }$.

It is an open question to see whether there is a relationship between the stress–energy forms for different *E*’s or $\chi$’s or whether there exists an object similar to the stress–energy form can be defined which does not depend on the choice of *E* or $\chi$.

## Application to numerical simulation of plasmas

This work can be applied to numerical simulations of plasmas. Such simulations typically involve solving the Maxwell–Vlasov system, either directly (particularly in one or two dimensions), or through PIC methods.

The usual method for describing the relativistic Maxwell–Vlasov system is in terms of proper time. However, for numerical simulations it is much easier to use the lab time. This is explained in Sect. 5.1.

A particular numerical simulation procedure is the agile numerical integrator (ANI). To date, this method is mainly used where velocities are non-relativistic. If velocities start approaching the speed of light, simulation becomes increasingly challenging as it would require many grid points near $v=c$. We show how one can extend the applicability of the ANI, using a series of Lorentz transformations, to scenarios where the velocities of particles become large, but the relative velocities between the particles remains small. This is ideal for cases of particle acceleration, as is detailed in Sect. 5.2.

### The use of lab time

**Fig. 7:**
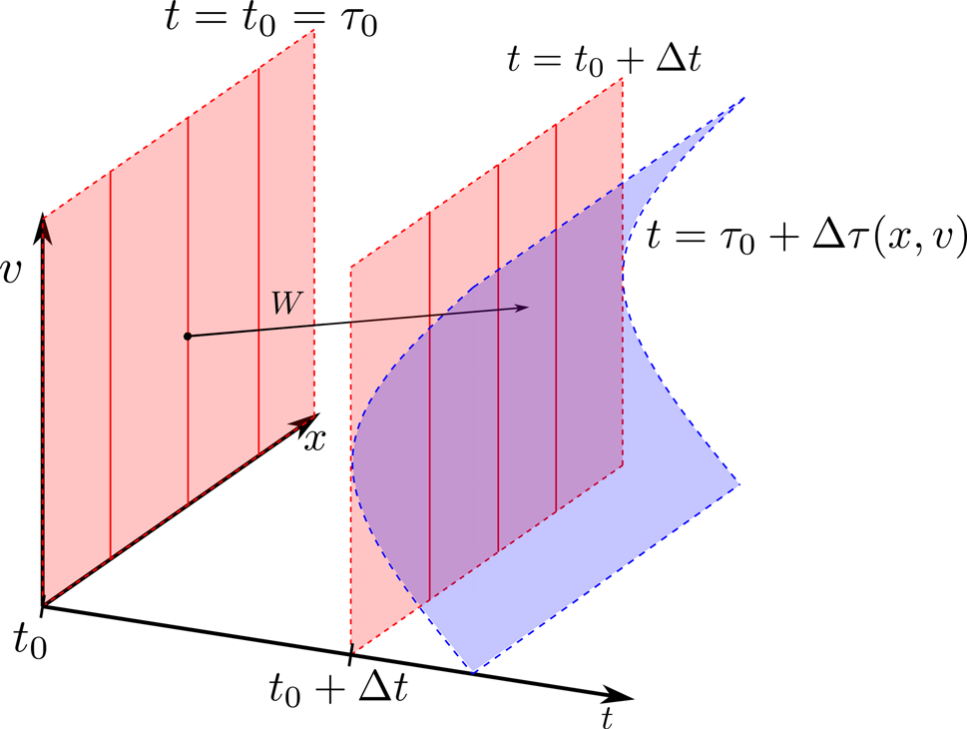
Advantages of using a lab frame when doing numerical simulations. The red planes represent time slices. We integrate over *v* to evaluate the charge and current. These are the fibres depicted in dark red. By contrast, using proper time to step forward from $\tau _0=t_0$ to $\tau _0+\Delta \tau _0$ results in the blue surface which cannot be directly integrated

These simulations invariably use a lab frame. Recall that the term lab frame in this article refers to any kinematic domain $E_t$, which is determined by a time-slicing scalar field $t\in \Gamma \Lambda ^0M$. This may be the frame of an actual lab, but can include the rest frame of a particle.

It is challenging to use the proper-time Lorentz force equations to update the particle trajectory. This is because, assuming $x^0(\tau )$ all coincided, the updated $x^0(\tau +\Delta \tau )$ would not coincide. This would make it difficult to sum over the particles in the same cell to obtain the charge or current. The same problem arises when attempting to solve the Maxwell–Vlasov system with the proper-time version of Vlasov’s equation, since the integration over velocities cannot be performed directly. This is depicted in Fig. 7.

Assuming a numerical simulation scheme is using a lab frame $E_t$, one needs to choose time-slicing scalar field *t*. This decision is made alongside many others, such as coordinates, grid strategy, integrator, etc. The usual choice for Minkowski spacetime is an inertial frame. However, there may be advantages with using a non-inertial frame. For example, in a particle accelerator, one could use the frame adapted to the design orbit. For uniform acceleration, this would correspond to Rindler coordinates.

In general relativity, there are generally no preferred coordinate systems, and the choice of time-slicing may be arbitrary. In this case, it may be useful to be able to run the simulations in different coordinate systems and compare the result.

Let *t* be one time-slicing and $\hat{t}=\hat{t}(t,x^a)$ be a new time-slicing. Then from Eq. (12)

109 $$\begin{aligned} \dot{\hat{t}} = \frac{\partial \hat{t}}{\partial t} \dot{t} + \frac{\partial \hat{t}}{\partial x^a} \dot{x}^a, \end{aligned}$$

since $\dot{\hat{t}}$ is a kinematic indicator it can be directly placed into Eq. (54) to give the transformed Vlasov field.

### The agile numerical integrator

Our formalism enables a generalisation of work such as [11] wherein a novel integration scheme, the agile numerical (ANI), is derived from the treatment of the Vlasov field as the divergence of a flux density $\mathcal {J}$ over phase space. As stated in the introduction, the ANI is mainly used to model particles with non-relativistic velocities. Here we show how to extend, using a Lorentz transformation, the ANI to higher velocities, giving it application for particle accelerators, or even the electrons inside the bubble of a wakefield accelerator.

In the aforementioned paper, the linearised Vlasov–Poisson system is described by the flux density

110 $$\begin{aligned} \mathcal {J} = f \hat{t} + v_x f\hat{x} - \frac{q}{m} \frac{\partial \phi }{\partial x}f \hat{v}_x, \end{aligned}$$

where $\hat{t}$ (resp. $\hat{x},\;\hat{v}_x$) are unit vectors in the *t* (resp. *x*, $v_x$) direction, and $\phi$ is the electric potential satisfying the Poisson equation. This enables the Vlasov equation to be cast as

111 $$\begin{aligned} \nabla \cdot \mathcal {J} =0, \end{aligned}$$

or an equivalent flux integral.

We can identify the coefficients of the phase space flux density Eq. (110) with the coefficients of the particle density form Definition 2.19. In particular, assuming the Vlasov–Poisson system to be written on a lab-time bundle $E_{t}$, we may make the following identifications: $v_x=\dot{x}$, $\hat{t}=\partial _t$, $\hat{x}=\partial _x$, $\hat{v}_x=\partial _{\dot{x}},$ and $\Omega _{E_{t}}={\text {d}}t\wedge {\text {d}}x\wedge d\dot{x}$ which satisfies Eq. (56). This allows us to recognise the notion of a velocity field on a phase space $\underline{u}$ from [11] with our notion of a Vlasov field $W_{E_{t}}$ on a kinematic domain $E_{t}$. From here we can make the identification,

112 $$\begin{aligned} \mathcal {J}=f_{E_{t}}W_{E_{t}}. \end{aligned}$$

Consequently Eq. (111) is equivalent to the transport equations Eq. (58), and the Vlasov equation can be written as

113 $$\begin{aligned} L_{W_{E_{t}}}\left( f_{E_{t}} \Omega _{E_{t}} \right) = 0. \end{aligned}$$

Consequently, our formalism allows for the generalisation of the ANI. We illustrate this through an application by performing a Lorentz boost to the Vlasov–Poisson system.

The main benefit of the ANI is that by characterising the Vlasov equation as a vector field, the particle density function (PDF) can be evolved independently along each dynamical axis. This allows us to write the equation for the evolution of the PDF with fewer interpolations than a method invoking Strang splitting. For the relativistic Vlasov–Poisson system, the discretised Vlasov equation yields

114 $$\begin{aligned} f(t+\Delta t,x,\dot{x})= f(t,x-\dot{x}\Delta t, \dot{x}) +f\left( t,x,\dot{x}-\frac{q}{m}\mathcal {E} F_{\text {H}}^{3/2} \Delta t \right) -f(t,x,\dot{x}), \end{aligned}$$

where $F_{\text {H}}$ is given by Eq. (20), which is equal to $F_{\text {H}}=1-\dot{x}^2$ here, and $\mathcal {E}$ is the electric field.

Note that this scheme requires 2 interpolations, one in *x* and one in $\dot{x}$. By gridding phase space in intervals width $(\Delta t, \Delta x, \Delta \dot{x})$ and indexing the nodes on the grid such that $f^n_{j,k}= f(n\Delta t, j\Delta x, k\Delta \dot{x})$, the above equation can be written

115 $$\begin{aligned} f^{n+1}_{j,k}= \sum _{j'} \mu ^0_{j,j'}(x-\dot{x}\Delta t)\ f^n_{j',k} + \sum _{k'} \mu ^1_{k,k'}\left( \dot{x}-\frac{q}{m}\mathcal {E} F_{\text {H}}^{3/2} \Delta t \right) \ f^n_{j,k'} -f^n_{j,k} \end{aligned}$$

where the $\mu ^0_{j,j'}(x)$ and $\mu ^1_{k,k'}(\dot{x})$ are the interpolation parameters, e.g. [37].

The number of interpolations can be halved by defining the grid points as $x=j\Delta x$ and $\dot{x}=k \Delta {x}/\Delta t$. Substituting into (114) gives

116 $$\begin{aligned} f^{n+1}_{j,k}= f^n_{j-k,k} + \sum _{k'} \mu ^1_{k,k'}\left( \dot{x}-\frac{q}{m}\mathcal {E} F_{\text {H}}^{3/2} \Delta t \right) \ f^n_{j,k'} -f^n_{j,k}. \end{aligned}$$

We can still obey the Courant stability conditions, by choosing $\Delta t$ such that $\Delta t \le \Delta x /V_0$ and $\Delta t \le (\Delta x/(\frac{q}{m} {\mathcal {E}_0} F_H^{3/2}))^{1/2}$, where $V_0$ is the maximum velocity of the system, and $\mathcal {E}_0$ is the maximum electric field amplitude. When the above grid spacing is chosen, we call this the matched grid case.

As stated in the introduction, if the particles become ultra-relativistic so that $\dot{x}\rightarrow c$, the use of the ANI becomes challenging as, to resolve the velocities, we would need many velocity grid points near $\dot{x}=c$. Using Eq. (115), this is possible, but not ideal, whereas using Eq. (116) this is not possible as the grid size $\Delta \dot{x}$ is fixed. Assuming the energy spread of the particles remains small, the solution is to perform a Lorentz boost, and continue the simulation in a grid adapted to this new rest frame. We show here how our technology can be used to transform the Vlasov field $W_{E_{t}}$ and the PDF $f_{E_{t}}$ to the new frame.

Suppose we have a Vlasov–Poisson system on the lab bundle $E_{t}$ with PDF $f_{E_{t}}$, and corresponding Vlasov field $W_{E_{t}}$. To find the equivalent of the PDF evolution equation [Eq. (114)] in the boosted frame $E_{t'}$, we first need to deduce $W_{E_{t'}}$ using Theorem 2.18. Next, we find an appropriate choice of $\Omega$ to calculate $\theta _{E_{t}}$ using Eq. (57) and use this to deduce $\theta _{E_{t'}}$ by promoting $\theta _{E_{t}}$ to $\theta ^+$ using Lemma (4.5). Then we use $\theta ^+$ to calculate $\theta _{E_{t'}}$ through Lemma (4.6), which gives us a relationship between $f_{E_{t}}$ and $f_{E_{t'}}$ using Eq. (57).

Using Eq. (34), we can write the 1D1V Vlasov–Poisson field on *U* (the light cone), in a coordinate system adapted to $E_{t}$, $(t,x,\dot{t},\dot{x})$, using the Lorentz metric $g_{\mu \nu }=\text{ diag }(-1,1)$ as

117 $$\begin{aligned} W=\dot{t}\partial _t +\dot{x}\partial _x -\frac{q}{m}\mathcal {E}\frac{F_H^{3/2}}{\dot{t}} \partial _{\dot{x}}, \end{aligned}$$

where $\mathcal {E}=\mathcal {E}(t,x)$ is the electric field, and $F_H$ is Eq. (20) (here, $F_{\text {H}}= \dot{t}^2 -\dot{x}^2$). Note that *W* is horizontal and vertically quadratic. Since $W\langle \dot{t}\rangle =0$, *W* is tangent to $E_{t}$ by Lemma A.5, we can write

118 $$\begin{aligned} W_{E_{t}}= \partial _t + \dot{x}\partial _x -\frac{q}{m}\mathcal {E}(F_H\circ \Sigma _{E_{t}})^{3/2}\partial _{\dot{x}}, \end{aligned}$$

since $\Sigma _{E_{t}}{}_*(W_{E_{t}})=W\vert _{E_{t}}$. That is, *W* is the extension of $W_{E_{t}}$. This is consistent with the 1D1V Vlasov field used to derive Landau oscillations (before linearisation) when $F_H=1$ in the non-relativistic case. Consider now a Lorentz boost to a frame moving with constant velocity *V*, $(t,x)\mapsto (t',x')=(\gamma t-V\gamma x, \gamma x- V\gamma t)$, where $\gamma =(1-V^2)^{-1/2}$. It is straightforward to show that on *U* we have

119 $$\begin{aligned} {}&\dot{t}'= \gamma \dot{t}-V\gamma \dot{x},\quad \dot{x}'=\gamma \dot{x}-V\gamma \dot{t}, \quad \dot{t}= \gamma \dot{t}' +\gamma V \dot{x}', \quad \dot{x}= \gamma \dot{x}' +\gamma V\dot{t}', \end{aligned}$$

120 $$\begin{aligned} {}&\partial _t=\gamma \partial _{t'}-\gamma V\partial _{x'},\quad \partial _x=\gamma \partial _{x'}-\gamma V\partial _{t'}, \quad \partial _{\dot{t}} = \gamma \partial _{\dot{t}'} -\gamma V\partial _{\dot{x}'}, \quad \partial _{\dot{x}} =\gamma \partial _{\dot{x}'}-\gamma V\partial _{\dot{t}'}. \end{aligned}$$

The lab bundle associated with this boost is given by $E_{t'}$. Using Eq. (54) and the above, we get

121 $$\begin{aligned} \begin{aligned} W'&= W-\frac{W\langle \dot{t}'\rangle }{\mathcal {R}\langle \dot{t}'\rangle } \mathcal {R} = \dot{t}\partial _t+\dot{x}\partial _x -\frac{q}{m} \mathcal {E} \frac{F_H^{3/2}}{\dot{t}} \partial _{\dot{x}} -\frac{q}{m}\mathcal {E} \frac{V\gamma F_H^{3/2}}{\dot{t}'\dot{t}}\mathcal {R} = \dot{t}'\partial _{t'}+ \dot{x}'\partial _{x'} -\frac{q}{m}\mathcal {E}\frac{F_H^{3/2}}{\dot{t}'}\partial _{\dot{x}'}. \end{aligned} \end{aligned}$$

From the same reasoning as for Eq. (118), it follows that

122 $$\begin{aligned} W_{E_{t'}}= \partial _{t'} +\dot{x}'\partial _{x'} -\frac{q}{m}\mathcal {E} (F_{\text {H}}\circ \Sigma _{E_{t'}})^{3/2}\partial _{\dot{x}'}, \end{aligned}$$

where $(t',x',\dot{t}',\dot{x}')$ is the coordinate system adapted to $E_{t'}$.

To get the particle density function in the new frame, we first need the correct volume element $\Omega \in \Gamma \Lambda ^4U$,

123 $$\begin{aligned} \Omega = \frac{-\det g}{F_H^{3/2}} {\text {d}}t\wedge {\text {d}}x\wedge {\text {d}}\dot{t}\wedge {\text {d}}\dot{x}. \end{aligned}$$

To see that $L_W\Omega =0,$ observe that (noting $\det g =-1$)

124 $$\begin{aligned} \begin{aligned} L_W\Omega&= {\text {d}} i_W\Omega ={\text {d}} \left( \frac{1}{F_H^{3/2}} \left( \dot{t} {\text {d}}x\wedge {\text {d}}\dot{t}\wedge {\text {d}}\dot{x} -\dot{x} {\text {d}}t\wedge {\text {d}}\dot{t}\wedge {\text {d}}\dot{x} +\frac{q}{m}\mathcal {E} \frac{F_H^{3/2}}{\dot{t}} {\text {d}}t\wedge {\text {d}}x\wedge {\text {d}}\dot{t} \right) \right) \\ {}&= \left( \partial _t\dot{t} +\partial _x\dot{x} -\frac{q}{m}\mathcal {E}\partial _{\dot{x}}\frac{1}{\dot{t}} \right) {\text {d}}t\wedge {\text {d}}x\wedge {\text {d}}\dot{t}\wedge {\text {d}}\dot{x}=0. \end{aligned} \end{aligned}$$

A similar calculation shows $L_{W'}\Omega =0$. From Lemma 2.22, by setting $s=t$ and $\alpha = (F_H\circ \Sigma _{E_{t}})^{-3/2} dx\wedge d\dot{x}$, and noting that $L_{\mathcal {R}}\Omega =-\Omega$ we see that $\Omega$ is unique.

To write $\theta _{E_{t}}$ we first calculate $i_{W_{E_{t}}}\Sigma _E^*i_{\mathcal {R}}\Omega$:

125 $$\begin{aligned} \begin{aligned} i_{\mathcal {R}}\Omega&= \frac{\dot{t}}{F_H^{3/2}} {\text {d}}t\wedge {\text {d}}x\wedge {\text {d}}\dot{x} -\frac{\dot{x}}{F_H^{3/2}} {\text {d}}t\wedge {\text {d}}x\wedge {\text {d}}\dot{t},\\ \Sigma _{E_{t}}^*i_{\mathcal {R}}\Omega&= \frac{1}{(F_{\text {H}}\circ \Sigma _{E_{t}})^{3/2}} {\text {d}}t\wedge {\text {d}}x\wedge {\text {d}}\dot{x},\\ i_{W_{E_{t}}} \Sigma _{E_{t}}^*i_{\mathcal {R}}\Omega&= \frac{1}{(F_{\text {H}}\circ \Sigma _{E_{t}})^{3/2}} {\text {d}}x\wedge {\text {d}}\dot{x} -\frac{\dot{x}}{(F_{\text {H}}\circ \Sigma _{E_{t}})^{3/2}} {\text {d}}t\wedge {\text {d}}\dot{x} -\frac{q}{m}\mathcal {E} {\text {d}}t\wedge {\text {d}}x. \end{aligned} \end{aligned}$$

Hence, we may write

126 $$\begin{aligned} \begin{aligned} \theta _{E_{t}}&=f_{E_{t}} i_{W_{E_{t}}}\Sigma _{E_{t}}^*i_{\mathcal {R}}\Omega \\ {}&= \frac{f_{E_{t}}}{(F_{\text {H}}\circ \Sigma _{E_{t}})^{3/2}} \left( {\text {d}}x\wedge {\text {d}}\dot{x} -\dot{x}{\text {d}}t\wedge {\text {d}}\dot{x} -\frac{q}{m} \mathcal {E} (F_{\text {H}}\circ \Sigma _{E_{t}})^{3/2} {\text {d}}t\wedge {\text {d}}x \right) . \end{aligned} \end{aligned}$$

Recall the definition for $\Pi _{E_{t}}: U^+\rightarrow E_{t}$ from Eq. (90). Using this, we can define $\theta ^+=\Pi _{E_{t}}^*\theta _{E_{t}}$. Define coordinates on $E_{t}$ by $(t_{E_{t}},x_{E_{t}},\dot{x}_{E_{t}})$ (note that this is the same as $(t,x,\dot{x})$ in previous and subsequent equations due to abuse of notation). Using the fact that $(t,x,\dot{t},\dot{x})\mapsto (t_{E_{t}},x_{E_{t}},\dot{x}_{E_{t}})$ where $\Sigma _{E_{t}}(t_{E_{t}},x_{E_{t}},\dot{x}_{E_{t}})= (t,x,1,\dot{x}/\dot{t})$,

127 $$\begin{aligned} {}&\Pi _{E_{t}}^*({\text {d}}\dot{x})={\text {d}}\left( \frac{\dot{x}}{\dot{t}} \right) = \frac{1}{\dot{t}}{\text {d}}\dot{x} -\frac{\dot{x}}{\dot{t}^2} {\text {d}}\dot{t}\end{aligned}$$

128 $$\begin{aligned} \implies&\theta ^+= \frac{f_{E_{t}}\circ \Pi _{E_{t}}}{(F_{\text {H}}\circ \Sigma _{E_{t}}\circ \Pi _{E_{t}})^{3/2}} \left( {\text {d}}x\wedge \left( \frac{1}{\dot{t}}{\text {d}}\dot{x}-\frac{\dot{x}}{\dot{t}^2}{\text {d}}\dot{t} \right) - \frac{\dot{x}}{\dot{t}}{\text {d}}t\wedge \left( \frac{1}{\dot{t}}{\text {d}}\dot{x} -\frac{\dot{x}}{\dot{t}^2} {\text {d}}\dot{t} \right) -\frac{q}{m} \mathcal {E} (F_{\text {H}}\circ \Sigma _{E_{t}}\circ \Pi _{E_{t}})^{3/2}) {\text {d}}t\wedge {\text {d}}x \right) \end{aligned}$$

129 $$\begin{aligned} {}&\quad \;= \frac{f_{E_{t}}\circ \Pi _{E_{t}}}{(F_{\text {H}}\circ \Sigma _{E_{t}}\circ \Pi _{E_{t}})^{3/2}} \left( \frac{1}{\dot{t}} {\text {d}}x\wedge {\text {d}}\dot{x} -\frac{\dot{x}}{\dot{t}^2}{\text {d}}x\wedge {\text {d}}\dot{t} -\frac{\dot{x}}{\dot{t}^2}{\text {d}}t\wedge {\text {d}}\dot{x} +\frac{\dot{x}^2}{\dot{t}^3}{\text {d}}t\wedge {\text {d}}\dot{t} -\frac{q}{m} \mathcal {E} (F_{\text {H}}\circ \Sigma _{E_{t}}\circ \Pi _{E_{t}})^{3/2} {\text {d}}t\wedge {\text {d}}x \right) . \end{aligned}$$

Now we perform the coordinate transformation corresponding to the Lorentz boost:

130 $$\begin{aligned} \begin{aligned} \frac{1}{\dot{t}} {\text {d}}x\wedge {\text {d}}\dot{x}&= \frac{\gamma ^2}{\dot{t}} ( {\text {d}}x'+ V{\text {d}}t')\wedge ({\text {d}}\dot{x}'+ V {\text {d}}\dot{t}')\\ {}&= \frac{\gamma ^2}{\dot{t}} ({\text {d}}x'\wedge {\text {d}}\dot{x}' +V{\text {d}}x'\wedge {\text {d}}\dot{t}' +V{\text {d}}t'\wedge {\text {d}}\dot{x}' +V^2{\text {d}}t'\wedge {\text {d}}\dot{t}'),\\ -\frac{\dot{x}}{\dot{t}^2} {\text {d}}x\wedge {\text {d}}\dot{t}&= -\frac{\dot{x}\gamma ^2}{\dot{t}^2} ({\text {d}}x' +V{\text {d}}t')\wedge ({\text {d}}\dot{t}'+V{\text {d}}\dot{x}')\\ {}&= -\frac{\dot{x}\gamma ^2}{\dot{t}^2} ({\text {d}}x'\wedge {\text {d}}\dot{t}' +V{\text {d}}x'\wedge {\text {d}}\dot{x}' +V{\text {d}}t'\wedge {\text {d}}\dot{t}' +V^2{\text {d}}t' \wedge {\text {d}}\dot{x}'),\\ -\frac{\dot{x}}{\dot{t}^2}{\text {d}}t \wedge {\text {d}}\dot{x}&= -\frac{\dot{x}\gamma ^2}{\dot{t}^2} ({\text {d}} t' +V{\text {d}}x') \wedge ({\text {d}}\dot{x}'+V{\text {d}}\dot{t}')\\ {}&= -\frac{\dot{x}\gamma ^2}{\dot{t}^2}({\text {d}}t'\wedge {\text {d}}\dot{x}'+V{\text {d}}t'\wedge {\text {d}}\dot{t}' +V{\text {d}}x'\wedge {\text {d}}\dot{x}'+ V^2 {\text {d}}x'\wedge {\text {d}}\dot{t}'),\\ \frac{\dot{x}^2}{\dot{t}^3} {\text {d}}t\wedge {\text {d}}\dot{t}&= \frac{\dot{x}^2\gamma ^2}{\dot{t}^3} ({\text {d}}t'+ V{\text {d}}x')\wedge ({\text {d}}\dot{t}'+ V{\text {d}}\dot{x}') \\ {}&= \frac{\dot{x}^2\gamma ^2}{\dot{t}^3} ({\text {d}}t'\wedge {\text {d}}\dot{t}' +V{\text {d}}t'\wedge {\text {d}}\dot{x}' +V{\text {d}}x'\wedge {\text {d}}\dot{t}' +V^2{\text {d}}x'\wedge {\text {d}}\dot{x}') \\ -\frac{q}{m} \mathcal {E} (F_{\text {H}}\circ \Sigma _{E_{t}}\circ \Pi _{E_{t}})^{3/2} {\text {d}}t\wedge {\text {d}}x&= -\frac{q}{m} \mathcal {E} (F_{\text {H}}\circ \Sigma _{E_{t}}\circ \Pi _{E_{t}})^{3/2}\gamma ^2({\text {d}}t'+V{\text {d}}x') \wedge ({\text {d}}x'+V{\text {d}}t')\\ {}&= -\frac{q}{m} \mathcal {E} (F_{\text {H}}\circ \Sigma _{E_{t}}\circ \Pi _{E_{t}})^{3/2} {\text {d}}t'\wedge {\text {d}}x'. \end{aligned} \end{aligned}$$

Gathering terms gives

131 $$\begin{aligned} {\text {d}}x'\wedge {\text {d}}\dot{x}':&\quad \frac{\gamma ^2}{\dot{t}}\left( 1 -2\frac{\dot{x}V}{\dot{t}} +\frac{V^2\dot{x}^2}{\dot{t}^2} \right) = \frac{\dot{t}^{\prime 2}}{\gamma ^3(\dot{t}'+ V\dot{x}')^3}\\ {\text {d}}x'\wedge {\text {d}}\dot{t}':&\quad \frac{\gamma ^2}{\dot{t}} \left( V -\frac{\dot{x}}{\dot{t}}(1+V^2) +\frac{V\dot{x}^2}{\dot{t}^2} \right) = \frac{-\dot{x}'\dot{t}'}{\gamma ^3(\dot{t}'+V\dot{x}')^3}\\ {\text {d}}t'\wedge {\text {d}}\dot{x}':&\quad \frac{\gamma ^2}{\dot{t}} \left( V- \frac{\dot{x}}{\dot{t}}(1+V^2) +\frac{\dot{x}^2V}{\dot{t}^2} \right) = \frac{-\dot{x}'\dot{t}'}{\gamma ^3(\dot{t}'+V\dot{x}')^3}\\ {\text {d}}t'\wedge {\text {d}}\dot{t}':&\quad \frac{\gamma ^2}{\dot{t}}\left( V^2 -2\frac{\dot{x}V}{\dot{t}} +\frac{\dot{x}^2}{\dot{t}^2} \right) = \frac{\dot{x}^{\prime 2}}{\gamma ^3(\dot{t}'+V\dot{x}')}\\ {\text {d}}t'\wedge {\text {d}}x':&\quad -\frac{q}{m} \mathcal {E}(F_{\text {H}}\circ \Sigma _{E_{t}}\circ \Pi _{E_{t}})^{3/2}. \end{aligned}$$

Therefore, $\theta ^+$ takes the form

132 $$\begin{aligned} \begin{aligned} \theta ^+&=\frac{f_{E_{t}}\circ \Pi _{E_{t}}}{(F_{\text {H}}\circ \Sigma _{E_{t}}\circ \Pi _{E_{t}})^{3/2}} \bigg ( \frac{\dot{t'}^2}{\gamma ^3(\dot{t}'+ V\dot{x}')^3} {\text {d}}x'\wedge {\text {d}}\dot{x}' +\frac{-\dot{x}'\dot{t}'}{\gamma ^3(\dot{t}'+V\dot{x}')^3} {\text {d}}x'\wedge {\text {d}}\dot{t}' +\frac{-\dot{x}'\dot{t}'}{\gamma ^3(\dot{t}'+V\dot{x}')^3}{\text {d}}t'\wedge {\text {d}}\dot{x}'\\ {}&\quad +\frac{\dot{x}^{\prime 2}}{\gamma ^3(\dot{t}'+V\dot{x}')}{\text {d}}t'\wedge {\text {d}}\dot{t}' -\frac{q}{m} \mathcal {E}(F_{\text {H}}\circ \Sigma _{E_{t}}\circ \Pi _{E_{t}})^{3/2}{\text {d}}t'\wedge {\text {d}}x' \bigg ). \end{aligned} \end{aligned}$$

Performing the pullback $\Sigma _{E_{t'}}^*$ on $\theta ^+$ gives

133 $$\begin{aligned} \begin{aligned} \theta _{E_{t'}}&= \frac{f_{E_{t}}\circ \Pi _{E_{t}} \circ \Sigma _{E_{t'}}}{(F_{\text {H}}\circ \Sigma _{E_{t}}\circ \Pi _{E_{t}}\circ \Sigma _{E_{t'}})^{3/2}} \bigg ( \frac{1}{\gamma ^3(1+ V\dot{x}')^3} {\text {d}}x'\wedge {\text {d}}\dot{x}' +\frac{-\dot{x}'}{\gamma ^3(1+V\dot{x}')^3}{\text {d}}t'\wedge {\text {d}}\dot{x}'\\ {}&\quad -\frac{q}{m} \mathcal {E}(F_{\text {H}}\circ \Sigma _{E_{t}}\circ \Pi _{E_{t}}\circ \Sigma _{E_{t'}})^{3/2}{\text {d}}t'\wedge {\text {d}}x'\bigg ). \end{aligned} \end{aligned}$$

To simplify observe that

134 $$\begin{aligned} \begin{aligned} \Sigma _{E_{t}}^*F_{\text {H}}&= 1-\dot{x}^2,\\ \Pi _{E_{t}}^*\Sigma _{E_{t}}^*F_{\text {H}}&= 1-\frac{\dot{x}^2}{\dot{t}^2}=\frac{-\dot{x}^{\prime 2}+\dot{t}^{\prime 2}}{\gamma ^2(\dot{t}' +V\dot{x}')^2},\\ \Sigma _{E_{t'}}^*\Pi _{E_{t}}^*\Sigma _{E_{t}}^*F_{\text {H}}&= \frac{1-\dot{x}^{\prime 2}}{\gamma ^2(1 +V\dot{x}')^2} =\frac{F_{\text {H}}\circ \Sigma _{E_{t'}}}{\gamma ^2(1 +V\dot{x}')^2}. \end{aligned} \end{aligned}$$

Plugging into our expression for $\theta _{E_{t'}}$ yields

135 $$\begin{aligned} \theta _{E_{t'}}= \frac{f_{E_{t}}\circ \Pi _{E_{t}}\circ \Sigma _{E_{t'}}}{(F_{\text {H}}\circ \Sigma _{E_{t'}})^{3/2}} \left( {\text {d}}x'\wedge {\text {d}}\dot{x}' -\dot{x'} {\text {d}}t'\wedge {\text {d}}\dot{x}' -\frac{q}{m}\mathcal {E} (F_{\text {H}}\circ \Sigma _{E_{t'}})^{3/2}{\text {d}}t'\wedge {\text {d}}x' \right) . \end{aligned}$$

Comparing this with the definition for $\theta _{E_{t'}}=f_{E_{t'}}i_{W_{E_{t'}}}\Sigma _{E_{t'}}^*i_{\mathcal {R}}\Omega$,

136 $$\begin{aligned} \theta _{E_{t'}}= \frac{f_{E_{t'}}}{(F_{\text {H}}\circ \Sigma _{E_{t'}})^{3/2}} \left( {\text {d}}x'\wedge {\text {d}}\dot{x}' -\dot{x}'{\text {d}}t'\wedge {\text {d}}\dot{x}' -\frac{q}{m} \mathcal {E}(F_{\text {H}}\circ \Sigma _{E_{t'}})^{3/2}{\text {d}}t'\wedge {\text {d}}x' \right) , \end{aligned}$$

allows us to make the identification

137 $$\begin{aligned} f_{E_{t'}}= f_{E_{t}}\circ \Pi _{E_{t}} \circ \Sigma _{E_{t'}}. \end{aligned}$$

By observing that

138 $$\begin{aligned} {}&\Sigma _{E_{t'}}:E_{t'} \hookrightarrow U ; (t',x',\dot{x}')\mapsto (t',x',1,\dot{x}')= (\gamma t -\gamma Vx, \gamma x-\gamma Vt,1, \gamma \dot{x} -\gamma V \dot{t}),\end{aligned}$$

139 $$\begin{aligned} {}&\Pi _{E_{t}} :U\rightarrow E_{t};(\gamma t -\gamma Vx, \gamma x-\gamma Vt, 1, \gamma \dot{x} -\gamma V \dot{t}) \mapsto \left( \gamma t -\gamma Vx, \gamma x-\gamma Vt, \gamma \dot{x} -\gamma V \right) , \end{aligned}$$

we may write

140 $$\begin{aligned} f_{E_{t'}}\left( t'(x,t),x'(x,t),\dot{x}'(\dot{x})\right) = f_{E_{t}}\left( \gamma t -\gamma Vx, \gamma x-\gamma Vt, \gamma \dot{x} -\gamma V \right) . \end{aligned}$$

It will now be necessary to create a new grid and redeposit the particles over this grid, using interpolation and Eq. (140). To do this, we require a new choice of grid spacing, a reasonable option for the matched grid case would be

141 $$\begin{aligned} \Delta t'= \gamma \Delta t, \quad \Delta x' = \frac{\Delta x}{\gamma }, \quad \Delta \dot{x}' = \frac{\Delta x'}{\Delta t'}= \frac{1}{\gamma ^2} \frac{\Delta x}{\Delta t}. \end{aligned}$$

On this new grid, we can recreate Eq. (114) using Eqs. (140) and (141) in order to execute the ANI in the new boosted bundle $E_{t'}$.

## Conclusion

In this article, we have presented an alternative way of representing the Vlasov equation. The primary advantage of this representation is that it enables the user to write the equations of motion without reference to a kinematic domain. As a result, the transformation laws of the Vlasov field become apparent and transformations between frames can be easily calculated in our framework.

The generality of our formalism makes it applicable to a wide variety of situations where the usual tools of kinematics would otherwise fail. For example, these tools apply in situations where there cannot exist a time orientation, when one does not have a metric compatible connection, or when working in a pre-metric context. Additionally, one can investigate the Vlasov equation for light-like particles. This enables the extension of the ultra-relativistic approximation [6] from fluids to the Vlasov equation. This is particularly relevant when considering particles in particle accelerators as well as astrophysical plasmas near black holes and neutron stars.

The current 3-form and the transport equations are generalised to our formalism. However, the generalisation of stress–energy 3-form depends on the choice of kinematic domain or a support form. Furthermore, the divergence of the stress–energy 3-form also depends on the choice of kinematic domain. As a result, it may be divergence-free with respect to one *E*, but not another. Since the stress–energy tensor is required for Einstein–Vlasov systems, it will be important, in future work, to investigate the relationship between $\tau ^E_{\alpha }$ and its divergence for different kinematic domains.

As highlighted in Sect. 5, our work has implications for numerical simulations, in particular when these simulations concern coordinate transformations, non-inertial frames or general relativity. The formulation of the (relativistic) Vlasov equation [38] in terms of the particle density form, i.e. the transport equations, allows for the generalisation of some existing numerical integrators; for example, the agile numerical integrator [11]. Our formalism enables the extension of the ANI to include ultra-relativistic scenarios, that is, where the velocities approach the speed of light. For example, when electrons are accelerated up to ultra-relativistic velocities, but the spread in their energies remains small. In Sect. 5.2, we show that this is achieved by performing a series of Lorentz boosts, so that the PDF is always in a frame where velocities are not close to the speed of light. These techniques may be generalised to other numerical integrators which see applications in accelerator physics. For future work, one can look at applying this technique to 2d Vlasov solvers. This will enable the modelling of the transfer for energy from high energy electrons to electromagnetic waves using a wiggler or undulator, generalising work such as [39].

There are many interesting plasma phenomena like Landau damping, two stream instability, and plasma echos which can be further investigated with these tools. The basic theory of these starts with a (relativistic) Poisson–Vlasov system, for example [38]. As such, they have a preferred inertial lab frame and corresponding time-slicing. The power of the results in this article will become apparent if one wishes to reformulated the Poisson–Vlasov systems in a non-inertial lab frame or with respect to a more complicated kinematic domain. For example, a scenario where there is a magnetic field in the inertial lab frame, but no magnetic field in an adapted co-moving accelerating lab frame.

There is clearly a deep relationship between the work presented here and the idea of trajectory and solutions of second-order ODEs which do not have a parameter prescribed. In a follow-up article, we will show how to define such trajectories and their connection to the leaves of the Vlasov bivector.

Other directions one may consider are to look at the relationship of this work with jet bundles and Finsler geometry. One can also look at how to generalise the Vlasov equation and the Boltzmann equation. In this latter case, we may have to replace the transport equation Sect. 86, with $\text {Null}(\Psi ,\theta )$ and $d\pi _\varsigma \theta =0$.

In summary, we argue that the Vlasov bivector is the fundamental object to describe kinetic systems, since it is invariant under reparameterisation.

## Data Availability

All data for this article is fully contained within this article.

## Appendices

### Sprays and semi-sprays

Vlasov fields can be formulated in terms of sprays and semi-sprays. For a detailed discussion of sprays the reader is directed towards [26] and [40]. For our purposes, we define a spray as follows.

#### Definition A.1

*(Spray)* A *spray* on a smooth manifold *N* is a smooth vector field $X\in \Gamma T(\breve{T}N)$ which is expressed in local adapted coordinates $(x^\mu ,\dot{x}^\mu )$ as

142 $$\begin{aligned} X=\dot{x}^\mu \frac{\partial \;}{\partial x^\mu } +X^\mu \frac{\partial \;}{\partial \dot{x}^\mu }, \end{aligned}$$

where $X^\mu = X^\mu (x,\dot{x})$ are local functions on $\breve{T}N$ satisfying

143 $$\begin{aligned} X^\mu \vert _{\lambda \underline{u}}=\lambda ^2 X^\mu \vert _{\underline{u}},\; \lambda >0,\; \underline{u}\in \breve{T}N. \end{aligned}$$

In the case where $\breve{T}N$ is a conic bundle, this can be shown to be equivalent to definition 2.3.

The trajectories of a spray are defined in the same way as described in section 2.3, satisfying the equation

144 $$\begin{aligned} \frac{d^2C^\mu }{dt^2} = X^\mu \left( C^\mu (t),\frac{dC^\mu }{dt} \right) . \end{aligned}$$

#### Definition A.2

*(Projectively related Sprays)* Two sprays *X* and $\hat{X}$ are *projectively related* if they have the same trajectories as point sets. That is, if *C*(*t*) is a trajectory of *X*, then there exists a reparameterisation $t=t(s)$ such that $C(s):=C(t(s))$ is a geodesic of $\hat{X}$ (and vice versa).

#### Lemma A.3

Two sprays *X* and $\hat{X}$ are projectively related if and only if there exists a 1-homogeneous scalar field $k\in \Gamma \Lambda ^0 (\breve{T}N)$ such that

145 $$\begin{aligned} \hat{X}= X+k \mathcal {R}, \end{aligned}$$

where $\mathcal {R}$ is the radial vector field on $\breve{T}N$.

#### Proof

See Z. Shen, Differential Geometry of Spray and Finsler Spaces, pages 173–174 [26], and Lemma 2.17. $\hfill\square$

In the literature, a semi-spray is defined similarly to a spray on $\breve{T}N$, only without the homogeneity property. For our purposes, it is productive to define sprays on some hypersurface $K\subset \breve{T}N$. We restrict our attention to hypersurfaces defined similarly to lab-time bundles.

#### Definition A.4

*(Semi-Spray)* Given $s\in \Gamma \Lambda ^0N$ let $K=\{ \underline{u}\in \breve{T}N: \dot{s}\vert _{\underline{u}}=1\}$. A *semi-spray* $X_K\in \Gamma \breve{T}K$ is a vector field given by

146 $$\begin{aligned} X_K=\frac{\partial \;}{\partial s}+ \dot{x}^{a} \frac{\partial \;}{\partial x^{a}}+ X_K^{a}(s,x^{a},\dot{x}^{a})\frac{\partial \;}{\partial \dot{x}^{a}}. \end{aligned}$$

Unlike a spray, there are no homogeneity conditions on $X_K^{a}$. The semi-spray $X_K$ corresponds to a set of ordinary differential equations locally expressed as

147 $$\begin{aligned} \frac{d^2f^{a}}{ds^2}= X_K^{a}\bigg ( s,f^{a}, \frac{df^{a}}{ds} \bigg ). \end{aligned}$$

Similarly to the case with a spray, *f*(*s*) is a solution to Eq. (147) if and only if its lift $\dot{f}(s)=(1,f^{a}(s), df^{a}(s)/ds)$ is an integral curve of $X_K$.

Observe that in the case where $K=E_{s}\subset U$, we may identify Eq. (146) with Eq. (35), a Vlasov field on a lab-time bundle Eq. (21).

Given such a semi-spray on *K* we may construct a spray on $\breve{T}N$ and vice versa. The full details of the lemma can be found in [26]. An example of this lemma in action is given below. If we are given a semi-spray determined by coefficients $X_K^{a}$ over *K* (equipped with a choice of parameterisation *s*), then we may construct a spray over $\breve{T}N$ with the following coefficients

148 $$\begin{aligned} {\left\{ \begin{array}{ll} {}& X^0(x,\dot{x})=0\\ {}& X^{a}(x,\dot{x})= \dot{x}^0\dot{x}^0 X_K^{a}(x^0,x^{a},\dot{x}^{a}/\dot{x}^0), \end{array}\right. } \end{aligned}$$

where $s=x^0$. Note that under this construction, $X_K$ is induced by *X*. This is an example of the quadratic extension described in Lemma 2.12. The freedom to choose $X^0$ in Eq. (148) roughly corresponds to the freedom to reparameterise the spray according to Eq. (145). In the instance where we restrict ourselves to a Vlasov field on a lab time, then Sect. can be identified with Eq. (148).

### Auxiliary lemmas

#### Lemma A.5

Let $X\in \Gamma TN,\; f\in \Gamma \Lambda ^0N$ be such that $df\ne 0$, and $f^{-1}\{0\}=K\subset N$ with $\Sigma _K:K\hookrightarrow N$. We have $X\vert _K\langle f \rangle =0$ if and only if there exists a unique $Y\in \Gamma TK$ such that $X\vert _K= \Sigma _K{}_*Y$ (i.e. *X* is tangent to *K*).

#### Proof

Suppose *X* is tangent to *K* then we have

$$\begin{aligned} X\vert _K\langle f \rangle = \Sigma _K{}_*Y\langle f \rangle = Y\langle f \circ \Sigma _K \rangle = Y\langle 0 \rangle =0. \end{aligned}$$

Suppose now that $X\vert _K\langle f \rangle =0$. If a suitable vector field exists it is unique by the injectivity of $\Sigma _K$. Let $\ell$ be the dimension of *N*. Since $df\ne 0$ there exists a local coordinate system $\{ x^0=f,x^1,\ldots ,x^{\ell -1} \}$ where we may express *X* as $X=X^\mu \partial _\mu ^{(x)}$, where $\mu =0,\ldots ,\ell -1$. Since $X\langle f\rangle =0$, we have $X^0=X\langle x^0\rangle =0$. In this coordinate system we also have $\Sigma _K: (x^0,\ldots ,x^{\ell -1})\mapsto (0,x^1,\ldots ,x^{\ell -1})$. This allows us to define a vector field $Y\in \Gamma TK$ where locally $Y=Y^a\partial _a^{(x)},\; Y^a=\Sigma _K^*X^a$, for $a=1,\ldots ,\ell -1$. It follows that $X\vert _K =\Sigma _K{}_*Y,$ and hence, *X* is tangent to *K*. $\hfill\square$

#### Lemma A.6

Given a spacetime manifold *M* with a pseudo-Riemann metric *g*, there exists a Vlasov field $W\in \Gamma TU$ constructed from a force equation involving a non-metric compatible connection $\hat{\nabla }$, i.e. in local coordinates $(x,\dot{x})$,

149 $$\begin{aligned} \begin{aligned} {}&\hat{\nabla }_{\dot{C}}\dot{C}= \widetilde{i_{\dot{C}}\mathcal {F}} \quad \text {and}\quad W= \dot{x}^\mu \partial _\mu ^{(x)}+ \left( g^{\mu \nu }\mathcal {F}_{\nu \rho }\dot{x}^\rho - \hat{\Gamma }^\mu _{\rho \nu } \dot{x}^\nu \dot{x}^\rho \right) \partial _\mu ^{(\dot{x})}, \end{aligned} \end{aligned}$$

where $\dot{C}=C_*(\partial _\tau )$ and $\tau$ is the chosen parameterisation of the trajectories, such that the integral curves of *W* will not lie on $E_{\text {H}}$.

#### Proof

Let $\nabla$ denote the Levi-Civita connection built from *g*. Since $\hat{\nabla }$ is non-metric compatible, it has non-vanishing non-metricity:

$$\begin{aligned} \hat{Q}= \hat{\nabla } g \ne 0. \end{aligned}$$

Letting $F_{\text {H}}$ denote the kinematic indicator of $E_{\text {H}}$. We observe that *W* is not tangent to $E_{\text {H}}$ since there exists $\dot{C}$ such that

$$\begin{aligned} \begin{aligned} W\vert _{\dot{C}}\langle F_{\text {H}}\rangle&= \dot{C}_*(\partial _\tau ) \langle F_{\text {H}}\rangle = \partial _\tau \langle F_{\text {H}}\circ \dot{C}\rangle = \partial _\tau C^*\big (g(\dot{C}, \dot{C})\big ) = \dot{C}\langle g(\dot{C},\dot{C}) \rangle = \hat{\nabla }_{\dot{C}}\big ( g(\dot{C},\dot{C})\big ) = \hat{Q}(\dot{C},\dot{C},\dot{C}) + g(\hat{\nabla }_{\dot{C}}\dot{C},\dot{C}) \\ &= \hat{Q}(\dot{C},\dot{C},\dot{C}) + g\left( \widetilde{i_{\dot{C}}\mathcal {F}},\dot{C}\right) = \hat{Q}(\dot{C},\dot{C},\dot{C}) + i_{\dot{C}}i_{\dot{C}}\mathcal {F} = \hat{Q}(\dot{C},\dot{C},\dot{C}) \ne 0, \end{aligned} \end{aligned}$$

where $\tau$ is a parameter and $\dot{C}={C_*(\partial _\tau )}$. Hence, *W* is not tangent to $E_{\text {H}}$ by Lemma A.5, and consequently, its integral curves will not remain on $E_{\text {H}}$. Conversely, if we replace $\hat{\nabla }$ with the Levi-Civita connection $\nabla$ in Eq. (149), then we may see that $W\vert _{\dot{C}}\langle F_{\text {H}}\rangle =0$ since $\nabla g=0$. Hence, *W* is tangent to $E_{\text {H}}$ and consequently its integral curves are confined to $E_{\text {H}}$. $\hfill\square$

#### Lemma A.7

Let *M* be a Minkowski spacetime manifold of dimension 2 and let $s \in \Gamma \Lambda ^0M$ define a lab-time function such that $E_{ s }$ defines a lab-time bundle [see Eq. (21)]. The null geodesics parameterised by the induced lab-time coordinate in general do not satisfy the geodesic equation. Consequently, the prolongations of lab-time parameterised curves must be expressed in terms of the pre-geodesic equation.

#### Proof

Let $C:\mathcal {I}\hookrightarrow M$ be a trajectory parameterised by $\tau \in \Gamma \Lambda ^0\mathcal {I}$. Then the prolongation satisfies

$$\begin{aligned} \dot{C}=C_*(\partial _\tau ), \quad \dot{C}\langle s \rangle = \frac{d ( s \circ C)}{d \tau } =1. \end{aligned}$$

Let (*t*, *x*) define coordinates on *M*. Null trajectories in Minkowski spacetime form straight lines, for example,

$$\begin{aligned} (t\circ C)(\tau )=(x\circ C)(\tau ). \end{aligned}$$

The above trajectory satisfies

$$\begin{aligned} \begin{aligned} \frac{\partial ( s \circ C)}{\partial \tau }&= \frac{\partial (x\circ C)}{\partial \tau } \frac{\partial s }{\partial x}+ \frac{\partial (t\circ C)}{\partial \tau } \frac{\partial s }{\partial t} = \frac{\partial (x\circ C)}{\partial \tau } \left( \frac{\partial s }{\partial x}+ \frac{\partial s }{\partial t}\right) =1 \end{aligned} \end{aligned}$$

By defining $X=x\circ C$ and $T=t\circ C,$ we can write

$$\begin{aligned} \begin{aligned} {}&\dot{X}= \left( \frac{\partial s }{\partial x}+ \frac{\partial s }{\partial t} \right) ^{-1} \frac{\partial \;}{\partial x}, \;\\ \dot{T}= \left( \frac{\partial s }{\partial x}+ \frac{\partial s }{\partial t} \right) ^{-1} \frac{\partial \;}{\partial t}. \end{aligned} \end{aligned}$$

It follows that

$$\begin{aligned} \begin{aligned} \nabla _{\dot{X}}\dot{X}\langle s \rangle = \frac{d\;}{d\tau }\left( \frac{\partial s }{\partial x}+ \frac{\partial s }{\partial t} \right) ^{-1} \frac{\partial s}{\partial x} \ne 0, \end{aligned} \end{aligned}$$

in general since there is no specific relationship between $s,\; t$ and *x*. The same result follows for $\dot{T}$. Hence, the null trajectories parameterised by the lab-time function cannot obey the geodesic equation, and they must obey a pre-geodesic equation.

If, however, we choose to parameterise the null geodesics by the Minkowski coordinate *t*, then the above equation does satisfy $\nabla _{\dot{X}}\dot{X}=\nabla _{\dot{T}}\dot{T}=0$. $\hfill\square$

#### Lemma A.8

Let (*M*, *g*) be a spacetime manifold, let *E* be a kinematic domain with a local coordinate system $(x^\mu ,\dot{x}^a)$, let $\Omega _E$ be the volume form as given by Eq. (60), and let $\theta _E$ be a particle density form built from $\Omega _E$. The stress–energy 3-form $\tau ^E_\alpha$ Eq. (101) is equivalent to the Einstein–Vlasov stress–energy tensor Eq. (102).

#### Proof

First observe that $\widehat{dx^\mu }\vert _{\underline{u}}=dx^\mu : \underline{u}= u^\mu =\dot{x}^\mu \vert _{\underline{u}}$. We may write $\theta _E$ as

$$\begin{aligned} \theta _E= f_E i_{W_E}\Omega _E= f_E\dot{x}^\mu i_\mu ^{(x)}\Omega _E+ f_E\varphi _E^a i_a^{(\dot{x})} \Omega _E, \end{aligned}$$

where $\varphi _E^a= W_E \langle \dot{x}^a \rangle$. Here we use the coordinate-based definition for the de Rham pushforward as seen in [33]. Since only forms with maximal degree in the fibre coordinates contribute to the de Rham pushforward, we have

$$\begin{aligned} \begin{aligned} (dx^\mu \wedge \tau ^E_{dx^\nu }) \vert _p&= dx^\mu \wedge \pi _\varsigma \big ( \widehat{dx^\nu } \theta _E \big )\big \vert _p = -\big (dx^\mu \wedge i_\rho ^{(x)} d^4x\big )\big \vert _p \int _{E_p} \widehat{dx^\nu } \dot{x}^\rho f_E \frac{\det (g)}{\dot{x}_0} d^3\dot{x} \\{}&= -d^4x\vert _p \int _{E_p} \dot{x}^\mu \dot{x}^\nu f_E \frac{\det (g)}{\dot{x}_0} d^3\dot{x}. \end{aligned} \end{aligned}$$

Since $\star 1= \sqrt{\det (g)} d^4x$ and $\star \star 1=-1$, we have for each $p\in M$

$$\begin{aligned} \star \left( dx^\mu \wedge \tau ^E_{dx^\nu } \right) \big |_p = \int _{E_p} \dot{x}^\mu \dot{x}^\nu f_E \frac{\sqrt{\det (g)}}{\dot{x}_0} d^3\dot{x}, \end{aligned}$$

so that Eq. (102) holds. $\hfill\square$

### Proofs

#### Proof of Lemma 3.6

Let $X_1,X_2$ and $Y_1,Y_2$ be specially related. We then have

$$\begin{aligned} \begin{aligned} X_1\wedge X_2&= \left( \alpha Y_1 +\beta Y_2\right) \wedge \left( \gamma Y_1+ \delta Y_2\right) = \alpha \delta Y_1\wedge Y_2 + \beta \gamma Y_2\wedge Y_1 = \left( \alpha \delta -\beta \gamma \right) Y_1\wedge Y_2 = Y_1\wedge Y_2. \end{aligned} \end{aligned}$$

Suppose $X_1\wedge X_2\ne 0$ then there exists a basis $\{X_1,\ldots ,X_n\}$ such that $X_i\wedge X_j\ne 0$ for $i\ne j,\; i,j=1,\ldots ,n$. We may then express

$$\begin{aligned} Y_1= \alpha ^i X_i,\quad Y_2=\beta ^i X_i, \end{aligned}$$

for scalar fields $\alpha ^i,\beta ^i\in \Gamma \Lambda ^0N$.

Hence, if we have $X_1\wedge X_2= Y_1\wedge Y_2$, we observe that

$$\begin{aligned} \begin{aligned} X_1\wedge X_2&= Y_1\wedge Y_2 = \alpha ^i X_i \wedge \beta ^j X_j = \big (\alpha ^1 \beta ^2- \alpha ^2 \beta ^1\big ) X_1\wedge X_2. \end{aligned} \end{aligned}$$

It follows that $\alpha ^1\beta ^2-\beta ^1\alpha ^2=1$. It remains to be shown that the other coefficients ($(\alpha ^i,\beta ^i)$ for $i>2$) vanish. Since the coefficients of the terms $X_i\wedge X_j$ for *i* or $j>2$ are vanishing, we have $\alpha ^i\beta ^j- \alpha ^j \beta ^i=0$ for *i* or $j>2$. We have the following set of equations:

150 $$\begin{aligned} {}&\alpha ^i \beta ^j=\beta ^i \alpha ^j, \quad \text{ for } i,j>2 ,\end{aligned}$$

151 $$\begin{aligned} {}&\alpha ^1 \beta ^j= \alpha ^j \beta ^1, \quad \text{ for } j>2 ,\end{aligned}$$

152 $$\begin{aligned} {}&\alpha ^2 \beta ^j= \alpha ^j \beta ^2, \quad \text{ for } j>2 . \end{aligned}$$

Define column vectors

$$\begin{aligned} A=\begin{pmatrix} \alpha ^3\\ \vdots \\ \alpha ^n \end{pmatrix}, \quad B=\begin{pmatrix} \beta ^3\\ \vdots \\ \beta ^n \end{pmatrix}. \end{aligned}$$

These column vectors satisfy the following relations

153 $$\begin{aligned} {}&\alpha ^1B= \beta ^1A,&\text{ by } \text{ eq. }(151) ,\end{aligned}$$

154 $$\begin{aligned} {}&\alpha ^2B=\beta ^2A,&\text{ by } \text{ eq. } (152) . \end{aligned}$$

By multiplying Eq. (153) by $\beta ^2$ and then applying Eq. (154), we observe that $\beta ^2 \alpha ^1 B= \beta ^2 \beta ^1 A= \beta ^1 (\beta ^2A)= \beta ^1(\alpha ^2 B),$ and hence, $0=(\beta ^2\alpha ^1-\beta ^1\alpha ^2)B=B$. Since at least one of $\beta ^1, \beta ^2$ is nonzero, we must also have $A=0$ by Eqs. (153) or (154). Hence, $X_1,X_2$ and $Y_1,Y_2$ are specially related. $\hfill\square$

#### Proof of Lemma 3.9

Since $\Phi$ is integrable, there exists $V,Z\in \Gamma TN$ such that $\Phi =V\wedge Z$ and $[V,Z]=\gamma V+\delta Z$. Let $\Phi$ have another representation $\Phi =X\wedge Y$. By Lemma 3.6, there exist scalar field $\lambda ,\sigma ,\rho ,\kappa \in \Gamma \Lambda ^0N$ such that

$$\begin{aligned} \begin{aligned} X&= \lambda V+\sigma Z, \text { and } Y= \rho V+\kappa Z. \end{aligned} \end{aligned}$$

The Lie bracket of *X* and *Y* satisfies

$$\begin{aligned} \begin{aligned} {[}X,Y]&= [\lambda V+\sigma Z, \rho V+ \kappa Z] = [\lambda V, \rho V]+ [\lambda V,\kappa Z]+ [\sigma Z, \rho V]+ [\sigma Z, \kappa Z]. \end{aligned} \end{aligned}$$

Consider a term containing *V* and *Z*, e.g.

$$\begin{aligned} \begin{aligned} {[}\lambda V, \kappa Z]&= \lambda V\langle \kappa \rangle Z -\kappa Z\langle \lambda \rangle V+ \lambda \kappa [V,Z] = \big (\lambda \kappa \gamma -\kappa Z\langle \lambda \rangle \big ) V+ \big (\lambda \kappa \delta + \lambda V\langle \kappa \rangle \big ) Z. \end{aligned} \end{aligned}$$

Consider a term containing two of the same types, e.g.

$$\begin{aligned} \begin{aligned} {[}\lambda V,\rho V]&= \lambda V\langle \rho \rangle V -\rho V\langle \lambda \rangle V +\lambda \rho [V,V] = \lambda V\langle \rho \rangle V -\rho V\langle \lambda \rangle V. \end{aligned} \end{aligned}$$

Hence, we may write

$$\begin{aligned} \begin{aligned} {[}X,Y]&= \lambda V\langle \rho \rangle V -\rho V\langle \lambda \rangle V + \big (\lambda \kappa \gamma -\kappa Z\langle \lambda \rangle \big ) V+ \big (\lambda \kappa \delta + \lambda V\langle \kappa \rangle \big ) Z + \big (\sigma \rho \gamma -\rho V\langle \sigma \rangle \big ) Z+ \big (\sigma \rho \delta + \sigma Z\langle \rho \rangle \big ) V\\ {}&\qquad + \sigma Z\langle \kappa \rangle Z -\kappa Z\langle \sigma \rangle Z\\&=\; \big ( \lambda V\langle \rho \rangle -\rho V\langle \lambda \rangle + \lambda \kappa \gamma -\kappa Z\langle \lambda \rangle +\sigma \rho \delta + \sigma Z\langle \rho \rangle \big ) V \\{}&\qquad + \big (\sigma Z\langle \kappa \rangle -\kappa Z\langle \sigma \rangle + \lambda \kappa \delta + \lambda V\langle \kappa \rangle + \sigma \rho \gamma -\rho V\langle \sigma \rangle \big ) Z\\&= \gamma ' V+ \delta ' Z \end{aligned} \end{aligned}$$

There exist $\lambda ',\sigma ',\rho ',\kappa '$ such that $V=\lambda ' X+\sigma 'Y$ and $Z=\rho ' X+\kappa 'Y$. These are guaranteed to exist since $\lambda \kappa -\sigma \rho =1$. It follows that

$$\begin{aligned} {[}X,Y]=\big (\gamma '\lambda '+\delta '\rho '\big )X+ \big (\gamma '\sigma '+\delta '\kappa '\big )Y. \end{aligned}$$

$$\hfill\square$$

#### Proof of Lemma 3.17

By Lemma 3.13, there exists a horizontal vector field $X\in \Gamma TU$ such that $\Psi =\mathcal {R}\wedge X$. Then $W_{crd }$ and *X* are related by

$$\begin{aligned} \begin{aligned} W_{crd }=&\frac{1}{2 F_{crd }} \left( \mathcal {R}\langle F_{crd }\rangle X - X\langle F_{crd }\rangle \mathcal {R} \right) = X-\frac{X\langle F_{crd }\rangle }{2 F_{crd }}\mathcal {R}. \end{aligned} \end{aligned}$$

To see that $\Psi =\mathcal {R}\wedge W_{crd },$ first suppose that $\Psi =\mathcal {R}\wedge X$ for some $X\in \Gamma TU;$ then, observe that

$$\begin{aligned} \begin{aligned} \mathcal {R}\wedge \frac{\Psi \langle F_{crd },\bullet \rangle }{2 F_{crd }}=&\mathcal {R}\wedge \bigg ( \frac{\mathcal {R}\langle F_{crd }\rangle }{2 F_{crd }}X- \frac{X\langle F_{crd }\rangle }{2 F_{crd }}\mathcal {R} \bigg ) =\mathcal {R}\wedge X =\Psi . \end{aligned} \end{aligned}$$

Define local coordinates $(x^\mu ,\dot{x}^\mu )$. To see that $W_{crd }$ is horizontal, observe that for any $f\in \Gamma \Lambda ^0M$ we have

$$\begin{aligned} \begin{aligned} W_{crd }\langle \pi ^*f\rangle =&\frac{\Psi \langle F_{crd },\pi ^*f \rangle }{2F_{crd }} =\sum _\mu \frac{\Psi \langle (\dot{x}^\mu )^2,\pi ^*f \rangle }{2F_{crd }} =\sum _\mu \frac{\dot{x}^\mu \Psi \langle \dot{x}^\mu ,\pi ^*f \rangle }{F_{crd }} =\sum _\mu \frac{(\dot{x}^\mu )^2\dot{f}}{F_{crd }}=\dot{f}. \end{aligned} \end{aligned}$$

To see that $W_{crd }$ is radially quadratic observe that for any $\underline{u}\in U,\; \lambda \in \mathbb {R},\; f\in \Gamma \Lambda ^0M$,

$$\begin{aligned} \begin{aligned} W_{crd }\vert _{\lambda \underline{u}} \langle \dot{f}\rangle&= \frac{\Psi \vert _{\lambda \underline{u}}\langle F_{crd },\dot{f} \rangle }{2F_{crd }\vert _{\lambda \underline{u}}} =\sum _\mu \left( \frac{\dot{x}^\mu }{F_{crd }} \right) \bigg \vert _{\lambda \underline{u}} \Psi \vert _{\lambda \underline{u}}\langle \dot{x}^\mu ,\dot{f} \rangle =\frac{\lambda }{\lambda ^2} \sum _\mu \left( \frac{\dot{x}^\mu }{F_{crd }} \right) \bigg \vert _{\underline{u}} \lambda ^3 \Psi \vert _{\underline{u}}\langle \dot{x}^\mu ,\dot{f} \rangle \\ =&\lambda ^2 \frac{\Psi \vert _{\underline{u}}\langle (\dot{x}^\mu )^2,\dot{f}\rangle }{2F_{crd }\vert _{\underline{u}}} =\lambda ^2W_{crd }\vert _{\underline{u}}\langle \dot{f}\rangle . \end{aligned} \end{aligned}$$

Hence, $W_{crd }$ is a Vlasov field. $\hfill\square$

#### Proof of Lemma 4.5

Let *F* be the 1-homogeneous kinematic indicator for $E\subset U^+$. Define a foliation on $U^+$ of kinematic domains $E_\ell$ for $\ell \in \mathbb {R}^+$ where $E_1=E$ and $F|_{E_\ell }=\ell$. We may define a coordinate system $(x^\mu ,\ell ,\xi ^a)$ for $U^+$ where $\ell$ is constant on each $E_\ell$. We may choose $\xi ^a$ such that they are 0-homogeneous, and $\ell$ is 1-homogeneous by the 1-homogeneity of *F*. By Lemma 2.6, we have for each $\lambda >0$ and $\underline{u}\in U^+$,

155 $$\begin{aligned} W\vert _{\lambda \underline{u}}\langle x^\mu \rangle = \lambda W\vert _{\underline{u}}\langle x^\mu \rangle , \; W\vert _{\lambda \underline{u}}\langle \xi ^a\rangle = \lambda W\vert _{\underline{u}}\langle \xi ^a\rangle . \end{aligned}$$

The map

$$\begin{aligned} \Xi _\ell :E\rightarrow E_\ell \;; \;\Xi _\ell (\underline{v})=\ell \underline{v}, \end{aligned}$$

is well defined since $E_\ell =\{ \ell \underline{v},\; \underline{v}\in E\}$. Furthermore, it satisfies

$$\begin{aligned} \Pi _E\circ \Sigma _{E_\ell }\circ \Xi _\ell = \mathbb {1}_E \; \text{ i.e. } \; \Xi _\ell =\left( \Pi _E\circ \Sigma _{E_\ell } \right) ^{-1}. \end{aligned}$$

Observe that we have

$$\begin{aligned} \begin{aligned} {}&\Xi _\ell ^*x^\mu = x^\mu , \quad \Xi _\ell ^*\xi ^a=\xi ^a,\quad {}\Xi _\ell ^*dx^\mu =dx^\mu , \quad \Xi _\ell ^*d\xi ^a=d\xi ^a. \end{aligned} \end{aligned}$$

Lastly, define $\Omega \in \Gamma \Lambda ^{2n-1}U$ by

$$\begin{aligned} \Omega =dx^0\wedge \cdots \wedge dx^{n-1}\wedge d\xi ^1\wedge \cdots \wedge d\xi ^{n-1}. \end{aligned}$$

First observe that $d\theta ^+=0$ since the exterior derivative commutes with the pullback. Notice also that $i_{\mathcal {R}}\theta ^+=0$ since $\Pi _E{}_*\mathcal {R}=0$ we have

$$\begin{aligned} i_{\mathcal {R}}\theta ^+= i_{\mathcal {R}} \Pi _E^*\theta _E= \Pi _E^*\left( i_{\Pi _E{}_*\mathcal {R}}\theta _E\right) =0. \end{aligned}$$

To see that $i_W\theta ^+=0$ define coordinate functions $y^0,\ldots ,y^{2n-2}$ as $y^k=x^k$ for $0\le k\le n-1$, $y^k=v^{k-n+1}$ for $n\le k \le 2n-2$. Hence, $(y^0,\ldots ,y^{2n-2})$ is a coordinate system for *E* while $(\ell ,y^0,\ldots ,y^{2n-2})$ is a coordinate system for $U^+$. With appropriate domains $y^k = \Pi _E^*y^k$ and $y^k = \Sigma _E^*y^k$. Hence, for $\Sigma _E\circ \Pi _E:U^+\rightarrow U^+$ we have $(\Sigma _E\circ \Pi _E)^*(y^k)=y^k$. Thus, $\Omega =dy^0\wedge \cdots \wedge dy^{2n-2}$. Also,

$$\begin{aligned} (\Sigma _E\circ \Pi _E)^*f = f \qquad \text {and}\qquad (\Sigma _E\circ \Pi _E)^*i^{(y)}_k \Omega = i^{(y)}_k \Omega \end{aligned}$$

where *f* is a scalar which such that $\mathcal {R}\langle f\rangle =0$, so it is a function only of $(y^0,\ldots ,y^{2n-2})$.

Since we have, for any $\underline{v}\in E$,

$$\begin{aligned} W\vert _{\lambda \underline{v}} \langle y^k\rangle = \lambda W\vert _{\underline{v}} \langle y^k\rangle \end{aligned}$$

by eq. (155). Thus, for $\underline{v}\in E$ (and $\lambda \underline{v}\in E_\lambda$)34 we have

$$\begin{aligned} \begin{aligned} \Big (\Xi _\lambda ^*\left( i_{W_{E_\lambda }} dy^k\right) \Big ) \Big \vert _{\underline{v}}&= \; \Xi _\lambda ^*\Big (\left( i_{W_{E_\lambda }} dy^k\right) \vert _{\lambda \underline{v}}\Big ) =i_{W_{E_\lambda }} dy^k\vert _{\lambda \underline{v}} = \lambda i_{W_E} dy^k\vert _{\underline{v}} = \lambda i_{W_E} \Xi _\lambda ^*\left( dy^k\vert _{\lambda \underline{v}} \right) = \Big (\lambda i_{W_E} \Xi _\lambda ^*\left( dy^k\right) \Big )\Big \vert _{\underline{v}}. \end{aligned} \end{aligned}$$

Since this is true for any $\underline{v}\in E,$ we have (for $dy^k\in \Gamma \Lambda ^1 E_\lambda$)

$$\begin{aligned} \Xi _\lambda ^*\left( i_{W_{E_\lambda }} dy^k\right) = \lambda i_{W_E}\Xi _\lambda ^*dy^k. \end{aligned}$$

It follows that

$$\begin{aligned} \lambda i_{W_{E}} \Xi _\lambda ^*\Sigma _{E_\lambda }^*\Omega = \Xi _\lambda ^*\left( i_{W_{E_\lambda }} \Sigma _{E_\lambda }^*\Omega \right) . \end{aligned}$$

Since $\theta _E$ is an $(2n-2)$-form on a $(2n-1)$-dimensional manifold *E*, with coordinates $(y^0,\ldots ,y^{2n-2}),$ we may write

$$\begin{aligned} \theta _E=\theta _E^k i_k^{(y)}\Sigma _E^*\Omega , \end{aligned}$$

where $\theta ^\mu _E=\theta ^\mu _E(y^0,\ldots ,y^{2n-2})$. Thus, we can extend $\theta ^\mu _E$ to a scalar field on $U^+$ so that $\mathcal {R}\langle \theta _E^k\rangle =0$. Also $\theta ^k_E=\Pi _E^*\theta _E^k=\Xi ^*_\ell \theta _E^k$ for the appropriate domains. Therefore,

$$\begin{aligned} \begin{aligned} \theta ^+&= \Pi _E^*\theta _E\\ = \Pi _E^*\left( \theta _E^k i_k^{(y)}\Sigma _E^*\Omega \right) = \Pi _E^*\theta _E^k\;(\Sigma _E\circ \Pi _E)^*\left( i_k^{(y)}\Omega \right) = \theta _E^k\; i_k^{(y)}\Omega . \end{aligned} \end{aligned}$$

We then have for each $\lambda$

$$\begin{aligned} \begin{aligned} \Xi _\lambda ^*\left( \Sigma _{E_\lambda }^*\left( i_W\theta ^+\right) \right)&= \Xi _\lambda ^*\left( i_{W_{E_\lambda }} \Sigma _{E_\lambda }^*\Pi _E^*\theta _E \right) = \; \Xi _\lambda ^*\left( i_{W_{E_\lambda }} \Sigma _{E_\lambda }^*\Pi _E^*\left( \theta _E^k i_k^{(y)}\Sigma _E^*\Omega \right) \right) = -\theta _E^k i_k^{(y)} \Xi _\lambda ^*\left( i_{W_{E_\lambda }} \Sigma _{E_\lambda }^*\Pi _E^*\Sigma _E^*\Omega \right) \\ &= -\theta _E^k i_k^{(y)} \Xi _\lambda ^*\left( i_{W_{E_\lambda }} \Sigma _{E_\lambda }^*\Omega \right) = -\lambda \theta _E^k i_k^{(y)} i_{W_{E}} \Xi _\lambda ^*\left( \Sigma _{E_\lambda }^*\Omega \right) =\; \lambda i_{W_E}\Xi _\lambda ^*\left( \theta _E^k i_k^{(y)} \Sigma _{E_\lambda }^*\Omega \right) \\ &=\; \lambda i_{W_E}\Xi _\lambda ^*\left( \Sigma _{E_\lambda }^*\Pi _E^*\left( \theta _E^k i_k^{(y)}\Sigma _E^*\Omega \right) \right) = \;\lambda i_{W_{E}} \Xi _\lambda ^*\left( \Sigma _{E_\lambda }^*\Pi _E^*\theta _E \right) = \lambda \ i_{W_E} \theta _E =0 \end{aligned} \end{aligned}$$

Since $\Xi _\lambda$ is bijective, we have $\Sigma _{E_\lambda }^*\left( i_W\theta ^+ \right) =0$. In order to see that this implies $i_W\theta ^+=0$, observe the following result:

$$\begin{aligned} \begin{aligned} {}&\hbox {Given} \alpha \in \Gamma \Lambda ^{2n-3} U^+ \hbox {, if} i_{\mathcal {R}} \alpha =0 \text { and }\Sigma _{E_\lambda }^*\alpha =0\text { for each }\lambda >0\text {, then }\alpha =0. \end{aligned} \end{aligned}$$

This follows since $i_{\mathcal {R}} \alpha =0$, we may write $\alpha = \alpha ^{kj}i_k^{(y)}i_j^{(y)} \Omega$. For any $\lambda >0,$ $0=\Sigma _{E_\lambda }^*\alpha =\Sigma _{E_\lambda }^*\left( \alpha ^{kj} \right) i_k^{(y)}i_j^{(y)} \Sigma _{E_\lambda }^*\Omega$. Since the form on the RHS is nonzero, we must have $\Sigma _{E_\lambda }^*\left( \alpha ^{kj} \right) =0$ for all $\lambda >0$. Since $U^+$ is the union of $E_\lambda ,$ then $\alpha ^{jk}=0$. It follows that $(\Psi ,\theta ^+)$ satisfy the transport equations on $U^+$. $\hfill\square$

#### Proof of Lemma 4.6

First suppose that $\theta ^+=\Pi _E^*\theta _E$. We have $\Sigma _E^*\theta ^+= \Sigma _E^*\Pi _E^*\theta _E= \left( \Pi \circ \Sigma _E \right) ^*\theta _E= \theta _E$.

Suppose we now have $\theta _E= \Sigma _E^*\theta ^+$. Define a coordinate system $(\ell ,y^k)$ in the same way as in the proof for Lemma 4.5, so that $\left( \Sigma _E\circ \Pi \right) ^*\! y^k=y^k$. Since $d\theta ^+=0$ and $i_{\mathcal {R}}\theta ^+=0$ we have $\theta ^+= \theta ^k i_k^{(y)} \Omega _X$, where $\theta ^+$ is a function of $(y^0,\ldots ,y^{2n-2})$, and $\Omega _X=dy^0\wedge \cdots \wedge dy^{2n-2}$. We then have

$$\begin{aligned} \begin{aligned} \Pi _E^*\theta _E&= \Pi _E^*\Sigma _E^*\theta ^+ = \Pi _E^*\Sigma _E^*\left( \theta ^k i_k^{(y)} \Omega _X \right) = \theta ^k \left( \Sigma _E\circ \Pi _E \right) ^*\left( i_k^{(y)} \Omega _X \right) = \theta ^k i_k^{(y)} \Omega _X = \theta ^+. \end{aligned} \end{aligned}$$

$$\hfill\square$$

#### Proof of Lemma 4.11

Define a coordinate system on *N* by $(x^0,\ldots ,x^{\ell -2},t)$ such that $X=\partial _t$. Since $i_X\alpha =0,$ it follows that $\alpha =\alpha _0 \text {d}x^0\wedge \cdots \wedge \text {d}x^{\ell -2}$ for some $\alpha _0\in \Gamma \Lambda ^0N$. Let $\beta _0=i_X\beta ,$ then we have

$$\begin{aligned} \begin{aligned} \int _N\alpha \wedge \beta&= \int _N \alpha _0 \text {d}x^0\wedge \cdots \wedge \text {d}x^{\ell -2} \wedge (i_X\beta )\text {d}t\\&= \int _{(x^0,\ldots ,x^{\ell -2})\in K_{t_0}} \int _{t\in \mathbb {R}} \alpha _0(x^0,\ldots ,x^{\ell -2}) \text {d}x^0 \wedge \text {d}x^{\ell -2} \wedge \beta _0(x^0,\ldots ,x^{\ell -2},t)\text {d}t\\&= \int _{(x^0,\ldots ,x^{\ell -2})\in K_{t_0}} \alpha _0(x^0,\ldots ,x^{\ell -2}) \text {d}x^0\wedge \cdots \wedge \text {d}x^{\ell -2} \int _{t\in \mathbb {R}} \beta _0(x^0,\ldots ,x^{\ell -2},t)\text {d}t\\&= \int _{(x^0,\ldots ,x^{\ell -2})\in K_{t_0}} \Sigma _{t_0}^*\alpha \int _{t\in \mathbb {R}}\beta \vert _{(x^0,\ldots ,x^{\ell -2},t)} = \int _{(x^0,\ldots ,x^{\ell -2})\in K_{t_0}} \Sigma _{t_0}^*\alpha \left( \int _{\mathbb {R}} \eta _{(x^0,\ldots ,x^{\ell -2},t)}^*\beta \right) . \end{aligned} \end{aligned}$$

$$\hfill\square$$

## Footnotes

1 Recall, examples from Finsler geometry may be ignored for readers unfamiliar with this geometry. For this reason, we do not give definitions of standard Finsler objects.
